# Design and Synthesis of Novel Aminoindazole-pyrrolo[2,3-*b*]pyridine Inhibitors of IKKα That Selectively Perturb Cellular Non-Canonical NF-κB Signalling

**DOI:** 10.3390/molecules29153515

**Published:** 2024-07-26

**Authors:** Christopher Riley, Usama Ammar, Aisha Alsfouk, Nahoum G. Anthony, Jessica Baiget, Giacomo Berretta, David Breen, Judith Huggan, Christopher Lawson, Kathryn McIntosh, Robin Plevin, Colin J. Suckling, Louise C. Young, Andrew Paul, Simon P. Mackay

**Affiliations:** 1 Strathclyde Institute of Pharmacy and Biomedical Sciences, University of Strathclyde, 161 Cathedral Street, Glasgow G4 0RE, UK; 2 Department of Pure and Applied Chemistry, University of Strathclyde, 295 Cathedral Street, Glasgow G1 1XL, UK

**Keywords:** inhibitory κB kinases, IKKα, nuclear factor-κB (NF-κB), non-canonical NF-κB signalling, IKKα inhibitor

## Abstract

The inhibitory-kappaB kinases (IKKs) IKKα and IKKβ play central roles in regulating the non-canonical and canonical NF-κB signalling pathways. Whilst the proteins that transduce the signals of each pathway have been extensively characterised, the clear dissection of the functional roles of IKKα-mediated non-canonical NF-κB signalling versus IKKβ-driven canonical signalling remains to be fully elucidated. Progress has relied upon complementary molecular and pharmacological tools; however, the lack of highly potent and selective IKKα inhibitors has limited advances. Herein, we report the development of an aminoindazole-pyrrolo[2,3-*b*]pyridine scaffold into a novel series of IKKα inhibitors. We demonstrate high potency and selectivity against IKKα over IKKβ in vitro and explain the structure–activity relationships using structure-based molecular modelling. We show selective target engagement with IKKα in the non-canonical NF-κB pathway for both U2OS osteosarcoma and PC-3M prostate cancer cells by employing isoform-related pharmacodynamic markers from both pathways. Two compounds (**SU1261** [IKKα *K*_i_ = 10 nM; IKKβ *K*_i_ = 680 nM] and **SU1349** [IKKα *K*_i_ = 16 nM; IKKβ *K*_i_ = 3352 nM]) represent the first selective *and* potent pharmacological tools that can be used to interrogate the different signalling functions of IKKα and IKKβ in cells. Our understanding of the regulatory role of IKKα in various inflammatory-based conditions will be advanced using these pharmacological agents.

## 1. Introduction

The Nuclear Factor-κB (NF-κB) family of transcription factors are central coordinators of the innate and adaptive immune response and play key roles in cancer development and progression [1,2,3,4,5,6,7,8]. NF-κB complexes also have a major function in controlling the ability of both pre-neoplastic and malignant cells to resist apoptosis and so contribute to cell survival and the development of recognised hallmarks of cancer [1,3,4]. These NF-κB signalling pathways and the protein components that regulate them remain attractive targets for new therapeutic interventions.

The cellular activation of NF-κB pathways, which are regulated by the inhibitory κB kinases (IKKs), is elevated when homeostasis is disrupted. Increased or constitutive IKKα/β catalytic activity leads to enhanced NF-κB expression and raised nuclear localisation, which in turn, results in NF-κB complexes engaging specific NF-κB-specific binding elements in the promoter regions of target genes to drive transcriptional activity. The genes transcribed support the cellular responses associated with inflammation and immune responses but also tumour survival and progression [1,2,3,4,5,6,7,8].

The IKKs are the key upstream regulators of the NF-κBs, which exist as either inactive homo- or hetero-dimers bound to inhibitory kappa B proteins (IκBs) or IκB-like domains within their own structures [1,2]. In the canonical NF-κB pathway, IKK activation results in the phosphorylation, targeted ubiquitination, and proteolytic removal of IκB proteins to liberate active NF-κB1 homo- and/or hetero-dimers that translocate to the nucleus. Conversely, in the non-canonical NF-κB pathway, phosphorylation targets the IκB-like C-terminal region of the high molecular weight p100 NF-κB2, which is followed by proteolytic processing to generate a different cohort of NF-κB homo/hetero-dimers [1,2,3,4,5,6,7,8].

Several studies have indicated that IKKα and IKKβ play key but divergent roles in the regulation of global NF-κB signalling and many aspects of cellular transcription [9,10]. IKKβ regulates the activation of the canonical NF-kB pathway via activation of p65 RelA-p50 heterodimers [11,12,13], leading to the generation of multiple pro-inflammatory mediators in a variety of cell types, which can support disease progression. Whilst IKKα has been shown to have a lesser role in the canonical NF-κB pathway [10,12], it is pivotal in the activation of the non-canonical NF-κB pathway, catalysing the phosphorylation and proteolytic processing of p100 NF-κB2, which in turn liberates distinct NF-κB dimers, typically p52/RelB complexes, and initiates the transcription of a specific subset of genes. As IKKα and IKKβ have specific cellular functions [9,14,15], the selective inhibition of one isoform over the other would represent divergent approaches to new therapeutic interventions in inflammatory-based diseases and cancer.

In relation to IKKβ-mediated canonical NF-κB signalling, concerted efforts have been made to develop several selective small-molecule kinase inhibitors of IKKβ [16,17,18] primarily focused on delivering clinical agents to treat inflammatory conditions such as asthma, arthritic disease, and gastrointestinal conditions [3,19,20]. However, efforts in this area have been suspended due to the emergence of adverse effects associated with the inhibition of cellular IKKβ catalytic activity, including inflammatory skin disease and the development of increased permeability and sensitisation of the colonic epithelium to a range of insults [21]. Perhaps not surprisingly, intestinal and liver toxicity alongside immunosuppression and susceptibility to infection have also been reported in a limited number of phase I/II clinical trials of IKKβ inhibitors [3], which has further limited their clinical applications. Whilst IKKβ knockout mice display severe liver dysfunction [22], murine transgenic ‘knock-in’ models that express a non-activatable form of IKKα^AA/AA^ (Ser176/180Ala) appeared normal, healthy, fertile, and unaffected by the gross toxicities associated with inhibition of IKKβ [23]. This suggests that selective targeting of IKKα pharmacologically could avoid the deleterious outcomes associated with targeting cellular IKKβ and the associated dose-limiting toxicity.

We have previously reported the first series of IKKα kinase inhibitors with clear selectivity over IKKβ, developed around a pyrrolo[2,3-*d*]pyrimidine scaffold, but with moderate potency in cells [24]. The series represented an advance beyond limited examples in the patent literature, where little detail regarding activity and specificity for IKKα has been reported [25]. The natural products noraristeromycin [26] and apigenin [27] have been identified as potential IKKα inhibitors, with reported inhibitory action against IKK-NF-κB signalling. However, the effect of these compounds could not be ascribed solely to IKKα inhibition because both are known to be promiscuous kinase inhibitors. Moreover, selective pharmacodynamic readouts to distinguish IKKα- versus IKKβ-regulated signalling were not deployed in the cancer models used [4,26,27,28,29], and apigenin is also known to have effects on cancer-related signalling and phenotypic endpoints via the PI3-kinase/Akt axis [30].

Given the substantial evidence now suggesting that IKKα has an important role in a number of cancers [4] including inflammatory-driven solid tumour types such as prostate [31,32,33], colorectal [34,35], breast [23,36,37], and pancreatic [38,39,40], and in certain haematological tumours [4], there is a demand for selective IKKα inhibitors to dissect and validate its regulatory roles in tumour development and progression and establish its potential as a pharmacological target.

The most potent and selective inhibitor from our first series (**SU909**) [24] had a high total polar surface area (134.36 Å^2^), which accounted for its low cellular potency. Herein we describe the design, synthesis, and evaluation of a second series of inhibitors based on an alternative aminoindazole/pyrrolo[2,3-*b*]pyridine scaffold in our ongoing programme to develop isoform-selective IKKα inhibitors. We present a structure-based design to successfully generate highly potent and selective inhibitors. We translate this selective biochemical activity into both osteosarcoma and prostate cancer cells using the relevant isoform-related pharmacodynamic markers. Notably, we show that two exemplars from our series (**SU1261** and **SU1349**) selectively perturb non-canonical NF-κB signalling whilst avoiding any significant impact upon the IKKβ-mediated canonical NF-κB pathway in cells. These first-in-class inhibitors, therefore, represent primary selective *and* potent pharmacological tools that can be used to interrogate the signalling function of IKKα in cells. We propose that the research community that is focused on the regulatory role of IKKα in various cancers, cardiovascular conditions, and inflammatory-based diseases can gain a greater understanding of the NF-kB signalling pathways using these pharmacological agents.

## 2. Results and Discussion

### 2.1. Inhibitor Design and Structure–Activity Relationship

**Figure 1:**
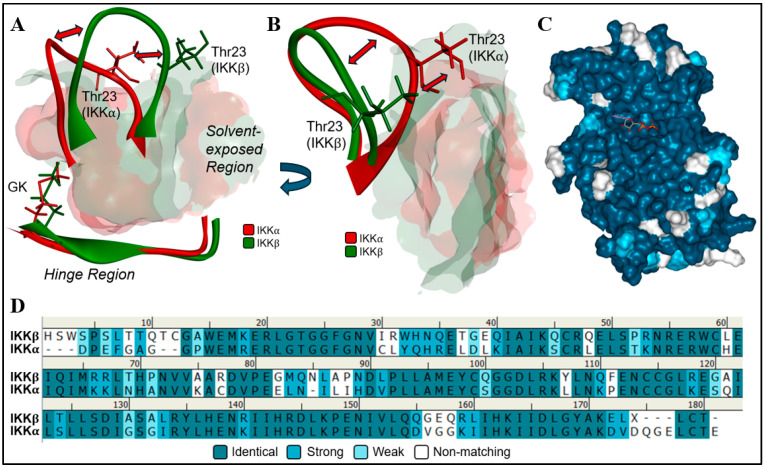
Superimposition of the IKKα (red) and IKKβ (green) ATP-binding sites taken from 5EBZ.pdb and 4KIK.pdb, respectively. (**A**) Top view of IKKα superimposed onto IKKβ binding site showing dislocation of the IKKβ G-loop containing Thr23 (green) from the IKKα position (red), indicated by the double-headed red arrow; (**B**) 90° rotation to show the front view of both superimposed isoforms, with the twisted Thr23-containing G-loop of IKKβ displaced towards the N-lobe above the ATP binding site (double-headed red arrow). (**C**). Structure of IKKβ with levels of similarity with IKKα (rmsd 1.393 Å^2^)) colour-coded as indicated. The ATP analogue marks the ATP binding site and is surrounded by identical residues. (**D**) Sequence alignment of ATP binding site amino acid residues of both IKKα and IKKβ, with levels of similarity colour-coded as indicated.

To design ligands with selectivity for IKKα over IKKβ, the ATP-binding sites of the two isoforms were superimposed and compared to identify specific differences that could be exploited. When aligning the primary sequences in this region (residues 6–180, IKKα; and 1–180, IKKβ), it is striking to see the level of homology between the two isoforms: 62.8% sequence identity and 77.2% sequence similarity, with both having Met as the GK residue and GK+1/GK+3 as Glu and Cys, respectively (Figure 1). However, given the number of reported structurally diverse compounds that inhibit IKKβ over IKKα [4,16,17,18,20,25,41], there are clearly differences in the two ATP-binding sites to impart such selectivity. To explore these differences, the kinase domain of IKKβ using the 4KIK.pdb Chain B crystal structure [42] that contains two phosphorylated Ser residues in its activation loop was compared with the equivalent domain from IKKα taken from the 5EBZ.pdb coordinates (Figure 1). To date, no group has been able to successfully crystallise IKKα and report a high-resolution structure, but Polley and co-workers [43] have generated structures of IKKα in dimeric (~150 kDa) and hexameric (~450 kDa) forms using a combination of X-ray crystallography and single-particle cryoelectron microscopy to 4.5 Å resolution. This was achieved using a recombinant form of the protein, where both Ser amino acids in the activation loop had been mutated to Glu residues, thus representing a constitutively activated form of the kinase that could be superimposed with the IKKβ 4KIK crystal structure. Whilst the resolution of the IKKα structure is lower than IKKβ, encouragingly, it mapped very well with the homology model we had previously reported to successfully guide the structure-based inhibitor design of the SU909 series [24].

**Figure 2:**
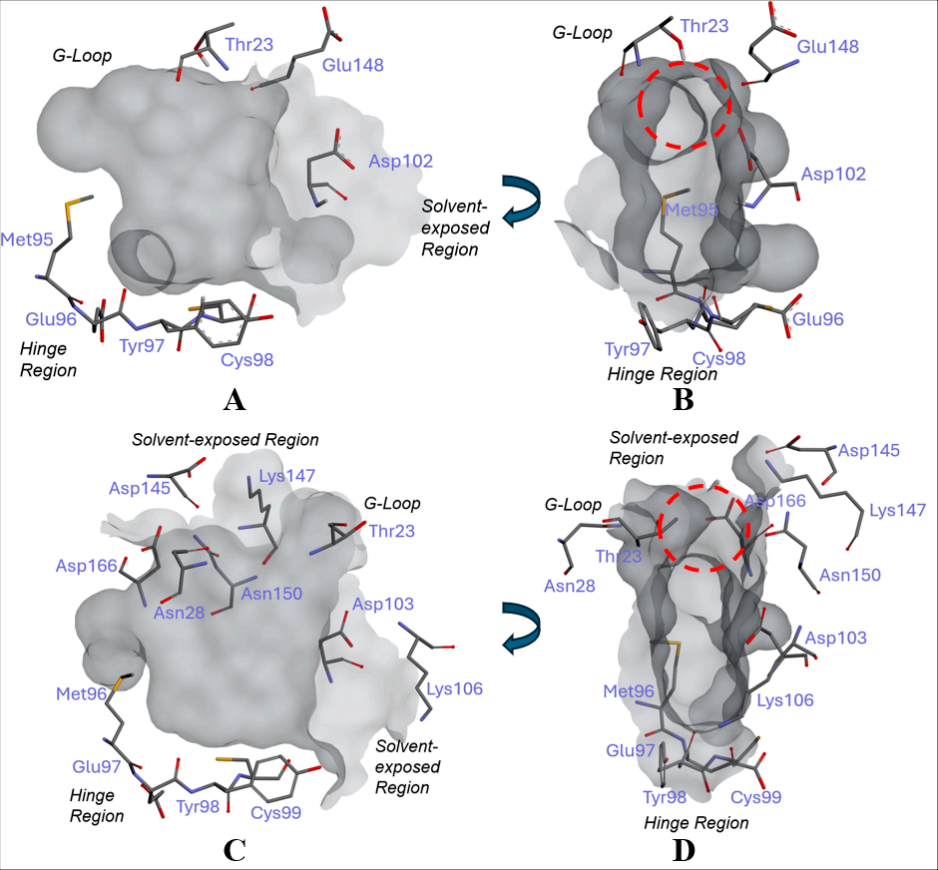
The 3D crystal structures of the ATP binding sites of both IKKα and IKKβ (PDB ID: 5EBZ and 4KIK, respectively). The grey surface represents the solvent-accessible surface in the ATP-binding state. H atoms: white; C atoms: grey; N atoms: blue; O atoms: red; S atoms yellow. (**A**) Top view of the IKKα binding site showing the hinge region at the base, with the GK, GK+1, and GK+3 highlighted (Met95, Glu96 and Cys98, respectively), and the G-loop segment at the top to form a wall opposite the hinge, with Thr23 and Glu148 highlighted; (**B**) rotation by 90° to reveal the front view of the IKKα binding site from the solvent-accessible area, with the hinge region at the base. Asp102 is the principal residue at the lip of this region available for ligand binding; (**C**) top view of the IKKβ binding site showing the hinge region at the base and in the same position as IKKα, with Met96, Glu97, and Cys99 highlighted. Thr23 and Asn28 from the G-loop are labelled, along with residues that line the lips of the two solvent-exposed regions. (**D**) Rotation by 90° to reveal the front view of the IKKβ binding site, again with the hinge region at the bottom. The red circle highlights the obstructive bulge from Thr23 that must be negotiated to access the revealed solvent region and the large number of residues that are presented at the lips of both solvent-exposed areas (Asn28, Asp103, Asp145, Lys147, Asn150, and Asp166). The red circle in B shows that the equivalent position of Thr23 in IKKα does not protrude into the site but instead forms the back wall to block off solvent access from the top completely.

Despite the high sequence homology, a key difference between the two isoforms involves the Thr23-containing G-loop that is opposite the hinge-binding region. By adopting different positions, it imposes a dissimilar topography in the sites (Figure 1): In IKKα, the G-loop forms an intact wall to enclose the site on three sides (the third involving the Met95 GK residue shown on the left in Figure 1A), which offers additional binding sites to a ligand (Figure 2A,B); in IKKβ, this sequence is twisted and displaced to the upper section of the binding pocket (Figure 2C), which removes the wall from the opposite side of the hinge-binding region seen in IKKα and exposes the site to solvent on two sides. Whilst hydrophilic groups could potentially be accommodated in this solvent region to promote binding with IKKβ, the occlusion wall in IKKα offers putative interaction sites that could be exploited to favour binding with the latter isoform. Furthermore, the twisting of the G-loop in IKKβ to open the site to solvent results in Thr23 encroaching into the site itself to create an obstructive bulge that any ligand moiety accessing the solvent area would need to negotiate (Figure 2D). Finally, because IKKβ is open on two sides, there is a greater number of residues proximal to the site interior available for ligand binding (Asn28, Asp103, Asp145, Lys147, Asn150, and Asp166, Figure 2C) compared to that of IKKα, which only has one accessible residue; Asp102, Figure 2B). In summary, IKKα has a more enclosed site, only open on one side to solvent adjacent to the hinge, whereas IKKβ is more open to solvent on two sides, although with a Thr23 protrusion that interrupts contiguous solvent access from both (Figure 2D).

**Figure 3:**
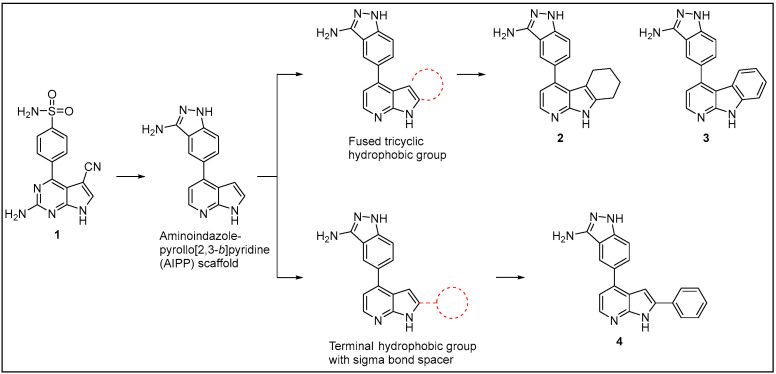
Rationale for the design of the preliminary test set of AIPPs used in this study. Tricyclic derivatives of the AIPP-based scaffold with fused hydrophobic groups (red circle) were designed to study the effect of co-planarity on binding (**2** and **3**). A derivative with a sigma bond spacer separating the hydrophobic group (red circle) from the AIPP was designed to assess flexibility (**4**).

**Figure 4:**
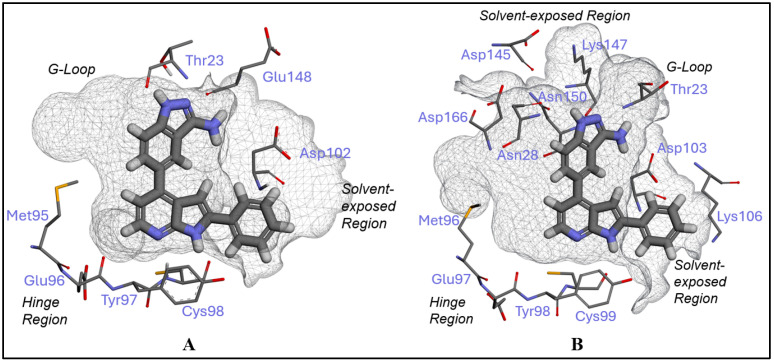
The predicted binding poses for **4** with IKKα (**A**) and IKKβ (**B**). The wire mesh represents the solvent-accessible surface in the ATP-binding state. H atoms: white; C atoms: grey; N atoms: blue; O atoms: red; S atoms yellow. (**A**) The pyrrolo[2,3-b]pyridine binding motif was directed to the hinge region and exhibited two H-bond interactions with Cys98 (2.02 and 2.14 Å). The aminoindazole binding motif was anchored to the G-loop by two H-bond interactions with Thr23 (2.38 Å) and Glu148 (2.21 Å) that make up the occluded wall. The 2-phenyl ring was exposed to the solvent area and exhibited hydrophobic interactions with the non-polar amino acid residues at this site (Leu21 and Val151). (**B**) In the IKKβ active site, **4** showed a similar binding pose to that of IKKα. The pyrrolo[2,3-b]pyridine ring had two interactions with Cys99 (2.58 and 2.72 Å) in the hinge region, but whilst the aminoindazole was orientated towards the G-loop, it only interacted with Asp103 (2.48 A) in the solvent-exposed area, which was occluded in IKKα.

In 2008, the aminoindazole-pyrrolo[2,3-*b*]pyridine (AIPP) core scaffold was identified as an inhibitor of both IKKα and IKKβ but without revealing its potency, other than to state sub-micromolar activity was observed against both isoforms in a cell-free, time-resolved FRET assay [25,44]. In 2017, we identified that compound **1** (**SU909**), a pyrrolo[2,3-d]pyrimidine, was a selective inhibitor of IKKα that recapitulated this discrimination in U2OS cells [24]. To examine the pyrrolo[2,3-*b*]pyridines as an alternative scaffold, we adopted the AIPP core because it had similar dimensions to **SU909** (**1**), with HBD/HBA motifs positioned at the scaffold extremities to interact with both the hinge region and the G-loop wall of IKKα, a rationale we had previously proposed as the basis for imparting selectivity. A preliminary set of AIPP derivatives was designed and synthesised to explore its utility as an IKK-targeting scaffold, whilst incorporating additional functionality through which to introduce selectivity for the IKKα isoform (**2**–**4**; Figure 3). This was achieved by initially adopting two different approaches: either appending a hydrophobic moiety to the pyrrolo[2,3-*b*]pyridine motif as a fused form to afford the tricyclic derivatives **2** and **3**, or as a phenyl substituent to introduce flexibility with respect to the central pyrrolo[2,3-*b*]pyridine moiety in **4**. When assessed against IKKα and IKKβ using our in-house DELFIA kinase assay [24] (Table 1), all three compounds showed excellent inhibitory activity against IKKα (*K_i_* 2–3 nM), which was significantly more potent than our previous hit (SU909: *K_i_* 80 nM). However, unlike **SU909**, they had poor selectivity, inhibiting IKKβ at low nanomolar concentrations (*K_i_* 5–77 nM). This comparable activity against both isoforms could be explained by our docking studies, with all three compounds adopting similar poses in the ATP binding site of both IKKs. The pyrrolo[2,3-*b*]pyridine was bound to the hinge region in the classical HBA/HBD motif with the GK+3 amide backbone, whilst the aminoindazole projected towards the top of the pocket to interact with the G-loop (Figure 4A). Notably, in IKKα, the aminoindazole ring formed two HBD/HBA interactions with Thr23 and Glu148 in the occluded wall of the site, whereas in IKKβ, because the G-loop is rotated upwards and there is no equivalent wall, these interactions were absent. Moreover, in IKKβ, there is a noticeable shift of the whole scaffold towards the solvent-exposed regions (Figure 4B) to enable the aminoindazole to form an H-bond with Asp103, which presumably compensates for the absence of any interaction with the G-loop wall and increases activity to a level comparable with IKKα. In both isoforms, the 2-phenyl moiety protruded away from the hinge towards the exposed solvent area.

To shift the selectivity profile towards IKKα, we selected **4** for structural optimization based on the notion that the sigma-bond rotation between the phenyl ring and the pyrrolo[2,3-*b*]pyridine would build the flexibility required to engage the three-dimensional array of residues nearby to exploit differences between the two isoforms. Furthermore, this would enable the inclusion of moieties that could also project into solvent and address solubility considerations. Small groups (NH_2_, OH, OMe, OEt, and F) were initially introduced into the phenyl ring to evaluate their impact on the selectivity profile against both IKKs (**5a**–**g**, Table 2). Our goal was to achieve a 1:50 selectivity ratio of IKKα to IKKβ, with a minimum threshold IKKβ inhibitory *K_i_* of >500 nM selected to reduce the likelihood of perturbing canonical NF-kB signalling in cells.

**Figure 5:**
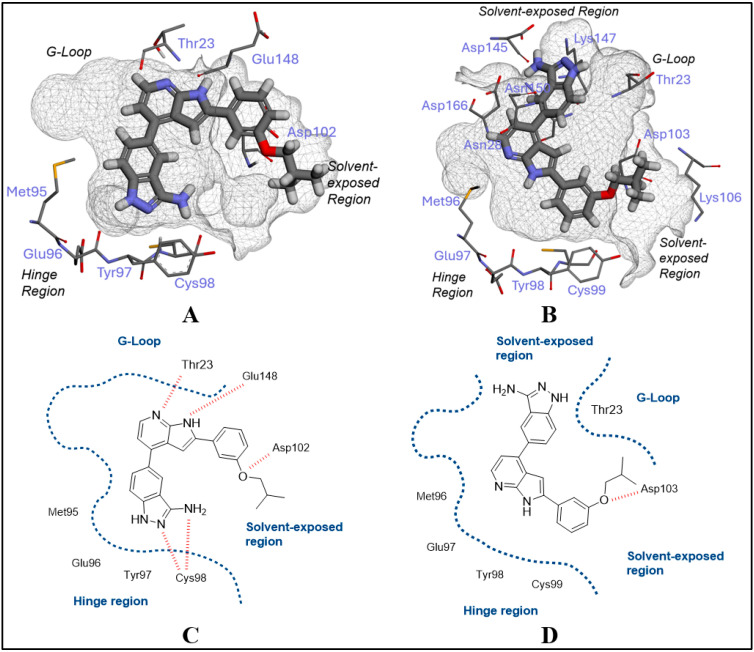
The proposed binding orientations of **5o** to explain its selectivity for IKKα over IKKβ (*K_i_* 19 and 458 nM, respectively). The wire mesh represents the solvent-accessible surface in the ATP-binding state. H atoms: white; C atoms: grey; N atoms: blue; O atoms: red; S atoms yellow. (**A**) A flipped binding orientation was observed compared to that of **4** in IKKα. The aminoindazole binding motif was directed to the hinge region and showed two interactions with Cys98 (1.89 and 2.43 Å). The pyrrolo[2,3-*b*]pyridine ring displayed two interactions with Thr23 and Glu148 (2.05 and 2.25 Å) in the occluded wall opposite. The pendant HBA-containing substituent interacted via H-bonding Asp102 (3.31 Å) in the solvent-exposed area; (**B**) **5o** did not display any poses that formed conventional H-bonds to the hinge residues via either the aminoindazole or the pyrrolo[2,3-*b*]pyridine moiety in IKKβ. Instead, a new orientation was generated that displayed a hook-like pose around the Thr23 G-loop bulge, unique to IKKβ. Here, the aminoindazole is directed into the solvent on one side of the Thr23 protrusion, and the bulky pendant substituent orientated into solvent on the other side of the bulge to form one H-bond between the ether and Asp103 (3.34 Å). (**C**) A 2D diagram of **5o** in the IKKα active site showing the occluded wall in the G-loop region that affords additional interactions with the pyrrolo[2,3-*b*]pyridine binding motif. H-bonds to key residues are shown in red; (**D**) 2D diagram of **5o** in the IKKβ active site to illustrate the hook-like pose around the Thr23 bulge that separates the two solvent-exposed regions. The absence of any H-bonding to the hinge residues in this pose could explain the poor affinity of compounds with a bulky hydrophobic substituent for IKKβ.

This minimal change in the structure did not improve the selectivity profile, which was supported by docking studies. **5a**–**g** all adopted similar binding poses to **4**, with no discriminatory interactions between these substituents and the key amino acid residues lining the solvent-exposed area. Crucially, however, they did not compromise activity and offered handles for further derivatisation with substituents containing appropriately positioned HBDs and HBAs that could exploit differences between each isoform (**5h**–**o**; Table 2). With this set, a noticeable difference in activity between the two isoforms emerged, which appeared to be related to substituent size. Docking studies suggested that, in IKKα, when the steric bulk of substituents appended to the phenyl ring was increased, the AIPP core scaffold adopted a new pose, which was essentially a 180° flip from that of **4** and exemplified by **5o** (Figure 5A). Here, the aminoindazole is now in the hinge region, forming two H-bonds with GK+1 and penetrating deeper in the binding site (Figure 5A), with the pyrrolo[2,3-*b*]pyridine forming H-bonds with Thr23 and Glu148 in the G-loop wall. The phenyl ring and its pendant substituent point towards the solvent-exposed region and gain an additional HB interaction between the ether handle and Asp102, justifying this inversion (Figure 5A). It appears that bulky substituents appended to the phenyl handle do not permit the adoption of the **4** pose because the opening to the solvent adjacent to the hinge in IKKα is too narrow to accommodate such a large pendant group. Docking of this bulkier series into IKKβ can explain why the activity against this isoform is reduced and the selectivity improved. Significantly, no poses were generated with the aminoindazole or the pyrrolo[2,3-*b*]pyridine H-bonding with GK+1 or GK+3. We attribute this to the Thr23 bulge in IKKβ, preventing the inverted pose exemplified by **5o** in IKKα (Figure 5A) or the pose for **4** (Figure 4B) from being adopted. This protrusion will not allow the phenyl group and its bulky pendant substituent to orientate into the tunnel towards the solvent whilst concomitantly having either heterocycle in the AIPP scaffold engaging with the hinge via the conventional kinase binding HBD/HBA motif. The only pose identified for IKKβ involved a hook-like conformation around the displaced Thr23 protrusion, with the aminoindazole accessing the solvent under the G-loop, the phenyl substituent accessing the solvent from the hinge, but without there being any HBD/HBA interaction with the hinge region itself (Figure 5B), which could explain the drop in potency.

**Figure 6:**
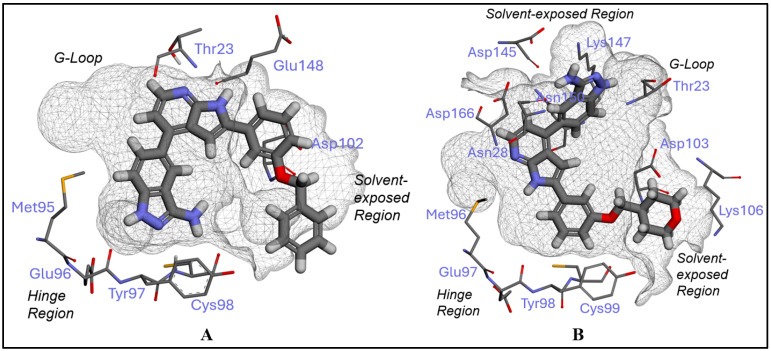
The predicted 3D binding pose for **5r** [**SU1261**] with IKKα (**A**) and **5x** with IKKβ (**B**). The wire mesh represents the solvent-accessible surface in the ATP-binding state. H atoms: white; C atoms: grey; N atoms: blue; O atoms: red; S atoms yellow. (**A**) The aminoindazole binding motif of **5r** [**SU1261**] was situated at the hinge region of IKKα, exhibiting two key interactions with Cys98 (1.99 and 2.56 Å), with the pyrrolo[2,3-b]pyridine positioned at the G-Loop opposite and displaying two key interactions with Thr23 (2.04 Å) and Glu148 (2.39 Å). As with **5o**, the pendant phenyl ring resided in the solvent-exposed region, with the benzyl ether oxygen atom hydrogen bonding to Asp102 (2.52 Å); (**B**) the hook-like binding pose was observed for **5x** in IKKβ, with the aminoindazole moiety making interactions with Thr23 (2.65 Å), Gly24 (3.04 Å), and Asn28 (2.00 Å). Of note were the additional interactions that were observed with the terminal polar pyran group forming an interaction with Lys106 (2.99 Å), which may compensate for an absence of H-bonding with the hinge region in IKKβ.

Encouraged by the observation that compounds with larger pendant substituents on the phenyl ring (**5n,o**) tended to improve selectivity, we next explored whether increasing steric bulk could enhance the window further. We incorporated a range of moieties with varying degrees of saturation, bearing a diverse array of HBAs and HBDs (**5p**–**aa**) to further develop the SAR and the selectivity profile. The general trend observed upon increasing the steric bulk was a more pronounced reduction in IKKβ activity, with the potency against IKKα generally retained. This resulted in a marked increase in selectivity across the series. The docking results for these compounds were consistent with our earlier observations: binding to IKKα inevitably returned the flipped pose, wherein the aminoindazole is bound to the hinge region (**5r** [**SU1261**], Figure 6A) for each of the more sterically hindered analogues. Moreover, increasing the bulk of the phenyl substituents generally prevented any effective binding to the hinge region by the aminoindazole or pyrrolo[2,3-*b*]pyridine moieties in IKKβ, other than the unfavourable hook-like conformation around the displaced Thr23 protrusion seen for **5o**, and no H-bonding to the hinge region. This pose was consistent for those compounds that displayed the lowest activity against IKKβ (**5o**–**r**,**t**,**w**).

A number of analogues did exhibit good IKKβ inhibition despite increased steric hindrance, for example, **5l**,**m**,**x**,**y**. Notably, these derivatives all possess terminal polar groups in their pendant phenyl substituent, and whilst the docking studies consistently returned the hook-like pose in IKKβ described for **5o**,**t**,**p**,**q**,**w** (exemplified by **5o** in Figure 5B), these terminal groups were able to form additional H-bonding interactions with either the protonated Lys106 residue in the case of **5x** (Figure 6B) or the Tyr98 side chain in the case of **5y**, situated in the solvent-exposed region. We propose that these additional interactions could compensate for those not seen with the hinge for **5o**,**t**,**p**,**q**,**w** to explain the improved activity with IKKβ.

Shifting the phenyl pendant substituent to the *para*-position (e.g., **5s**,**u**) tended to improve IKKβ activity to reduce selectivity and was, therefore, not extensively pursued. From a docking perspective, the altered geometry of the ligand caused by the *para* substitution generated a new pose in IKKβ, which could explain this increase in activity. Here, the aminoindazole was positioned along the hinge region to H-bond with GK+1, the pyrrolo[2,3-*b*]pyridine accessing the solvent adjacent to the hinge, and the *para*-pendant substituted phenyl ring projected upwards into the solvent-exposed region below the G-loop to form an additional H-bond with the Thr23 bulge via the ether (**5s**) or sulfonamide (**5u**). Both compounds adopted the flipped pose for IKKα that had been returned across the series.

With a rationale for selectivity established, we next sought to improve the physicochemical properties of the series via the incorporation of solubilising groups and additional heteroatoms in the central phenyl ring (**6a**–**l**, Table 3). **5r** [**SU1261**] was selected as the starting point for the optimisation of the series, owing to its activity and selectivity being recapitulated in cells (*vide infra*). The introduction of polar functional groups and additional nitrogen atoms was explored, with a view to reducing lipophilicity and improving aqueous solubility. Modifications such as linker length extension and heteroatom choice and placement were concurrently explored to see if the potency and specificity of the ligand could be further refined.

**Figure 7:**
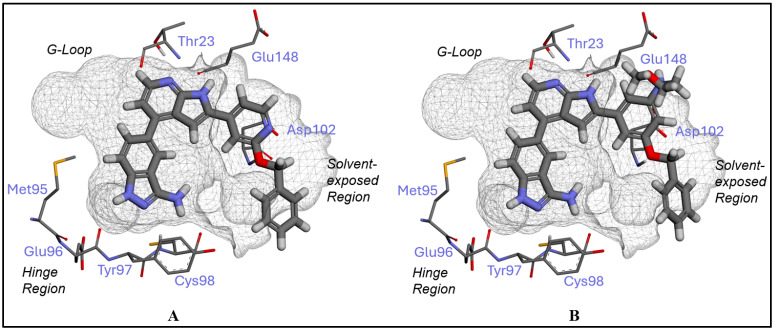
The proposed interactions of **6g** [**SU1349**] (**A**) and **6c** (**B**) with the IKKα active site. The wire mesh represents the solvent-accessible surface in the ATP-binding state. H atoms: white; C atoms: grey; N atoms: blue; O atoms: red; S atoms yellow. (**A**) The aminoindazole motif of **6g** [**SU1349**] was positioned at the hinge region, displaying two H-bond interactions with Cys98 (2.00 and 2.62 Å); the pyrrolo[2,3-*b*]pyridine H-bonded with Thr23 (2.04 Å) and Glu148 (2.46 Å), and the ether showed an HB interaction between the oxygen atom and Asp 102 (2.49 Å); (**B**) the aminoindazole motif of **6c** was positioned at the hinge region, displaying two interactions with Cys98 (1.90 and 2.39 Å), and the pyrrolo[2,3-b]pyridine interacted with the G-loop wall via Thr23 (2.02 Å) and Glu148 (2.26 Å). The pendant phenyl ring was positioned in the solvent-exposed region, making a π–anion interaction with Asp102 (2.52 Å), with the benzyl ether oxygen atom hydrogen bonding to Asp102 (2.75 Å). The additional solubilising alkyl ether substituent was accommodated in the solvent-exposed region.

The addition of heteroatoms and polar moieties when combined with a hydrophobic bulky substituent generally maintained potency for IKKα and decreased IKKβ inhibition, which ultimately improved the selectivity of these analogues. Again, similarities with the prior docking results were observed, with bulky *meta-*substituents on the phenyl ring positioned in the same solvent-exposed orientation in IKKα, allowing significant interaction between the AIPP core with the hinge and G-loop wall in the flipped pose to facilitate potent inhibition (**6g** [**SU1349**], Figure 7A). Furthermore, most analogues had poor activity against IKKβ, which could generally be accounted for *in silico* by the familiar, unfavourable hook-like conformation shown in Figure 5B,D, with the **5o** pose being predominantly replicated in IKKβ with no H-bonding to the hinge.

Compounds with a supplementary solubilising long-chain polar group (**6b,c**) demonstrated similar selectivity profiles that could be explained by our model. The solvent-exposed area in IKKα was large enough for them to consistently adopt the standard flipped pose across the series associated with inhibition (**6c**, Figure 7B). In IKKβ, the increased steric bulk that arises from a disubstituted arrangement in two trajectories could not accommodate the Thr23 bulge under any conditions and generated no viable binding poses.

**Figure 8:**
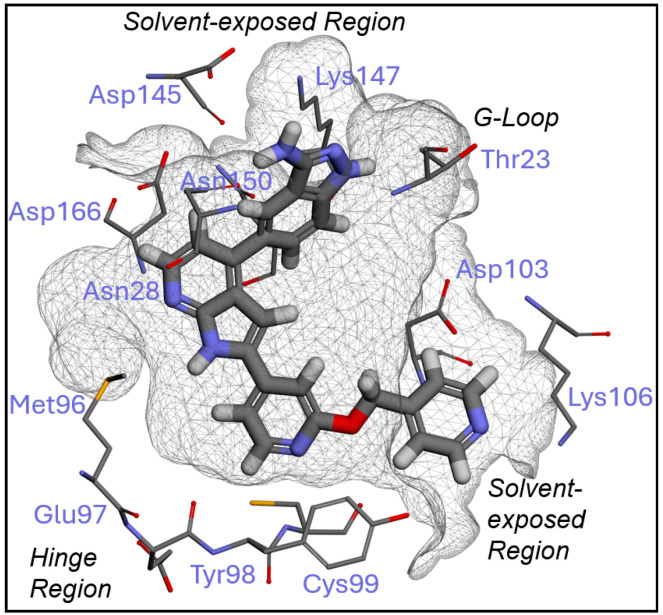
The predicted binding pose of **6k** with IKKβ. The wire mesh represents the solvent-accessible surface in the ATP-binding state. H atoms: white; C atoms: grey; N atoms: blue; O atoms: red; S atoms yellow. The familiar hook-like binding pose was observed, with the aminoindazole moiety H-bonding with Thr23 (2.64 Å) and Asn28 (2.12 Å) but no interaction with the hinge residues. However, an additional interaction was observed between the terminal pyridyl nitrogen and Lys106 (2.44 Å), which could compensate for the absence of any H-bonding with the hinge.

Whilst the addition of heteroatoms and polar moieties generally maintained selectivity for these analogues, there was one exception: **6k** displayed potent inhibition against IKKβ despite possessing a bulky substituent on the phenyl ring. However, the said substituent is a polar pyridyl group, which replicates the poorer selectivity seen for **5l**,**m**,**x**,**y**, all of which contain H-bonding pendant groups. Furthermore, the docked pose of **6k** was similar, with an additional H-bonding interaction seen with Lys 106, which served to improve the affinity of the unfavourable hook-like pose (Figure 8). Together, these data suggest that bulky substituents with a terminal polar functionality that can H-bond to the IKKβ isoform should be avoided if selectivity is to be maintained. Finally, the *para-*substituted analogue **6d** also displayed reduced selectivity and replicated the docking pose seen for **5s**,**u**.

### 2.2. Chemistry

**Scheme 1:**
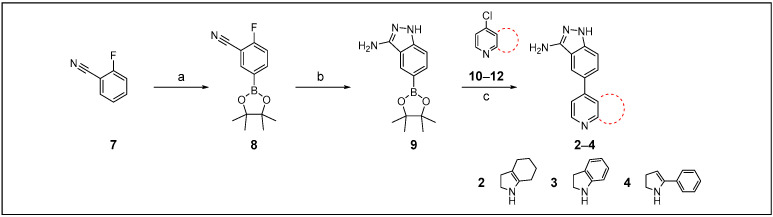
Reagents and conditions. (**a**) B_2_Pin_2_, dtbbpy, [Ir(OMe)(1,5-cod)]_2_, MTBE, 80 °C, 18 h; (**b**) hydrazine hydrate, EtOH, reflux, 30 h; (**c**) PdCl_2_(dtbpf), K_3_PO_4_, EtOH/H_2_O, 120 °C, 20 h.

The synthetic strategy for accessing the compounds described herein began with the organoiridium(I) catalysed C-H activation of commercially available fluorobenzonitrile **7**, giving rise to boronic ester intermediate **8** (Scheme 1) [45]. This was followed by a ring closure using hydrazine, which proceeds via nucleophilic aromatic substitution at the aryl fluoride and nucleophilic attack at the nitrile carbon to afford the key aminoindazole intermediate **9** (Scheme 1). The final step in the preparation of the initial set of AIPP derivatives **2**–**4** was the Suzuki–Miyaura cross-coupling between boronic ester intermediate **9** and the commercially sourced pyrrolo[2,3-*b*]pyridine aryl chlorides **10**–**12** (Scheme 1) [46].

**Scheme 2:**
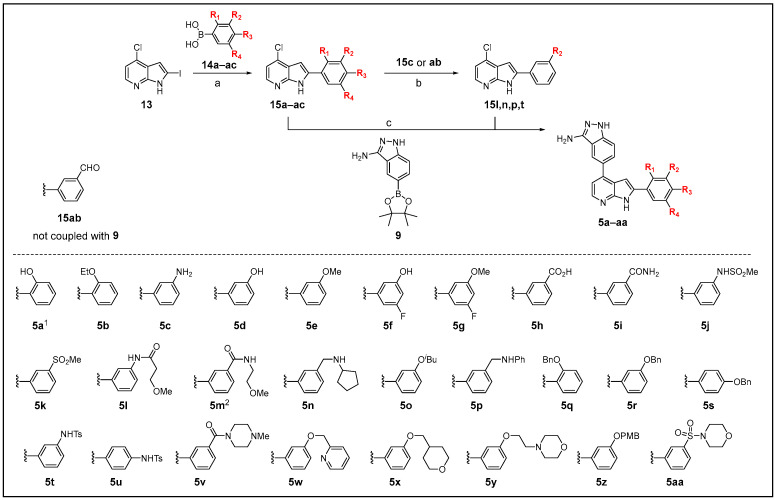
Reagents and conditions. (**a**) K_2_CO_3_, PdCl_2_(PPh_3_)_2_, dioxane/H_2_O, 100 °C, 20 h; (**b**) (**15l**) 3-methoxypropanoic acid, HCTU, Et_3_N, DMF, rt, 18 h, (**15n**) cyclopentylamine, STAB, AcOH, DMA, rt, 48 h, (**15p**) aniline, STAB, AcOH, DMA, rt, 48 h, (**15t**) TsCl, Et_3_N, DCM, 0 °C—rt; (**c**) PdCl_2_(dtbpf), K_3_PO_4_, EtOH/H_2_O, 120 °C, 20 h; ^1^ **5a** was obtained as a byproduct in the preparation of **5q**. ^2^ **5m** was obtained via amide bond formation with **5h** (2-methoxyethylamine, HCTU, Et_3_N, DMF, rt, 18 h).

The preparation of compound **5** series was achieved via the same route as the initial compound set **2**–**4** (Scheme 2). Aryl chlorides **15a**–**ac** were prepared via an additional Suzuki reaction of 4-chloro-2-iodo-pyrrolo[2,3-*b*]pyridine (**13**), with a range of boronic acids **14a**–**ac**, (Scheme 2), with selectivity for the pyrrolo[2,3-*b*]pyridine 2-position achieved through the reduced reactivity of aryl chlorides, compared with aryl iodides within the same scaffold [47]. This enabled two Suzuki coupling reactions to be performed selectively in sequence by the judicious choice of catalyst systems with varying activity i.e., triphenylphosphine palladium catalysis at the iodide moiety, followed by chloride-directing palladium catalysis using a ferrocene-based ligand. Additionally, aniline **15c** was subjected to amide bond formation and tosylation conditions to afford amide and sulfonamide intermediates **15l** and **15t**, respectively. Furthermore, the benzaldehyde functional handle of **15ab** underwent reductive amination, giving rise to amines **15n** and **15p**. As before, the final coupling was carried out between the synthesised aryl halides **15a**–**ac** and boronic ester **9** to afford the final compounds **5a**–**aa**, displayed in Table 3, possessing varying degrees of steric bulk (Scheme 2).

**Scheme 3:**
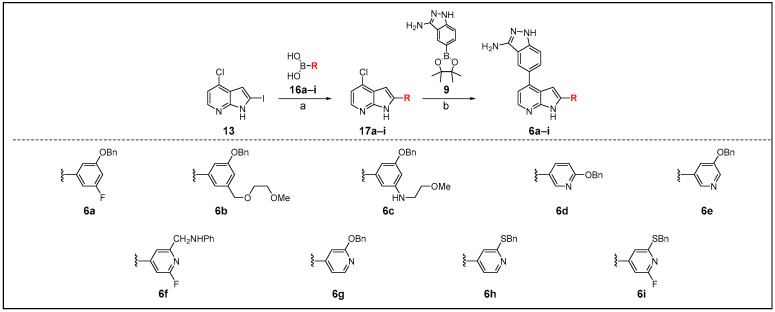
Reagents and conditions. (**a**) K_2_CO_3_, PdCl_2_(PPh_3_)_2_, dioxane/H_2_O, 100 °C, 20 h; (**b**) PdCl_2_(dtbpf), K_3_PO_4_, EtOH/H_2_O, 120 °C, 20 h.

Considering analogues **6a**–**i**, designed with increased solubility in mind, synthesis followed a similar route as those described above. A selection of phenyl and pyridyl boronic acids **16a**–**i** was coupled to the aryl iodide moiety of **13**, giving rise to the intermediates **17a**–**i**. Following this, the scaffold was decorated at the chloride moiety via a Suzuki coupling with the key intermediate **9** to afford compounds **6a**–**i** (Scheme 3).

**Scheme 4:**
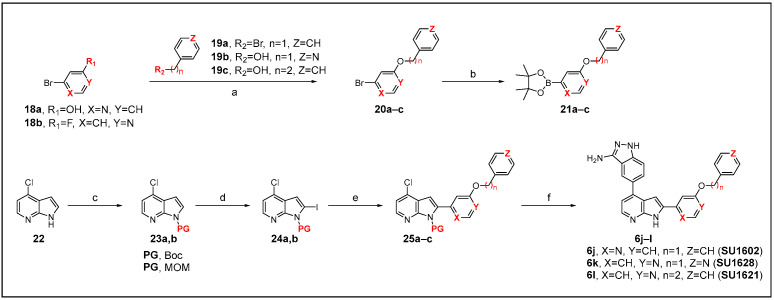
Reagents and conditions. (**a**) (**20a**) Cs_2_CO_3_, DMF, 0 °C—rt, 4 h, (**20b**,**c**) KO*^t^*-Bu, THF, 0 °C—rt, 4 h; (**b**) B_2_Pin_2_, Pd(dppf)Cl_2_, KOAc, dioxane/H_2_O, 110 °C, 18 h. (**c**) (**23a**) K_2_CO_3_, MOMCl, DMF, 0 °C—rt, 18 h, (**23b**) DMAP, Boc_2_O, DMF, rt, 18 h; (**d**) *n*-BuLi, I_2_, THF, −78 °C—rt, 2 h; (**e**) PdCl_2_(dtbpf), Cs_2_CO_3_, dioxane/H_2_O, 80 °C, 18 h; (**f**) compound **9**, PdCl_2_(dtbpf), Cs_2_CO_3_, dioxane/H_2_O, 110 °C, 18 h, (**6j**) HCl/MeOH, reflux, 8 h, (**6k**,**l**) TBAF, THF, reflux, 8 h.

Intermediates in the route towards compounds **6j**–**l** required bespoke synthesis, which began from the commercially available disubstituted pyridines **18a**–**b** (Scheme 4). Intermediates **20a**–**c** were prepared via the S_N_2 halide displacement of benzyl bromide, in the case of **20a**, or nucleophilic aromatic substitution of the aromatic fluoride, in the case of **20b** and **20c**, and they were subsequently subjected to Suzuki–Miyaura borylation conditions to afford the intermediates **21a**–**c** (Scheme 4) [48]. A protecting group (PG) strategy was utilised to aid in purification to afford intermediates **23a**,**b**, employing either methoxymethyl (MOM) chloride or *t*-butyloxycarbonyl (Boc) anhydride, which was then iodinated via lithium–halogen exchange using *n*-BuLi, giving rise to protected intermediates **24a**,**b**. These were then subjected to consecutive Suzuki couplings at the 2- and 4-position halide moieties, sequentially, with boronate esters **21a**–**c** used first to generate intermediates **25a**–**c**, followed by reaction with intermediate **9** to yield the final target compounds **6j**–**l** (Scheme 4).

### 2.3. Physicochemical/DMPK Analysis

**Figure 9:**
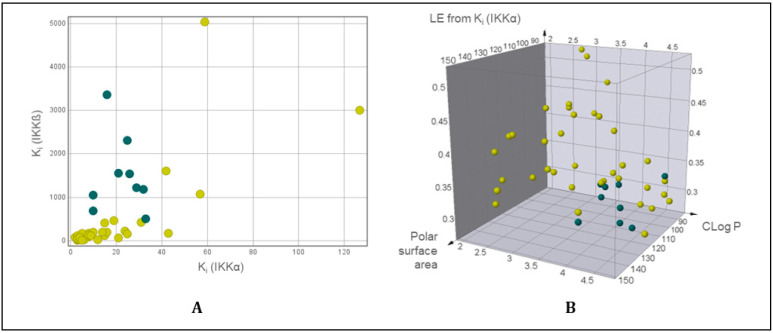
Graphical correlation between the activity and the calculated physicochemical properties. (**A**) IKKα versus IKKβ selectivity analysis. Compounds with superior potency and selectivity profile highlighted in blue; (**B**) physicochemical property analysis. Compounds with superior potency and selectivity profile highlighted in blue.

We next examined our series using DataWarrior (Version 6.0.0), an open-source software package for the generation and analysis of physicochemical attributes [49]. Firstly, compounds were plotted with respect to the inhibition of IKKα versus IKKβ, which included an initial dataset filter, dependent on an IKKα *K_i_* < 40 nM and an IKKβ *K_i_* > 500 nM threshold (Figure 9A).

With this filter applied, the data could be arranged according to the key physicochemical properties: CLogP, topological polar surface area (tPSA), and ligand efficiency (LE) [50], with compounds that possessed adequate potency and selectivity being highlighted in blue (Figure 9B).

**Figure 10:**
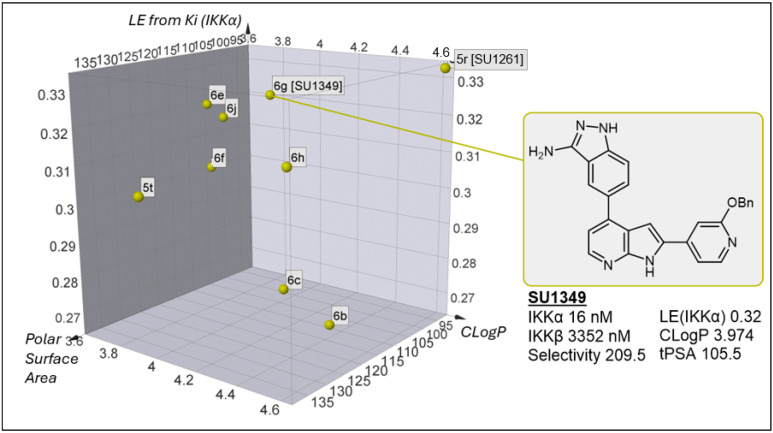
Visual representation of the nine compounds that emerged from the potency and physicochemical property filtering approach. A narrow region of chemical space is occupied concerning tPSA and CLogP. All compounds herein possess a hydrophobic *meta*-substituent with large steric bulk, the majority being a benzyl group connected via either an ether (**5r**, **6b**, **6c**, **6e**, **6g**, **6j**) or thioether (**6h**) linkage.

By removing the compounds with an undesirable selectivity profile, the filtered data points fall into a narrow region of chemical space, possessing a CLogP of ca. 3.5–4.5, a tPSA of 100–120 Å^2^ (with two outliers at 90 and 140 Å^2^), and LE values approximately at the desirable benchmark of 0.3 for further development. All the compounds that met our selectivity and potency criteria possessed bulky hydrophobic *meta*-substituents, with seven of the nine derivatives bearing either a benzyl ether or thioether and a pyridyl group, the most notable example being **6g** [**SU1349**], which boasts a 209-fold selectivity for IKKα over IKKβ (Figure 10).

The modification of **5r** [**SU1261**] to those derivatives in Figure 10, whilst generally improving selectivity, only improved solubility marginally (Table 4), despite the introduction of typically solubilising ether groups (**6b**,**c**), and herein lies the problem to further progress this series: to date, incorporating polar functionality in the solvent-exposed pendant group has improved solubility but significantly compromised the *in vitro* biochemical selectivity for IKKα (*vide supra*). This is clearly demonstrated by comparing **6g** [**SU1349**] with **6k**—the latter has the requisite solubility but is equipotent against both isoforms, whereas the former has the essential selectivity profile but poorer solubility. However, whilst **6g** [**SU1349**] had lower solubility than **6k**, it was the most soluble in the series that displayed selectivity and, furthermore, had low *in vitro* murine clearance (Table 4).

### 2.4. Kinome Profiling

We profiled three compounds from the series across the kinome (Table S1). Our original pan-IKK inhibitor **4** proved to be very promiscuous, inhibiting 44 kinases from a panel of 231 by >80% at 1 μM. The introduction of the pendant benzyloxy substituent to generate **5r** [**SU1261**] markedly reduced off-target kinase inhibition to 10 kinases (>80% at 1 μM), most notably CDK5, CDK9, haspin, and the stress-activated kinases MKK7β and PRAK. Interestingly, exchanging the 2-phenyl pyrrolo[2,3-*b*]pyridine substituent of **5r** [**SU1261**] for the 2-pyridin-4-yl group in **6g** [**SU1349**] not only improved solubility and clearance but also significantly reduced the off-target inhibition of MKK7β and PRAK, although not CDK5 and CDK9.

### 2.5. Cell-Based Assessment

To assess the translation of the biochemical potencies and selectivities described above to a cell-based setting, exemplars from the series were initially examined for inhibitory action against IKKs in the U2OS osteosarcoma cell line. This cell type was utilised to represent cells with a proliferative phenotype dependent on the constitutively activated IKKα-mediated non-canonical NF-κB pathway [51,52]. Compounds **5r** [**SU1261**] (*K_i_* IKKα vs. IKKβ: 10 nM vs. 680 nM) and **6g** [**SU1349**] (*K_i_* IKKα vs. IKKβ: 16 nM vs. 3352 nM) were selected as the most potent compounds with different selectivity ratios and tested in assays involving FCS-stimulated IKKα-mediated p100 phosphorylation (Ser866/870) as a primary pharmacodynamic marker of the non-canonical NF-κB pathway and compared to that of TNFα-stimulated IKKβ-mediated IκBα degradation and p65 phosphorylation (Ser536) as primary pharmacodynamic markers of the canonical NF-κB pathway.

**Figure 11:**
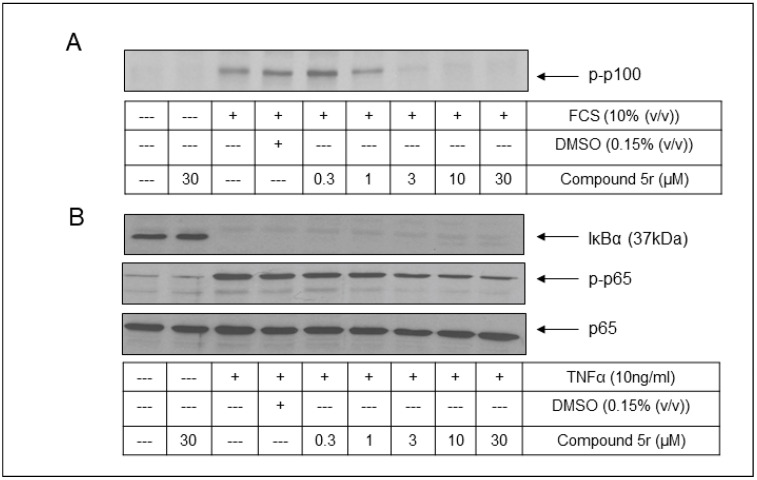
Compound **5r** [**SU1261**] inhibits FCS-stimulated p100 phosphorylation (Ser866/870) with no impact on TNFα-stimulated IkBα degradation and limited impact on phosphorylation of p65 (Ser536) in U2OS osteosarcoma cells. U2OS cells were grown to near confluency and rendered quiescent by serum deprivation for 24 h. In panel (**A**), cells were then exposed to vehicle (DMSO; 0.15% (*v*/*v*)) or increasing concentrations of **5r** [**SU1261**] (0.3–30 µM) for 1 h prior to treatment with FCS (10% (*v*/*v*)) for 4 h, and phospho-p100 (Ser866/870) was assessed by Western blotting. In panel (**B**), cells were exposed to vehicle (DMSO; 0.15% (*v*/*v*)) or increasing concentrations of **5r** [**SU1261**] (0.3–30 µM) for 1 h prior to treatment with TNFα (10 ng/mL) for 30 min and IkBα degradation, phospho-p65 (Ser536), and p65 expression assessed by Western blotting. The results in panels (**A**,**B**) are representative of three independent experiments. Normalised data (*n* = 3) from semi-quantitative scanning densitometry were plotted relative to ‘agonist plus vehicle’ (FCS plus DMSO), and IC_50_ values were established by curve fitting using the Hill equation (see Figure S1A).

**Figure 12:**
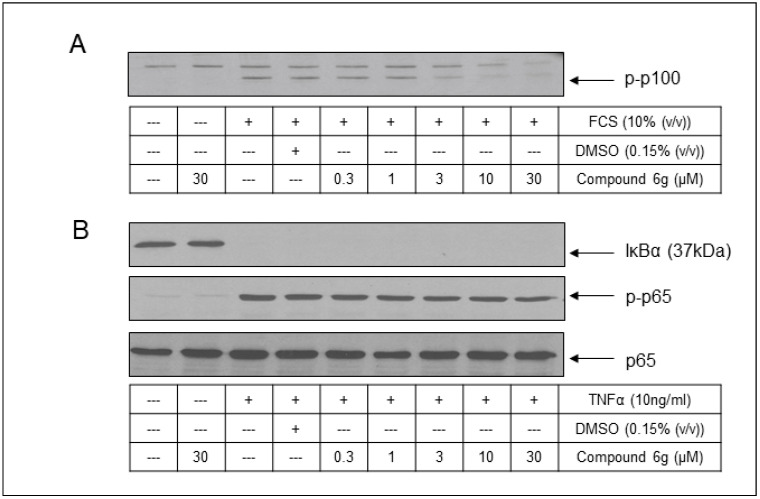
Compound **6g** [**SU1349**] inhibits FCS-stimulated p100 phosphorylation (Ser866/870) but not TNFα-stimulated IkBα degradation nor phosphorylation of p65 (Ser536) in U2OS osteosarcoma cells. U2OS cells were grown to near confluency and rendered quiescent by serum deprivation for 24 h. In panel (**A**), cells were then exposed to vehicle (DMSO; 0.15% (*v*/*v*)) or increasing concentrations of **6g** [**SU1349**] (0.3–30 µM) for 1 h prior to treatment with FCS (10% (*v*/*v*)) for 4 h, and phospho-p100 (Ser866/870) in whole-cell extracts was assessed by Western blotting. In panel (**B**), cells were exposed to vehicle (DMSO; 0.15% (*v*/*v*)) or increasing concentrations of **6g** [**SU1349**] (0.3–30 µM) for 1 h prior to treatment with TNFα (10 ng/mL) for 30 min, and IkBα degradation, phospho-p65 (Ser536), and p65 expression in whole-cell extracts were assessed by Western blotting. The results in panels (**A**,**B**) are representative of three independent experiments. Normalised data (*n* = 3) from semi-quantitative scanning densitometry were plotted relative to ‘agonist plus vehicle’ (FCS plus DMSO), and IC_50_ values were established by curve fitting using the Hill equation (see Figure S1B).

**Figure 13:**
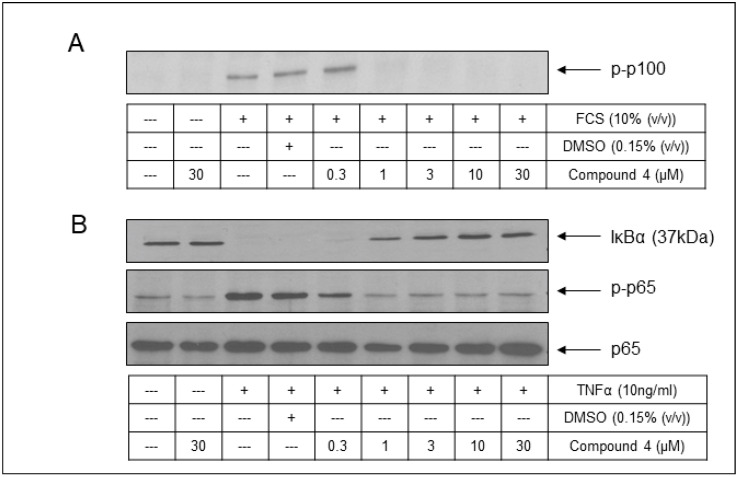
Compound **4** inhibits FCS-stimulated p100 phosphorylation (Ser866/870) and TNFα-stimulated IkBα degradation as well as phosphorylation of p65 (Ser536) in U2OS osteosarcoma cells. U2OS cells were grown to near confluency and rendered quiescent by serum deprivation for 24 h. In panel (**A**), cells were then exposed to vehicle (DMSO; 0.15% (*v*/*v*)) or increasing concentrations of **4** (0.3–30 µM) for 1 h prior to treatment with FCS (10% (*v*/*v*)) for 4 h, and phospho-p100 (Ser866/870) in whole-cell extracts was assessed by Western blotting. In panel (**B**), cells were exposed to vehicle (DMSO; 0.15% (*v*/*v*)) or increasing concentrations of **4** (0.3–30 µM) for 1 h prior to treatment with TNFα (10 ng/mL) for 30 min, and IkBα degradation, phospho-p65 (Ser536), and p65 expression in whole-cell extracts were assessed by Western blotting. The results in panels (**A**,**B**) are representative of three independent experiments. I Normalised data (*n* = 3) from semi-quantitative scanning densitometry were plotted relative to ‘agonist plus vehicle’ (FCS plus DMSO), and IC_50_ values were established by curve fitting using the Hill equation (see Figure S1C).

The pre-treatment of U2OS cells with increasing concentrations of **5r** [**SU1261**] and **6g** [**SU1349**] each resulted in the concentration-dependent inhibition of FCS-stimulated phosphorylation of p100 (Figure 11A and Figure 12A), suggesting the effective inhibition of cellular IKKα, with IC_50_ values of 2.87 μM and 8.75 μM, respectively (Figure S1A,B). For the two IKKβ-dependent readouts selected to assess selectivity over IKKβ (Figure 11B), **5r** [**SU1261**] did not impact TNFα-stimulated IκBα degradation at any concentration examined, whilst at higher concentrations, evidence of an albeit less potent concentration-dependent inhibition of TNFα-stimulated p65 (Ser536) phosphorylation was observed (IC_50_ = 5.64 μM: Figure S1A). For **6g** [**SU1349**], no significant inhibition of TNFα-stimulated IκBα degradation was observed (Figure 12B and Figure S1B), and no significant inhibition was observed against TNFα-stimulated p65 (Ser536) phosphorylation (Figure 12B and Figure S1B; IC_50_ > 30 μM). These data, therefore, confirmed the effective translation of the selectivity profile for the compounds *in vitro* into a cellular environment. Compound **4** was selected as a non-selective IKK inhibitor (*K_i_* IKKα vs. IKKβ: 3 nM vs. 5 nM) to determine whether both NF-κB pathways were affected using our pharmacodynamic readouts. As expected, in a cellular setting, **4** displayed a near-equipotent, concentration-dependent inhibition of FCS-stimulated p100 phosphorylation (Figure 13A and Figure S1C; IC_50_ = 0.69 μM), and TNFα-stimulated IκBα degradation (Figure 13B and Figure S1C; IC_50_ = 0.62 μM) and TNFα-stimulated p65 (Ser536) phosphorylation (Figure 13B and Figure S1C; IC_50_ = 0.3 μM) were observed.

To provide further evidence of cellular activity in an alternative cellular background, the effects upon IKKα-mediated signalling were pursued in the PC-3M prostate cancer cell line. IKKα is now emerging as a relevant target for intervention in prostate cancer, as it supports, firstly, the transcriptional events that underpin the development of key phenotypic hallmarks of cancer [4]. Furthermore, under conditions of androgen deprivation, IKKα has been suggested to support the transition of tumours from the hormone naïve to the castrate-resistant stage of the disease due to the contribution of an inflammatory cytokine-driven switch from IKKβ-mediated canonical NF-κB signalling to that of IKKα-mediated non-canonical NF-κB signalling [32]. A key driver in this setting is B-lymphocyte-derived LTα_1_β_2_ [32], a member of the TNF-superfamily of cytokines and a strong activator of IKKα-mediated non-canonical NF-κB signalling [53].

**Figure 14:**
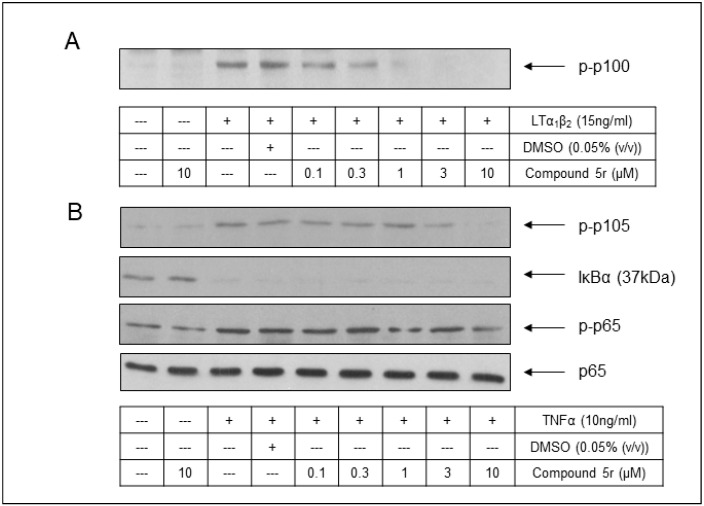
Compound **5r** [**SU1261**] inhibits LTα_1_β_2_-stimulated p100 phosphorylation (Ser866/870) but not TNFα-stimulated IkBα degradation, phosphorylation of p65 (Ser536), nor phosphorylation of p105 (Ser932) in PC-3M prostate cancer cells. PC-3M cells were grown to near confluency and rendered quiescent by serum deprivation for 24 h. In panel (**A**), cells were then exposed to vehicle (DMSO; 0.05% (*v*/*v*)) or increasing concentrations of **5r** [**SU1261**] (0.1–10 µM) for 1 h prior to treatment with LTα_1_β_2_ (15 ng/mL) for 4 h, and phospho-p100 (Ser866/870) in whole-cell extracts was assessed by Western blotting. In panel (**B**), cells were exposed to vehicle (DMSO; 0.05% (*v*/*v*)) or increasing concentrations of **5r** [**SU1261**] (0.1–10 µM) for 1 h prior to treatment with TNFα (10 ng/mL) for 30 min, and IkBα degradation, phospho-p65 (Ser536), phospho-p105 (Ser932), and p65 expression in whole-cell extracts were assessed by Western blotting. The results in panels (**A**,**B**) are representative of three independent experiments. Normalised data (*n* = 3) from semi-quantitative scanning densitometry were plotted relative to ‘agonist plus vehicle’ (LTα_1_β_2_ plus DMSO), and IC_50_ values were established by curve fitting using the Hill equation (see Figure S1D).

The pre-treatment of PC-3M cells with an increasing concentration of **5r** [**SU1261**] resulted in the concentration-dependent inhibition of LTα_1_β_2_-stimulated phosphorylation of p100 (Figure 14A) with an IC_50_ value of 0.57 μM (Figure S1D), suggesting effective inhibition of cellular IKKα. For IKKβ-dependent readouts, (Figure 14B) **5r** [**SU1261**] did not impact TNFα-stimulated IκBα degradation or TNFα-stimulated p65 (Ser536). An additional marker of cellular IKKβ signalling, TNFα-stimulated p105 (Ser932) phosphorylation [54] was also examined and, again, no significant concentration-dependent inhibition was observed (Figure 14B).

**Figure 15:**
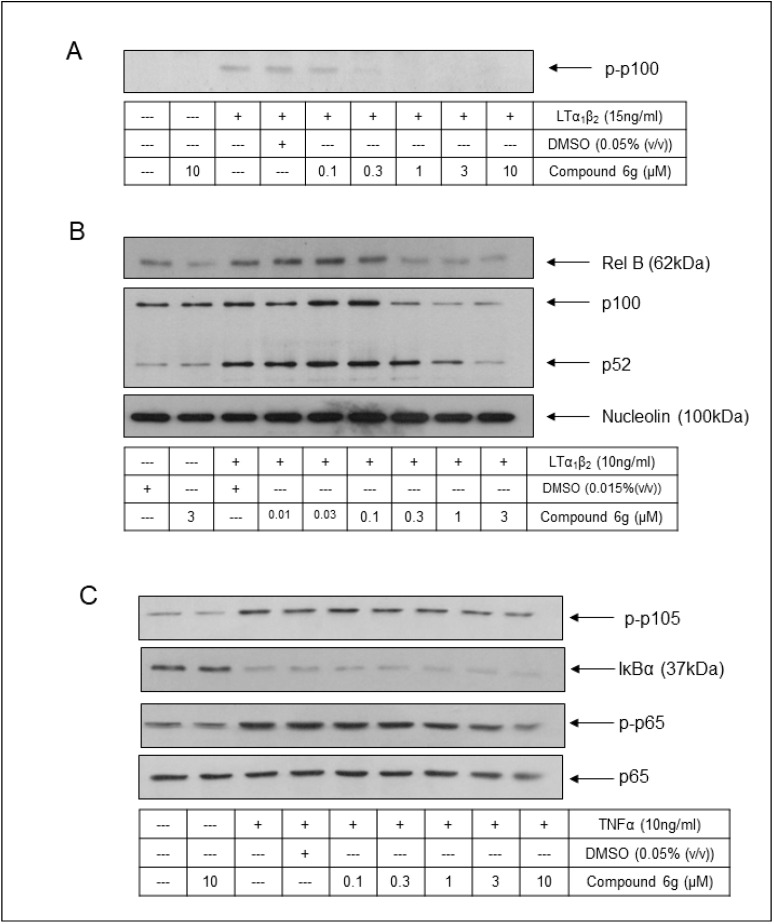
Compound **6g** [**SU1349**] inhibits LTα_1_β_2_-stimulated p100 phosphorylation (Ser866/870) and p52/Rel B nuclear translocation but not TNFα-stimulated IkBα degradation, phosphorylation of p65 (Ser536), nor phosphorylation of p105 (Ser932) in PC-3M prostate cancer cells. PC-3M cells were grown to near confluency and rendered quiescent by serum deprivation for 24 h. In panel (**A**), cells were then exposed to vehicle (DMSO; 0.05% (*v*/*v*)) or increasing concentrations of **6g** [**SU1349**] (0.01–3 µM) for 1 h prior to treatment with LTα_1_β_2_ (15 ng/mL) for 4 h, and phospho-p100 (Ser866/870) in whole-cell extracts was assessed by Western blotting. In panel (**B**), cells were exposed to vehicle (DMSO; 0.05% (*v*/*v*)) or increasing concentrations of **6g** [**SU1349**] (0.3–30 µM) for 1 h prior to treatment with LTα_1_β_2_ (15 ng/mL) for 4 h, and p52/RelB in crude nuclear extracts was assessed by Western blotting. The results in panels (**A**,**B**) are representative of three independent experiments. Normalised data (*n* = 3) from semi-quantitative scanning densitometry were plotted relative to ‘agonist plus vehicle’ (LTα_1_β_2_ plus DMSO), and an IC_50_ value was established by curve fitting using the Hill equation (see Figure S1E). In panel (**C**), cells were exposed to vehicle (DMSO; 0.05% (*v*/*v*)) or increasing concentrations of **6g** [**SU1349**] (0.1–10 µM) for 1 h prior to treatment with TNFα (10 ng/mL) for 30 min, and IkBα degradation, phospho-p65 (Ser536), phospho-p105 (Ser932), and p65 expression in whole-cell extracts was assessed by Western blotting.

Compound **6g** [**SU1349**] also demonstrated effective concentration-dependent inhibition of LTα_1_β_2_-stimulated phosphorylation of p100 (Figure 15A and Figure S1E; IC_50_ = 0.2 μM). Associated with the IKKα-mediated activation of the non-canonical NF-κB pathway is the downstream liberation and the nuclear translocation of p52/RelB-containing NF-κB complexes. The extent of the nuclear translocation of these proteins was also assessed by Western blotting of crude nuclear extracts prepared from PC-3M cells. **6g** [**SU1349**] was observed to inhibit LTα_1_β_2_-stimulated p52 and RelB nuclear translocation in the PC-3M cells, again in a concentration-dependent manner (Figure 15B and Figure S1E; IC_50_ for RelB translocation = 0.15 μM). Related to the markers of TNFα-stimulated canonical NF-κB signalling, **6g** [**SU1349**] had no impact on any of TNFα-stimulated IκBα degradation, phosphorylation of p65 (Ser536), or p105 (Ser932) phosphorylation in these cells (Figure 15C).

Collectively, across the U2OS and PC3M cells, biochemical potency and selectivity were translated to cell-based settings. Whilst potencies against IKKα-related pharmacodynamic markers varied over the two cell lines, this perhaps reflects the extent of the non-canonical NF-κB pathway activation in each setting. Nevertheless, compounds **5r** [**SU1261**] and **6g** [**SU1349**] demonstrated inhibition of agonist-stimulated non-canonical NF-κB pathway activation vs. that of agonist-stimulated canonical NF-κB pathway activation, thus confirming selectivity for IKKα over IKKβ.

### 2.6. Conclusions

Taken together, these data demonstrate that, whilst **4** epitomises an equipotent inhibitor of both cellular IKKα and IKKβ, **5r** [**SU1261**] and **6g** [**SU1349**] represent key exemplars of a novel second-generation series of highly potent IKKα inhibitors that are predicted to interact conventionally by H-bonding with the kinase hinge region.

Regarding selectivity, despite the high sequence homology, a key difference between the two isoforms involves the Thr23-containing G-loop that is opposite the hinge-binding region (Figure 1). In IKKα, the G-loop forms an intact wall to enclose the site on three sides, which offers additional binding sites for a ligand in this isoform. In IKKβ, this sequence is twisted and displaced, thus removing the wall from the opposite side of the hinge-binding region seen in IKKα. The occlusion wall in IKKα offers putative interaction sites that we have exploited to favour binding. Furthermore, the twisting of the G-loop in IKKβ results in Thr23 encroaching into the site itself to create an obstructive bulge that any ligand moiety accessing the solvent area would need to negotiate and explains why bulky pendent phenyl groups can be used to produce discriminatory binding. In IKKα, when the steric bulk of substituents appended to the phenyl ring was increased, the aminoindazole in the hinge region formed two H-bonds with GK+1, with the pyrrolo[2,3-*b*]pyridine forming H-bonds with Thr23 and Glu148 in the G-loop wall opposite. The phenyl ring and its pendant substituent gained an additional HB interaction between the ether handle and Asp102. In IKKβ, no poses were generated with the aminoindazole or the pyrrolo[2,3-*b*]pyridine H-bonding with GK+1 or GK+3. The only pose identified for IKKβ involved a hook-like conformation around the displaced Thr23 protrusion, with the aminoindazole accessing the solvent under the G-loop and the phenyl substituent accessing the solvent from the hinge, but without there being any HBD/HBA interaction with the hinge region itself, which could explain the drop in potency.

This series of compounds demonstrably inhibits agonist-stimulated non-canonical NF-κB signalling. **SU1349** inhibits IKKα-related pharmacodynamic markers of this pathway with IC_50_ values of 0.2 μM (p100 phosphorylation) and 0.15 μM (p52/RelB nuclear translocation) in a prostate cancer cell line with no detectable impact on IKKβ-mediated canonical NF-κB signalling at 10 μM. This represents a significant selectivity index and provides the scientific community with a chemical tool that can be used to gain a better understanding of the key regulatory roles of IKKα in the initiation and development of disease, particularly inflammatory-driven cancers. We have recently determined the in vivo pharmacokinetic parameters following intravenous/intraperitoneal administration for **SU1349** (Table S2). The results from its evaluation in murine models of prostate cancer will be reported imminently by us and our collaborators.

## 3. Materials and Methods

### 3.1. Docking

*In silico molecular docking simulation.* The docking study was carried out using Discovery Studio Client 21.1.0.20298 (Biovia Corp, San Diego, CA, USA) [55]. The X-ray structure of both IKKα and IKKβ kinase enzymes were downloaded from the protein data bank [56] (PDB ID IKKα, 5EBZ [43,57]; IKKβ, 4KIK [42,58]). Non-essential chains and water molecules were removed keeping only one chain containing the kinase domain and its corresponding reference ligand (residue name 5TL and KSA for IKKα and IKKβ, respectively) for each isoform (Chain A for IKKα and Chain B for IKKβ). Chain A was selected for IKKα based on redundancy, owing to the reported structure being a hexamer of identical units, representing a constitutively active mutant (*vide supra*). In the case of IKKβ, Chain B was selected on the basis that in their original communication, Liu et al. [42] state that the activation loop of Chain B is phosphorylated, whereas this is not the case for Chain A. The bonds and bond orders were checked and corrected, as were the terminal residues.

A CHARMm forcefield (chemistry at Harvard macromolecular mechanics) was applied to the protein domain. A Momany–Rone forcefield was selected for the partial charge. The binding site was defined using the coordinates of the reference ligands. The CDOCKER algorithm (CHARMm-based molecular dynamic scheme) [59,60] was used in this study to generate the most stable conformers within the binding sites. Random ligand conformations were generated (10 conformers) from the initial ligand structure through dynamic target temperature (100 K), followed by random rotations. The generated conformers were refined by grid-based (GRID-1) simulated annealing.

The docking protocol was validated by running an initial docking experiment (pre-docking) for the reference ligands of both IKKα and IKKβ (Figure S2). The generated conformers of all proprietary ligands were visualized and analysed to investigate the binding modes within the ATP active sites of both kinases (IKKα and IKKβ).

Evaluation of the resultant poses was performed manually by visual inspection, with the comparison of binding orientation of the ten lowest energy conformations. Emphasis was placed on poses with exceptionally low energy and/or multiple instances of the same pose being adopted. A concomitant comparison with the observed biochemical Ki values was carried out, to propose a rationale for the observed structure–activity relationship.

### 3.2. Chemistry

*General*. Unless otherwise stated, all commercially available reagents and solvents used were obtained from Sigma-Aldrich (Dorset, UK), Fluorochem (Glossop, UK), Fisher Scientific (Loughborough, UK), Acros (Geel, Belgium), Alfa Aesar (Morcambe, UK), Apollo Scientific (Stockport, UK), and Advanced ChemBlocks (Hayward, CA, USA) and used without further purification. Air- or moisture-sensitive reactions were carried out under an argon or nitrogen atmosphere. Microwave reactions were carried out using a Biotage Initiator system. Thin-layer chromatography (TLC) was carried out on aluminium-backed SiO_2_ plates (Merck, Boston, MA, USA, silica gel 60, F_254_) and spots were visualised using ultra-violet light (254 nm) or by staining with potassium permanganate. All tested compounds were determined to be ≥95% purity by LC-MS and analytical HPLC unless otherwise stated. Flash chromatography was performed using a Biotage SP4 automated chromatography system using a silica stationary phase (Fisher Scientific, 60 Å, 35–70 micron; detection wavelength: 254 nm; monitoring: 280 nm), with the specific mobile phases used detailed in the text for individual compounds. Reverse phase HPLC purifications were conducted on Shimadzu Prominance HPLC, (Milton Keynes, UK) using a semi-preparative (50 × 21.2 mm) Luna 5 µm C18 column at 40 °C; flow rate: 6 mL/min; detection wavelength: 254 nm eluting with an acetonitrile/water gradient with 0.1% TFA. NMR spectra were recorded on either a Bruker Avance3/DPX400 (400 MHz), Bruker DRX500 (500 MHz), Bruker AV400 (400 MHz), Bruker AV500HD (500 MHz), or Bruker AV600 (600 MHz) instrument (Coventry, UK) and analysed using Advanced Chemistry Development Labs (ACD/labs) NMR processor 12.00 or MestReNova 10.0 software. Chemical shifts (δ) are recorded in parts per million (ppm) relative to an internal solvent reference (tetramethylsilane) and coupling constants (*J*) in Hertz (Hz). Splitting patterns were indicated as singlet (s), broad singlet (br s), doublet (d), doublet of doublet (dd), triplet (t), quartet (q), and multiplet (m). LCMS was carried out on an Agilent Technologies 1220 series LC system with Agilent 6100 series quadrupole mass spectrometer in ESI/APCI mode (Cheadle, UK). Separation was achieved with an Agilent Eclipse C18 4.6 × 50 mm column; flow rate: 1 mL/min; detection: 254 nm; sample volume: 10 µL; mobile phase: acetonitrile/5 mM ammonium acetate: water/5 mM ammonium acetate; 5%, 1.48 min; 5–100%, 8 min; 100%, 13.5 min; 100–5%, 16.5 min; 18 min. HRMS was carried out on an Exactive (Thermo scientific, Waltham, MA, USA) or LTQ orbitrap (Thermo scientific, Waltham, MA). Synthesis and characterisation of compounds can be found in the Supplementary Materials.

### 3.3. Assessment of Kinase Activity of IKKα and IKKβ In Vitro

IKKα and IKKβ inhibitory activity were determined using a dissociation-enhanced ligand fluorescent immunoassay (DELFIA) based on the protocol of HTScan™ IKKβ Kinase Assay (Cell Signaling Technology, Danvers, MA, USA) as described in [24].

### 3.4. In Vitro DMPK Studies

Pharmacokinetic studies were contracted to Sygnature Discovery Limited. Turbidimetric Solubility was determined by diluting compound solutions (1 μM, 3 μM, 10 μM, 30 μM, 100 μM) prepared in DMSO into 0.01 M phosphate-buffered saline pH7.4. Turbidimetry was used as the endpoint by measuring absorbance at 620 nm.

Intrinsic clearance and half-life were determined using cryopreserved pooled mouse CD1 hepatocytes. Test compounds were analysed at 1 µM in Williams Media E buffer (0.01% DMSO) at a cell density of 0.5 × 10^6^ cells mL^–1^. Cells were incubated at 37 °C for one hour with shaking, and compound depletion was measured by LC-MS/MS at six time points (0.25, 5, 10, 20, 40, and 60 min).

### 3.5. Cell-Based Studies

#### 3.5.1. Materials

All reagents used in cell-based studies were purchased from Sigma (Poole, UK), unless otherwise stated. The antibody-detecting components of the NF-kB pathways used in pharmacodynamic assays were purchased from Cell Signaling Technology (CST) Europe: p-p100 (Ser866/870), p100/p52, IkBα, p-65, p-p65 NF-κB (Ser536), and nucleolin. HRP-conjugated secondary antibodies were sourced from Jackson ImmunoResearch Europe Ltd. (Cambridgeshire, UK).

#### 3.5.2. Methodology

##### Cell Culture

U2OS cells were cultured in McCoy’s 5A Modified Medium, and PC-3M-Luc-C6 prostate cancer cells were cultured in Minimum Essential Medium (MEM), each containing 10% (*v*/*v*) Foetal Calf Serum (FCS), L-glutamine (27 mg/mL), and penicillin/streptomycin (250 units/mL; 100 μg/mL) and incubated in a humidified atmosphere at 37 °C with 5% (*v*/*v*) CO_2_. Cells were grown to near confluence and prior to experimentation rendered quiescent by serum deprivation for 24 h.

##### Treatment of Cells, Preparation of Samples, and Western Blotting

For examination of potential effects of compound on non-canonical NF-κB signalling, cells were exposed to vehicle (DMSO; 0.03–0.05% (*v*/*v*)) or increasing concentrations of compounds (0.01–30 µM) for 1 h prior to treatment with FCS (10% (*v*/*v*); for U2OS cells) or LTα_1_β_2_ (20 ng/mL; for PC-3M cells) for 4 h. The status of p-p100 (Ser 866/870) in whole-cell extracts prepared from 12-well plates was assessed by Western blotting, as described in McKenzie et al. [61]. To examine the effects of compounds on the nuclear translocation of NF-κB complexes, cells were incubated with vehicle (0.015–0.15% (*v*/*v*) DMSO) or increasing concentrations of compounds (0.01–30 μM) as indicated for 1 h prior to exposure to lymphotoxin-β (LTα_1_β_2_) for 4 h and crude nuclear extracts prepared as described in Liu et al. [62]. Thereafter, p52 and RelB status in crude nuclear extracts normalised for protein amounts were assessed by Western blotting.

To examine the potential impact of compounds on canonical NF-κB signalling, cells were then exposed to vehicle (DMSO; 0.05% (*v*/*v*)) or increasing concentrations of compounds (0.01–30 µM) for 1 h prior to treatment with TNF⍺ (20 ng/mL) for 30 min. Whole-cell extracts were prepared, and the status of IκB⍺, phospho-p65 (Ser536), p65, and phospho-p105 (Ser933) was also assessed by Western blotting.

##### Data Analysis

Each figure/panel of Western blotting data represents one of at least three separate experiments. Western blots were scanned and imaged using Adobe Photoshop 5.0.2 software and polypeptide bands were semi-quantified by scanning densitometry using the Scion Image program. Data were normalised to fold expression/inhibition relative to ‘agonist plus vehicle’ treatment and expressed as mean ± s.e.m. IC_50_ values were established by curve fitting using the Hill equation.

## Figures and Tables

**Table 1:** The *K*_i_ values (nM) for the preliminary test set (**2**–**4**) against IKKα and IKKβ.

Compound	*K*_i_ Values (nM)	
	IKKα	IKKβ
**2**	2	77
**3**	3	5
**4**	3	15

**Table 2:** *K_i_* values for series 1 compounds (**5a**–**aa**) against IKKα and IKKβ.

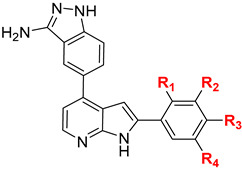						
Cmpd	R1	R2	R3	R4	*K*_i_ Values (nM)	
					IKKα	IKKβ
**5a**	OH	H	H	H	4	42
**5b**	OEt	H	H	H	8	86
**5c**	H	NH_2_	H	H	5	28
**5d**	H	OH	H	H	5	26
**5e**	H	OMe	H	H	5	40
**5f**	H	OH	H	F	4	42
**5g**	H	OMe	H	F	3	32
**5h**	H	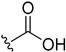	H	H	8	168
**5i**	H	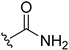	H	H	5	159
**5j**	H	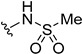	H	H	3	109
**5k**	H	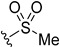	H	H	3	36
**5l**	H	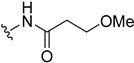	H	H	6	25
**5m**	H	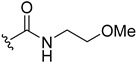	H	H	15	107
**5n**	H	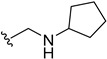	H	H	10	191
**5o**	H	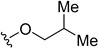	H	H	19	458
**5p**	H	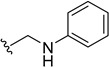	H	H	35	470
**5q**	OBn	H	H	H	57	1064
**5r [SU1261]**	H	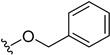	H	H	10	680
**5s**	H	H	OBn	H	16	189
**5t**	H	NHTs	H	H	10	1048
**5u**	H	H	NHTs	H	4	67
**5v**	H	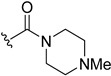	H	H	24	215
**5w**	H	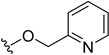	H	H	16	402
**5x**	H	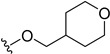	H	H	8	122
**5y**	H	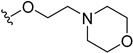	H	H	9	29
**5z**	H	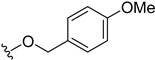	H	H	43	166
**5aa**	H	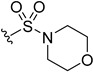	H	H	25	149

**Table 3:** *K_i_* values of series 2 compounds (**6a**–**k**) against IKKα and IKKβ.

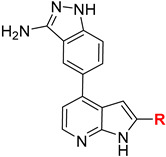			
Cmpd	R	*K*_i_ Values (nM)	
		IKKα	IKKβ
**6a**	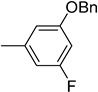	59	5029
**6b**	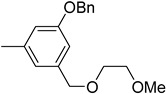	29	1215
**6c**	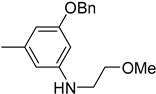	26	1532
**6d**	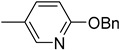	12	22
**6e**	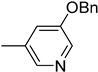	21	1550
**6f**	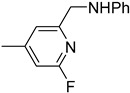	33	503
**6g [SU1349]**	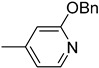	16	3352
**6h**	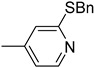	5	1180
**6i**	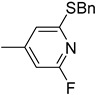	127	3000
**6j**	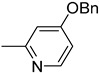	25	2300
**6k**	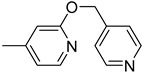	6	12
**6l**	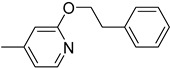	42	1600

**Table 4:** Turbidimetric solubility and murine hepatocyte clearance of selected compounds from the series.

Cmpd	Solubility (μM)	Mouse Hepatocyte Clearance (μL/min/10^6^ Cells)	Mouse Hepatocyte Half-Life (min)
**5r [SU1261]**	<1	38	36
**6b**	3.75	N.D.	N.D.
**6c**	2	N.D.	N.D.
**6e**	6.5	N.D.	N.D.
**6f**	3.75	N.D.	N.D.
**6g [SU1349]**	20	27	51
**6h**	2	N.D.	N.D.
**6j**	2	N.D.	N.D.
**6k**	>100	11	121

N.D. = not determined.

## Data Availability

The data presented in this study are available on request from the corresponding author due to intellectual property arrangements with Cancer Research Horizons, the commercialization engine for Cancer Research UK.

## Supplementary Materials

The following supporting information can be downloaded at: https://www.mdpi.com/article/10.3390/molecules29153515/s1, Table S1: The % inhibition of compounds 4, 5r [SU1261], and 6g [SU1349] against a panel of kinase enzymes. Table S2: *in vivo* murine PK parameters for 6g [SU1349]; Figure S1. (**A**): Compound **5r** [SU1261] inhibits FCS-stimulated p100 phosphorylation (Ser866/870) with no impact on TNFα-stimulated IkBα degradation and limited impact on phosphorylation of p65 (Ser536) in U2OS osteosarcoma cells. (**B**): Compound **6g** [SU1349] inhibits FCS-stimulated p100 phosphorylation (Ser866/870) but not TNFα-stimulated IkBα degradation nor phosphorylation of p65 (Ser536) in U2OS osteosarcoma cells. (**C**): Compound **4** inhibits FCS-stimulated p100 phosphorylation (Ser866/870) and TNFα-stimulated IkBα degradation as well as phosphorylation of p65 (Ser536) in U2OS osteosarcoma cells. (**D**): Compound **5r** [SU1261] inhibits LTα1β2-stimulated p100 phosphorylation (Ser866/870) but not TNFα-stimulated IkBα degradation, phosphorylation of p65 (Ser536), nor phosphorylation of p105 (Ser932) in PC-3M prostate cancer cells. (**E**): Compound **6g** [SU1349] inhibits LTα1β2-stimulated p100 phosphorylation (Ser866/870) and p52/Rel B nuclear translocation but not TNFα-stimulated IkBα degradation, phosphorylation of p65 (Ser536), nor phosphorylation of p105 (Ser932) in PC-3M prostate cancer cells. Figure S2. Comparison of crystalised ligand poses with redocked poses for 5EBZ (IKKα) and 4KIK (IKKβ). S1.0. Compound Synthesis, Purification and Characterisation. S1.1. ^1^H and ^13^C NMR spectra for the pharmacological tools SU1261 [5r] and SU1349 [6g].
