# Supplementary material for: Design and Synthesis of Novel Aminoindazole-pyrrolo[2,3-b]pyridine Inhibitors of IKKα That Selectively Perturb Cellular Non-Canonical NF-κB Signalling
Source: Molecules. 2024 Jul 26;29(15):3515. doi: 10.3390/molecules29153515 (PMC11314561; doi:10.3390/molecules29153515)
Supplement: Supplementary file 1 [file molecules-29-03515-s001.zip › molecules-3098915-supplementary.pdf]

Table S1. The % inhibition of compounds **4**, **5r** [**SU1261**], and **6g** [**SU1349**] against a panel of kinase enzymes. We profiled three compounds from the series across 231 kinases. For **SU1349**, given its structural similarity with **SU1261**, we selected 98 from the 231 kinases, which included those where **SU1261** showed > 30% inhibition (highlighted in blue)

Tested kinase	% inhibition		
	Compound 4	Compound 5r [SU1261]	Compound 6g [SU1349]
Abl(h)	86	44	15
ACK1(h)	83	41	17
ALK(h)	56	27	5
ALK4(h)	25	8	ND*
AMPKα1(h)	23	-7	-5
AMPKα2(h)	43	13	ND
Arg(h)	96	76	24
ARK5(h)	87	33	3
ASK1(h)	18	23	ND
Aurora-A(h)	88	52	36
Aurora-B(h)	99	92	71
Aurora-C(h)	90	32	19
Axl(h)	55	9	ND
Blk(h)	37	-4	ND
Bmx(h)	55	17	ND
BRK(h)	46	17	8
BrSK1(h)	45	-5	ND
BrSK2(h)	41	9	ND
BTK(h)	11	3	ND
CaMKI(h)	9	-6	1
CaMKIIβ(h)	74	47	12
CaMKIIγ(h)	93	84	46
CaMKIIδ(h)	80	61	37
CaMKIV(h)	4	6	-9
CaMKIδ(h)	-8	9	24
CDK1/cyclinB(h)	101	77	58
CDK2/cyclinA(h)	99	61	37
CDK2/cyclinE(h)	86	37	29
CDK3/cyclinE(h)	94	45	37
CDK5/p25(h)	101	96	92
CDK5/p35(h)	101	96	90
CDK6/cyclinD3(h)	82	17	-13
CDK7/cyclinH/MAT1(h)	85	37	14
CDK9/cyclin T1(h)	98	98	99
CHK1(h)	-2	-2	-9
CHK2(h)	28	13	-5
CK1γ1(h)	20	10	ND
CK1γ2(h)	49	28	ND
CK1γ3(h)	29	19	ND
CK1δ(h)	58	14	5
CK2(h)	-7	-5	2
CK2α2(h)	5	4	ND

Tested kinase	% inhibition		
	Compound 4	Compound 5r [SU1261]	Compound 6g [SU1349]
cKit(h)	51	9	ND
CLK2(h)	97	80	68
CLK3(h)	74	23	ND
c-RAF(h)	29	20	2
CSK(h)	-16	-4	ND
cSRC(h)	42	26	-28
DAPK1(h)	-1	-15	4
DAPK2(h)	2	13	ND
DCAMKL2(h)	33	6	ND
DDR2(h)	16	-17	ND
DMPK(h)	5	-4	ND
DRAK1(h)	67	24	ND
DYRK2(h)	43	14	9
eEF-2K(h)	0	-7	ND
EGFR(h)	13	5	-8
EphA1(h)	87	24	-7
EphA2(h)	14	-9	ND
EphA3(h)	19	0	ND
EphA4(h)	9	2	ND
EphA5(h)	19	8	ND
EphA7(h)	26	-3	ND
EphA8(h)	-2	-7	ND
EphB1(h)	63	14	ND
EphB2(h)	12	-8	ND
EphB3(h)	8	-4	ND
EphB4(h)	53	25	ND
ErbB4(h)	20	14	-5
FAK(h)	35	8	ND
Fer(h)	7	-16	ND
Fes(h)	80	50	-31
FGFR1(h)	84	64	-4
FGFR2(h)	79	39	ND
FGFR3(h)	77	41	0
FGFR4(h)	8	6	ND
Fgr(h)	54	33	ND
Flt1(h)	94	58	6
Flt3(h)	102	46	36
Flt4(h)	97	63	43
Fms(h)	91	58	10
Fyn(h)	64	33	ND
GCK(h)	98	86	71
GRK5(h)	5	3	ND
GRK6(h)	0	-8	ND
GRK7(h)	10	-3	ND
GSK3α(h)	95	74	27
GSK3β(h)	92	57	5
Haspin(h)	101	94	92
Hck(h)	21	16	ND

Tested kinase	% inhibition		
	Compound 4	Compound 5r [SU1261]	Compound 6g [SU1349]
Hck(h) activated	38	22	ND
HIPK1(h)	14	-11	5
HIPK2(h)	57	14	ND
HIPK3(h)	26	6	ND
IGF-1R(h)	64	14	ND
IGF-1R(h), activated	61	32	ND
IR(h)	50	4	-1
IR(h), activated	46	23	ND
IRAK1(h)	89	42	37
IRAK4(h)	25	3	-8
IRR(h)	26	1	ND
Itk(h)	18	20	ND
JAK2(h)	88	73	35
JAK3(h)	76	30	18
JNK1α1(h)	80	12	25
JNK2α2(h)	22	-6	ND
JNK3(h)	99	86	62
KDR(h)	93	42	26
Lck(h)	48	34	-19
Lck(h) activated	44	23	ND
LIMK1(h)	58	21	15
LKB1(h)	15	17	ND
LOK(h)	67	51	ND
Lyn(h)	77	31	ND
MAPK1(h)	46	-4	18
MAPK2(h)	29	-1	ND
MAPKAP-K2(h)	38	-3	1
MAPKAP-K3(h)	12	-1	ND
MARK1(h)	84	28	17
MEK1(h)	8	0	8
MELK(h)	41	13	ND
Mer(h)	88	27	12
Met(h)	61	14	-3
MINK(h)	92	51	41
MKK6(h)	17	2	ND
MKK7β(h)	80	99	11
MLCK(h)	0	-10	ND
MLK1(h)	79	56	ND
Mnk2(h)	67	26	ND
MRCKα(h)	-22	-18	ND
MRCKβ(h)	-10	4	ND
MSK1(h)	-2	-24	-4
MSK2(h)	12	-3	ND
MSSK1(h)	12	13	ND
MST1(h)	33	4	ND
MST2(h)	5	-1	4
MST3(h)	6	-3	ND
mTOR(h)	10	8	0

Tested kinase	% inhibition		
	Compound 4	Compound 5r [SU1261]	Compound 6g [SU1349]
mTOR/FKBP12(h)	3	2	ND
MuSK(h)	48	13	ND
NEK11(h)	4	-6	ND
NEK2(h)	5	4	8
NEK3(h)	0	-14	ND
NEK6(h)	6	3	ND
NEK7(h)	-2	-2	ND
NLK(h)	51	1	ND
p70S6K(h)	33	-15	ND
PAK2(h)	18	1	0
PAK4(h)	42	37	ND
PAK5(h)	16	10	ND
PAK6(h)	32	22	ND
PAR-1Bα(h)	86	29	20
PASK(h)	65	16	ND
PDGFRα(h)	8	-12	ND
PDGFRβ(h)	14	1	-14
PDK1(h)	25	7	18
PEK(h)	10	8	ND
PhKγ2(h)	50	21	ND
Pim-1(h)	23	-1	8
Pim-2(h)	25	5	ND
Pim-3(h)	10	6	ND
PKA(h)	29	-6	ND
PKBα(h)	8	10	4
PKBβ(h)	3	-5	ND
PKBγ(h)	12	0	ND
PKCα(h)	5	23	-1
PKCβI(h)	-2	-6	ND
PKCβII(h)	9	-1	ND
PKCγ(h)	-5	-2	ND
PKCδ(h)	16	6	ND
PKCε(h)	-14	0	ND
PKCζ(h)	-1	4	ND
PKCη(h)	2	2	-1
PKCθ(h)	6	-14	ND
PKCι(h)	3	-10	ND
PKCμ(h)	21	18	-9
PKD2(h)	8	-1	-5
PKG1α(h)	29	30	ND
PKG1β(h)	13	1	ND
Plk1(h)	-5	-1	2
Plk3(h)	-6	-7	ND
PRAK(h)	97	91	44
PRK2(h)	85	56	19
PrKX(h)	-21	-22	ND
PTK5(h)	74	29	ND
Pyk2(h)	63	22	ND

Tested kinase	% inhibition		
	Compound 4	Compound 5r [SU1261]	Compound 6g [SU1349]
Ret(h)	52	41	-3
RIPK2(h)	54	19	ND
ROCK-I(h)	13	28	10
ROCK-II(h)	89	58	25
Ron(h)	53	7	ND
Ros(h)	16	11	ND
Rse(h)	30	10	ND
Rsk1(h)	38	2	-18
Rsk2(h)	28	0	ND
Rsk3(h)	53	27	ND
Rsk4(h)	17	4	ND
SAPK2a(h)	-16	0	ND
SAPK2b(h)	-13	-18	ND
SAPK3(h)	99	49	44
SAPK4(h)	87	18	10
SGK(h)	15	-10	22
SGK2(h)	44	30	ND
SGK3(h)	-8	-27	ND
SIK(h)	56	8	ND
Snk(h)	1	-2	ND
SRPK1(h)	5	7	ND
SRPK2(h)	-5	-7	ND
STK33(h)	38	25	ND
Syk(h)	77	32	25
TAK1(h)	28	6	0
TAO1(h)	73	16	5
TAO2(h)	46	7	ND
TAO3(h)	36	4	ND
TBK1(h)	81	22	2
Tec(h) activated	29	62	ND
TGFBR1(h)	28	21	ND
Tie2 (h)	55	18	ND
TLK2(h)	-8	0	ND
TrkA(h)	71	29	-11
TrkB(h)	65	-37	-34
TSSK1(h)	34	14	ND
TSSK2(h)	-9	-9	ND
Txk(h)	43	27	ND
ULK2(h)	2	-4	ND
ULK3(h)	48	14	ND
VRK2(h)	0	-11	ND
WNK2(h)	29	22	ND
WNK3(h)	20	17	ND
ZAP-70(h)	-8	-27	-22
ZIPK(h)	-7	0	1

* ND, not determined.

Table S2. *in vivo* murine PK parameters for **6g [SU1349].** *In vivo* PK studies were performed by Shanghai ChemPartner Co. Ltd. (Shanghai, 201203 CN) Each route of administration involved three CD1 mice all receiving a single dose, with mean values reported. The IV/IP dosing solution was prepared in 10% DMAC+ 10% Solutol HS15+ 80% (15% of HP-β-CD) in water. BQL: Below quantifiable limit of 1 ng/mL for SU1349 in CD1 mouse plasma. All BQL data was excluded from mean value concentration and graphing. Plasma concentrations were determined on an LCMSMS-010 (API4000, triple quadruple) instrument using Positive Ion, ESI MS conditions, with deaxamethasone as an internal standard. Intravenous time points were taken at 5 min, 15 min, 30 min, 1, 2, 4, 8, and 24 hours and intraperitoneal time points were taken at 15 min, 30 min, 1, 2, 4, 6, 8, and 24 hours.

**Administration route**	**Dose (mg/kg)**	**Mean PK parameters**					
		**Clearance (mL/min/kg)**	**Terminal $t_{1/2}$ (hr)**	**V_{ss} (L/kg)**	**C_{max} (ng/mL)**	**T_{max} (hr)**	**F (%)**
I.V.	10	16.7	4.18	0.39			
I.P.	10		3.02		199	0.44	9.3

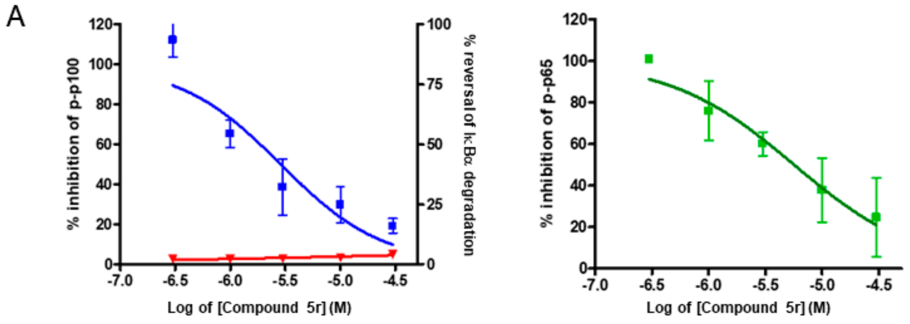

Figure S1A. Compound 5r [SU1261] inhibits FCS-stimulated p100 phosphorylation (Ser866/870) with no impact on TNFα-stimulated IkBα degradation and limited impact on phosphorylation of p65 (Ser536) in U2OS osteosarcoma cells. Left-hand panel: cells were exposed to vehicle (DMSO; 0.15% (v/v)) or increasing concentrations of **5r** [**SU1261**] (0.3-30 μM) for 1 h prior to treatment with FCS (10% (v/v)) for 4 h and phospho-p100 (Ser866/870) was assessed by Western blotting (see Figure 11A). Right-hand panel: cells were exposed to vehicle (DMSO; 0.15% (v/v)) or increasing concentrations of **5r** [**SU1261**] (0.3-30 μM) for 1h prior to treatment with TNFα (10ng/ml) for 30 min and IkBα degradation and phospho-p65 (Ser536) assessed by Western blotting (see Figure 11B. The results are representative of three independent experiments. Normalised data (n=3) from semi-quantitative scanning densitometry was plotted relative to 'agonist plus vehicle' (FCS plus DMSO) and IC_{50} values established by curve fitting using the Hill equation.

B

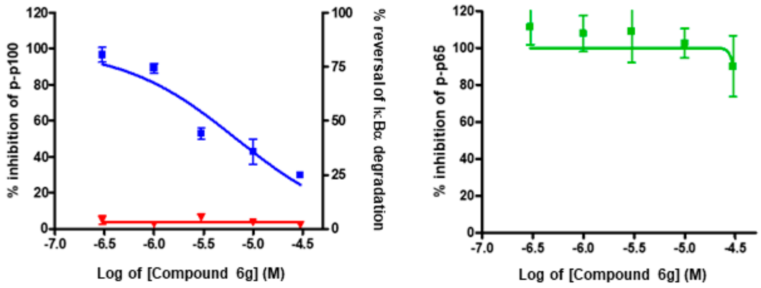

Figure S1B. Compound 6g [**SU1349**] **inhibits FCS-stimulated p100 phosphorylation (Ser866/870) but not TNFα-stimulated IkBα degradation nor phosphorylation of p65 (Ser536) in U2OS osteosarcoma cells.** Left-hand side (blue line): cells were exposed to vehicle (DMSO; 0.15% (v/v)) or increasing concentrations of **6g** [**SU1349**] (0.3-30 μM) for 1h prior to treatment with FCS (10% (v/v)) for 4 h and phospho-p100 (Ser866/870) in whole cell extracts assessed by Western blotting (see Figure 12A). Left-hand side (red line): cells were exposed to vehicle (DMSO; 0.15% (v/v)) or increasing concentrations of **6g** [**SU1349**] (0.3-30 μM) for 1 h prior to treatment with TNFα (10ng/ml) for 30 min and IkBα degradation in whole cell extracts assessed by Western blotting (see Figure 12B) . Right-hand side (green line): cells were exposed to vehicle (DMSO; 0.15% (v/v)) or increasing concentrations of **6g** [**SU1349**] (0.3-30 μM) for 1 h prior to treatment with TNFα (10ng/ml) for 30 min and phospho-p65 (Ser536) in whole cell extracts assessed by Western blotting (see Figure 15B). The results are representative of three independent experiments. Normalised data (n=3) from semi-quantitative scanning densitometry was plotted relative to 'agonist plus vehicle' (FCS plus DMSO) and IC_{50} values established by curve fitting using the Hill equation.

C

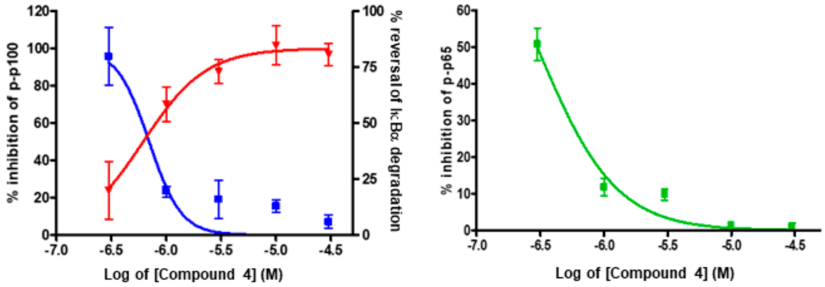

Figure S1C. Compound 4 inhibits FCS-stimulated p100 phosphorylation (Ser866/870) and TNFα-stimulated IkBα degradation as well as phosphorylation of p65 (Ser536) in U2OS osteosarcoma cells Left-hand panel (blue line): cells were exposed to vehicle (DMSO; 0.15% (v/v)) or increasing concentrations of **4** (0.3-30 μM) for 1 h prior to treatment with FCS (10% (v/v)) for 4 h and phospho-p100 (Ser866/870) in whole cell extracts was assessed by Western blotting (see Figure 13A). Left-hand side (red line): cells were exposed to vehicle (DMSO; 0.15% (v/v)) or increasing concentrations of **4** (0.3-30 μM) for 1h prior to treatment with TNFα (10ng/ml) for 30 min and IkBα degradation in whole cell extracts assessed by Western blotting (see Figure 13B). Right-hand side: cells were exposed to vehicle (DMSO; 0.15% (v/v)) or increasing

concentrations of **4** (0.3-30 μM) for 1h prior to treatment with TNFα (10ng/ml) for 30 min and phospho-p65 (Ser536) in whole cell extracts assessed by Western blotting (see Figure 13B) .The results are representative of three independent experiments. Normalised data (n=3) from semi-quantitative scanning densitometry was plotted relative to 'agonist plus vehicle' (FCS plus DMSO) and IC_{50} values established by curve fitting using the Hill equation.

D

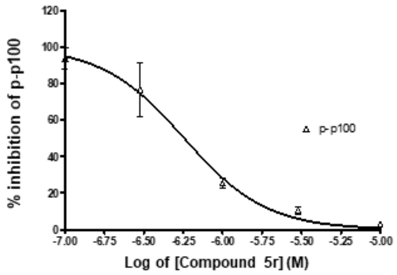

Figure S1D. Compound 5r [SU1261] inhibits $LT\alpha_1\beta_2$-stimulated p100 phosphorylation (Ser866/870) but not TNFα-stimulated IkBα degradation, phosphorylation of p65 (Ser536) nor phosphorylation of p105 (Ser932) in PC-3M prostate cancer cells. Cells were then exposed to vehicle (DMSO; 0.05% (v/v)) or increasing concentrations of **5r** [**SU1261**] (0.1-10 μM) for 1 h prior to treatment with $LT\alpha_1\beta_2$ (15ng/ml) for 4 h and phospho-p100 (Ser866/870) in whole cell extracts assessed by Western blotting (see figure 14A). Results are representative of three independent experiments. Normalised data (n=3) from semi-quantitative scanning densitometry was plotted relative to 'agonist plus vehicle' ($LT\alpha_1\beta_2$ plus DMSO) and IC_{50} values established by curve fitting using the Hill equation.

E

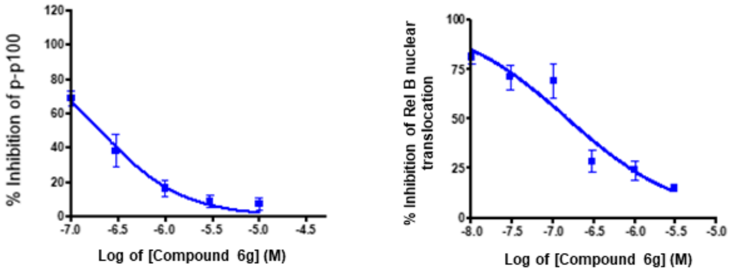

Figure S1E. Compound 6g [SU1349] inhibits $LT\alpha_1\beta_2$-stimulated p100 phosphorylation (Ser866/870) and p52/Rel B nuclear translocation but not TNFα-stimulated IkBα degradation, phosphorylation of p65 (Ser536) nor phosphorylation of p105 (Ser932) in PC-3M prostate cancer cellsLeft-hand side: cells were exposed to vehicle (DMSO; 0.05% (v/v)) or increasing concentrations of **6g** [**SU1349**] (0.01-3 μM) for 1 h prior to treatment with $LT\alpha_1\beta_2$ (15ng/ml) for 4 h and phospho-p100 (Ser866/870) in whole cell extracts assessed by Western blotting (see Figure 15A). Right-hand side: cells were exposed to vehicle (DMSO; 0.05% (v/v)) or increasing concentrations of **6g** [**SU1349**] (0.3-30 μM) for 1 h prior to treatment with $LT\alpha_1\beta_2$ (15ng/ml) for 4 h and p52/RelB in crude nuclear extracts assessed by Western blotting (see Figure 15B). The results are representative of three independent experiments. Normalised data (n=3) from semi-quantitative scanning densitometry was

plotted relative to 'agonist plus vehicle' ($LT\alpha_1\beta_2$ plus DMSO) and an IC_{50} value established by curve fitting using the Hill equation.

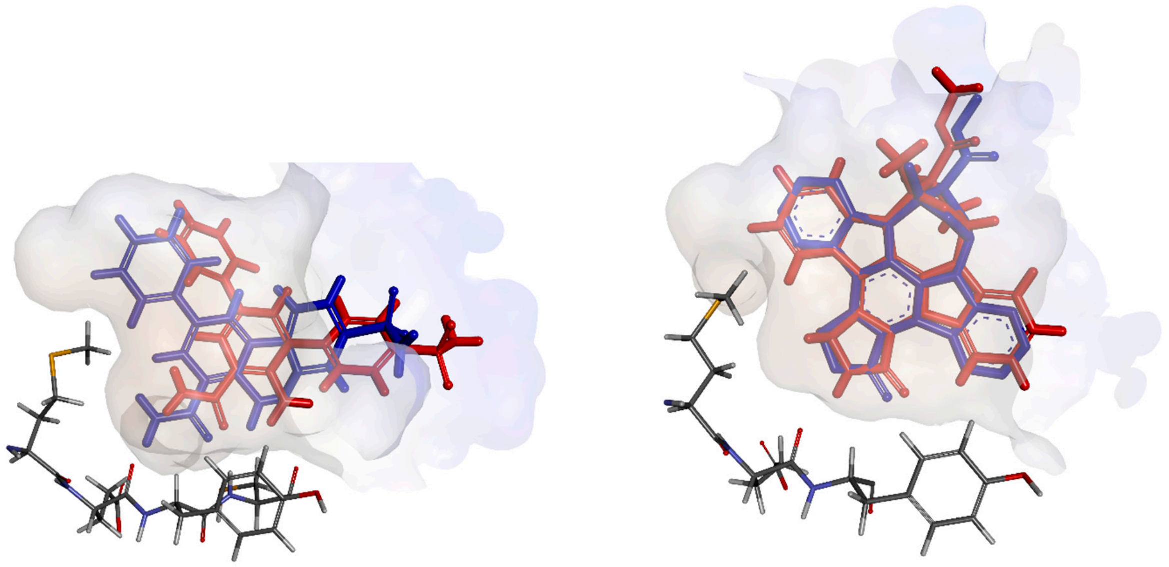

Figure S2. Comparison of IKKα (left, EBZ.pdb) and IKKβ (right, 4KIK.pdb) binding sites, depicting the crystallographic ligand shown in blue, and the re-docked ligand in red. In addition, residues GK–GK+3 are shown for context.

S1.0. Compound Synthesis and Characterisation

Synthesis of 2-fluoro-5-(4,4,5,5-tetramethyl-1,3,2-dioxaborolan-2-yl)benzonitrile (8). A solution of bis(pinacolato)diboron (1.3 g, 5.2 mmol), 4,4′-di-tert-butyl-2,2′-dipyridyl (43 mg, 0.16 mmol) and (1,5-cyclooctadiene)(methoxy)iridium(I) dimer (106 mg, 0.16 mmol) in anhydrous MTBE (10 mL) in a sealed vial (20 mL) was stirred at rt for 1 h. A solution of 2-fluorobenzonitrile (1, 0.6 g, 5 mmol) in anhydrous MTBE (1 mL) was added. The reaction mixture was allowed to stir at 80 °C for 18 h. The reaction mixture was cooled, filtered through celite and evaporated under reduced pressure. The crude residue was used in the next step without further purification.

*Synthesis of 5-(4,4,5,5-tetramethyl-1,3,2-dioxaborolan-2-yl)-1H-indazol-3-amine (**9**).* To a solution of compound **8** (1.98 g, 8 mmol) in EtOH (100 mL), hydrazine hydrate (2.4 mL, 2.47 g, 39 mmol, 50-60%) was added and the reaction was refluxed for 30 h. The solvent was evaporated under reduced pressure. The residue was triturated with a mixture of EtOAc and petroleum ether (1:1, 12 mL), filtered and washed with water and petroleum ether 60-80% to give the titled product **3** as a yellow solid (1.4 g, 67%). ^1H NMR (400 MHz, DMSO-d_6) δ ppm 12.70 (s, 1 H), 8.17 (s, 1 H), 7.49 (d, *J*=8.35 Hz, 1 H), 7.18 (d, *J*=7.91 Hz, 1 H), 5.46 (s, 2 H), 1.30 (s, 12 H). LC-MS: exact mass calculated for $C_{13}H_{18}BN_3O_2$: 259.12, found 260.1 $(M+1)^+$.

*General procedure for synthesis of 5-(substituted pyridin-4-yl)-1H-indazol-3-amine (**2–4**).* To a suspension of 4-chloro-pyridine derivatives (**10–12**, 0.35 mmol), compound **9** (0.136 g, 0.525 mmol) and [1,1′-bis(di-tert-butylphosphino)ferrocene]dichloro palladium(II) catalyst (0.011 g, 0.0175 mmol) in EtOH (1 mL) and water (1 mL), a solution of K_3PO_4 was added (1M, 0.88 mL) and the reaction mixture was heated to 120 °C for 20 h. The reaction mixture was cooled, diluted with EtOAc and washed with water and brine. The organic layer was dried over anhydrous sodium sulfate and concentrated under reduced pressure. The crude residue was purified by column chromatography (10% MeOH in EtOAc) to afford the titled products (**2–4**).

*5-(6,7,8,9-Tetrahydro-5H-pyrido[2,3-b]indol-4-yl)-1H-indazol-3-amine (**2**).* Beige solid (73 mg, 69%). ^1H NMR (400 MHz, DMSO-d_6) δ 11.46 (s, 1H), 11.27 (s, 1H), 8.07 (d, *J* = 4.9 Hz, 1H), 7.78 (s, 1H), 7.33 (d, *J* = 8.6 Hz, 1H), 7.29 (d, *J* = 8.6 Hz, 1H), 6.86 (d, *J* = 4.9 Hz, 1H), 5.43 (s, 2H), 2.71 (t, *J* = 5.8 Hz, 2H), 2.20 (t, *J* = 5.2 Hz, 2H), 1.81 – 1.77 (m, 2H), 1.66 – 1.50 (m, 2H). HRMS (ESI): exact mass calculated for $C_{18}H_{17}N_5$: 303.1552, found 304.1557 $(M+1)^+$.

5-(9H-pyrido[2,3-b]indol-4-yl)-1H-indazol-3-amine (**3**). White powder (36 mg, 35 %). ^{1}H NMR (400 MHz, DMSO-d_6): δ 11.90 (br s, 1H), 11.62 (br s, 1H), 8.44 (d, *J* = 5.0 Hz, 1H), 8.05 (s, 1H), 7.57 (d, *J* = 8.1 Hz, 1H), 7.54 (dd, *J* = 8.7, 1.4 Hz, 1H), 7.51 (d, *J* = 8.1 Hz, 1H), 7.44 (d, *J* = 8.5 Hz, 1H), 7.42-7.38 (m, 1H), 7.11(d, *J* = 5.0 Hz, 1H), 7.02-6.98 (m, 1H), 5.47 (br s, 2H). LC-MS: exact mass calculated for $C_{18}H_{13}N_5$: 299.12, found 300.3 $(M+1)^+$.

5-(2-Phenyl-1H-pyrrolo[2,3-b]pyridin-4-yl)-1H-indazol-3-amine (**4**). Off-white solid (50 mg, 44%). ^{1}H NMR (500 MHz, DMSO-d_6) δ 12.27 (s, 1H), 12.05 (s, 1H), 8.27 – 8.24 (m, 2H), 7.99 (d, *J* = 8.5 Hz, 1H), 7.74 (s, 1H), 7.55 – 7.49 (m, 2H), 7.40 – 7.34 (m, 3H), 7.22 (d, *J* = 8.5 Hz, 1H), 7.17 (d, *J* = 1.9 Hz, 1H), 5.73 (s, 2H).

*General procedure for synthesis of 4-chloro-2-(substituted phenyl)-1H-pyrrolo[2,3-b]pyridine (**15a–ac**).* A suspension of 4-chloro-2-iodo-7-azaindole (0.343 g, 1.23 mmol), substituted phenyl boronic acid (**14a–ac**, 1.52 mmol), K_2CO_3 (0.483 g, 3.49 mmol) and bis(triphenylphosphine) palladium(II) chloride (0.074 g, 0.105 mmol) in dioxane (3 mL) and water (2 mL) was degassed under nitrogen. The reaction mixture was allowed to stir at 100 °C for 20 h. The reaction mixture was cooled to rt and extracted between EtOAc (5 mL) and water (3 mL). The organic layer was washed with brine (2 × 3 mL), dried over anhydrous sodium sulfate and concentrated under reduced pressure. The crude residue was used in the next step without further purification unless otherwise stated below.

4-Chloro-2-(2-ethoxyphenyl)-1H-pyrrolo[2,3-b]pyridine (**15b**). The resulting solid was purified by recrystallization using DCM and hexane to afford the titled compound as an orange solid (207 mg, 62%). ^{1}H NMR (DMSO-d_6): δ 12.13 (br s, 1H), 8.17 (d, *J* = 5.2 Hz, 1H), 7.89 (dd, *J* = 1.6, .6 Hz, 1H), 7.39 – 7.36 (m, 1H), 7.19 (d, *J* = 5.2 Hz, 1H), 7.16 (s, 1H), 7.08 – 7.06 (m, 2H), 4.21 (q, *J* = 6.9 Hz, 2H), 1.46 (t, *J* = 7.0 Hz, 3H). LC-MS: exact mass calculated for $C_{15}H_{13}{}^{35}ClFN_2O$: 273.74, found 273.1 $(M+1)^+$.

3-(4-Chloro-1H-pyrrolo[2,3-b]pyridin-2-yl)-5-fluorophenol (**15f**). The resulting solid was purified by column chromatography (90% EtOAc in petroleum ether) to afford the titled compound as a yellow solid (69 mg, 21.5 %). ^{1}H NMR (DMSO-d_6): δ 12.50 (br s, 1H), 10.13 (s, 1H), 8.19 (d, *J* = 4.8 Hz, 1H), 7.30 (dt, *J* = 1.8, 10.0 Hz, 1H), 7.23 – 7.21 (m, 2H), 6.98 (d, *J* = 2.4 Hz, 1H), 6.59 (dt, *J* = 2.2, 10.4 Hz, 1H). LC-MS: exact mass calculated for $C_{13}H_8{}^{35}ClFN_2O$: 262.03, found 263.13 $(M+1)^+$.

N-(3-(4-Chloro-1H-pyrrolo[2,3-b]pyridin-2-yl)phenyl)methanesulfonamide (**15j**). The crude solid was purified by column chromatography (90% EtOAc in petroleum ether) to afford the titled compound as a pale yellow solid (0.216 g, 64.7%). ^{1}H NMR (DMSO-d_6): δ 12.62 (br s, 1H), 9.88 (br s, 1H), 8.19 (d, *J* = 5.2 Hz, 1H), 7.75 – 7.71 (m, 2H), 7.45 (t, *J* = 16.0 Hz, 1H), 7.22 – 7.20 (m, 2H), 6.88 (d, *J* = 2.0 Hz, 1H), 3.10 (s, 3H). HRMS (ESI): exact mass calculated for $C_{14}H_{13}O_2N_3{}^{35}ClS$: 322.0412, found 322.0410 $(M+1)^+$.

4-Chloro-2-(3-(methylsulfonyl)phenyl)-1H-pyrrolo[2,3-b]pyridine (**15k**). The resulting solid was purified by column chromatography (90% EtOAc in petroleum ether) to afford the titled compound as an off-white solid (245 mg, 62%). ^{1}H NMR (DMSO-d_6): δ 12.73 (br s, 1H), 8.56 (t, *J* = 1.6 Hz, 1H), 8.35 (dt, *J* = 1.2, 8.2 Hz, 1H), 8.24 (d, *J* = 5.3 Hz, 1H), 7.92 (dt, *J* = 1.2, 8.2 Hz, 1H), 7.77 (t, *J* = 7.8 Hz, 1H), 7.26 (dd, *J* = 5.0 Hz, 1H), 7.24 (s, 1H), 3.33 (s, 3H). LC-MS: exact mass calculated for $C_{14}H_{12}{}^{35}ClN_3O_2S$: 321.03, found 322.3 $(M+1)^+$.

4-Chloro-2-(3-isobutoxyphenyl)-1H-pyrrolo[2,3-b]pyridine (**15o**). The resulting solid was purified by recrystallization using DCM and hexane to afford the titled compound as an orange solid (166 mg, 45%). ^{1}H NMR (DMSO-d_6): δ 12.48 (br s, 1H), 8.17 (d, *J* = 5.2 Hz, 1H), 7.58 (s, 1H), 7.56 (s, 1H), 7.37 (t, *J* = 8.0 Hz, 1H), 7.20 (d, *J* = 5.2 Hz, 1H), 7.04 (d, *J* = 2.4 Hz, 1H), 6.94 – 6.92 (m, 1H), 3.85 (d, *J* = 6.4 Hz, 2H), 2.08 – 2.05 (m, 1H), 1.02 (d, J = 6.4 Hz, 6H). LC-MS: exact mass calculated for $C_{17}H_{17}{}^{35}ClN_2O$: 301.79, found 301.1 $(M+1)^+$.

2-(2-(Benzyloxyphenyl)-4-chloro-1H-pyrrolo[2,3-b]pyridine (**15q**). The resulting solid was triturated with hexane and Et_2O to afford the titled compound as a light solid (378 mg, 92%). ^{1}H NMR (DMSO-d_6): δ 12.19 (br s, 1H), 8.18 (d, *J* = 7.5 Hz, 1H), 7.92 (dd, *J* = 2.0, 8.8 Hz, 1H), 7.54 (d, *J* = 7.5 Hz, 1H), 7.42 (t, *J* = 7.0 Hz, 1H), 7.36 – 7.34 (m, 2H), 7.28 (d, J = 8.5 Hz, 1H), 7.17 (d, *J* = 5.0 Hz, 1H), 7.09 (t, *J* = 7.5 Hz, 1H), 7.06 (s, 1H), 5.32 (s, 2H).

3-(4-Chloro-1H-pyrrolo[2,3-b]pyridin-2-yl)phenyl)(4-methylpiperazin-1-yl)methanone (**15v**). The resulting solid was triturated with hexane and filtered through a pad of celite, concentrated under reduced pressure and dried to afford the titled compound as light solid (405 mg, 93%). ^{1}H NMR (DMSO-d_6): δ 12.57 (br s, 1H), 8.19 (d, J = 4.0 Hz, 1H), 8.08 (d, *J* = 6.8 Hz, 1H), 8.00 (s, 1H), 7.55 (t, *J* = 6.2

Hz, 1H), 7.37 (d, *J* = 6.0 Hz, 1H), 7.22 (d, *J* = 4.0 Hz, 1H), 7.11 (s, 1H), 3.66 (br s, 2H), 3.36 (br s, 2H), 2.39 (br s, 2H), 2.23 (br s, 2H), 2.21 (s, 3H).

*4-Chloro-2-(3-(pyridin-2-ylmethoxy)phenyl)-*1H-pyrrolo[2,3-b]pyridine (15w). The crude residue was purified by column chromatography (90% EtOAc in petroleum ether) to give the titled compounds as brown solid (380 mg, 92%). 1H NMR (400 MHz, DMSO-d6) δ 12.52 (s, 1H), 8.60 (d, *J* = 4.6 Hz, 1H), 8.18 (d, *J* = 5.2 Hz, 1H), 7.88 – 7.84 (m, 1H), 7.73 (s, 1H), 7.61 (d, *J* = 7.7 Hz, 1H), 7.58 (d, *J* = 7.8 Hz, 1H), 7.45 – 7.37 (m, 1H), 7.36 (d, *J* = 7.2 Hz, 1H), 7.21 (d, *J* = 5.2 Hz, 1H), 7.06 – 7.03 (m, 2H), 5.30 (s, 2H). LC-MS: exact mass calculated for $C_{19}H_{14}{}^{35}ClN_3O$: 335.08, found 336.1 $(M+1)^+$.

*2-(3-((Tetrahydro-2H-pyran-4-yl)methoxy)phenyl)-4-chloro-1H-pyrrolo[2,3-b]pyridine (**15x**).* The crude residue was triturated with 50% EtOAc in petroleum ether to afford the titled product as brown solid (336 mg, 80%). ^{1}H NMR (400 MHz, DMSO-*d*$_6$) δ 12.51 (s, 1H), 8.17 (d, *J* = 5.2 Hz, 1H), 7.58 – 7.56 (m, 2H), 7.39 – 7.35 (m, 1H), 7.20 (d, *J* = 5.2 Hz, 1H), 7.05 (d, *J* = 2.0 Hz, 1H), 6.94 (d, *J* = 7.2 Hz, 1H), 3.93 (d, *J* = 6.8 Hz, 2H), 3.89 (dd, *J* = 11.2, 2.8 Hz, 2H), 3.37 (dd, *J* = 12.4, 1.6 Hz, 2H), 2.08 – 2.00 (m, 1H), 1.72 (d, *J* = 11.6 Hz, 2H), 1.42 – 1.31 (m, 2H). LC-MS: exact mass calculated for $C_{19}H_{19}{}^{35}ClN_2O_2$: 342.11, found 343.3 $(M+1)^+$.

*4-(2-(3-(4-Chloro-1H-pyrrolo[2,3-b]pyridin-2-yl)phenoxy)ethyl)morpholine (**15y**).* The crude residue was purified by flash chromatography (10% MeOH in EtOAc) to obtain the titled compound as beige solid (136 mg, 31%). ^{1}H NMR (400 MHz, DMSO-*d*$_6$) δ 12.49 (s, 1H), 8.17 (d, *J* = 5.2 Hz, 1H), 7.60 (s, 1H), 7.58 (d, *J* = 8.0 Hz, 1H), 7.39 – 7.35 (m, 1H), 7.20 (d, *J* = 5.2 Hz, 1H), 7.06 (d, *J* = 2.0 Hz, 1H), 6.95 (dd, *J* = 8.1, 2.0 Hz, 1H), 4.19 (t, *J* = 5.8 Hz, 2H), 3.61 – 3.58 (m, 4H), 2.73 (t, *J* = 5.8 Hz, 2H), 2.51-2.48 (m, 4H). LC-MS: exact mass calculated for $C_{19}H_{20}{}^{35}ClN_3O_2$: 357.12, found 358.1 $(M+H)^+$.

*4-Chloro-2-(3-((4-methoxybenzyl)oxy)phenyl)-1H-pyrrolo[2,3-b]pyridine (**15z**).* The resulting solid was purified by column chromatography (90% EtOAc in petroleum ether) and triturated with Et_2O to afford the titled compound as an orange solid (228 mg, 51%). ^{1}H NMR (DMSO-*d*$_6$): δ 12.48 (br s, 1H), 8.17 (d, *J* = 5.2 Hz, 1H), 7.68 (t, *J* = 2.0 Hz, 1H), 7.58 (d, *J* = 8.0 Hz, 1H), 7.43 – 7.41 (m, 3H), 7.21 (d, *J* = 5.2 Hz, 1H), 7.05 (s, 1H), 6.98 – 6.96 (m, 3H), 5.13 (s, 2H), 3.75 (s, 3H). LC-MS: exact mass calculated for $C_{21}H_{17}{}^{35}ClN_2O_2$: 364.10, found 365.7 $(M+1)^+$.*4-((3-(4-Chloro-1H-pyrrolo[2,3-b]pyridin-2-yl)phenyl)sulfonyl)morpholine (**15aa**).* The resulting solid was triturated with boiling MeOH, filtered and dried to afford the titled compound as brown solid (88 mg, 19%). ^{1}H NMR (DMSO-*d*$_6$): δ 12.83 (br s, 1H), 8.37 – 8.35 (m, 1H), 8.23 (d, *J* = 4.8 Hz, 1H), 7.78 – 7.75 (m, 4H), 7.25 (d, *J* = 5.2 Hz, 1H), 7.23 (d, *J* = 2.0 Hz, 1H), 3.66 – 3.65 (m, 4H), 2.96 – 2.94 (m, 4H).

*3-(4-Chloro-7-azaindol-2-yl)benzaldehyde (**15ab**).* The resulting solid was triturated with petroleum ether and EtOAc to afford the titled compound as brown solid (240 mg, 186%). ^{1}H NMR (DMSO-*d*$_6$): δ ppm 7.17 (s, 1H) 7.24 (d, *J* = 5.27 Hz, 1 H) 7.70 – 7.77 (m, 1 H) 7.91 (d, *J* = 7.47 Hz, 1 H) 8.21 (d, *J* = 5.27 Hz, 1 H) 8.33 (d, *J* = 7.47 Hz, 1 H) 8.57 (s, 1 H) 10.09 (s, 1 H). LC-MS: exact mass calculated for $C_{14}H_9{}^{35}ClN_2O$: 257.7, found 257.1 $(M+H)^+$.

*2-(4-(Benzyloxy)phenyl)-4-chloro-1H-pyrrolo[2,3-b]pyridine (**15s**).* The crude residue was purified by column chromatography (90% EtOAc in petroleum ether) followed by trituration to the resulting solid by a mixture of hexane/Et_2O to afford the titled compound as an off-white solid (177 mg, 43%). ^{1}H NMR (DMSO-*d*$_6$): δ 12.38 (br s, 1H), 8.13 (d, *J* = 5.5 Hz, 1H), 7.94 (d, *J* = 8.5 Hz, 1H), 7.47 (d, *J* = 7.0 Hz, 2H), 7.41 (t, *J* = 7.5 Hz, 2H), 7.35 (d, *J* = 7.5 Hz, 1H), 7.18 (d, *J* = 5.0 Hz, 1H), 7.12 (d, *J* = 8.5 Hz, 1H), 6.87 (d, *J* = 2.0 Hz, 1H), 5.19 (s, 2H).

*Synthesis of N-(3-(4-chloro-1H-pyrrolo[2,3-b]pyridin-2-yl)phenyl)-3-methoxypropanamide (**15l**).* A solution of compound **15c** (0.10 g, 0.4 mmol) and 3-methoxypropanoic acid (37 μL, 0.4 mmol) in DMF (5 mL) was allowed to stir at rt for 5 min. HCTU (495 mg, 1.2 mmol) and trimethylamine (168 μL, 1,2 mmol) were added and the solution allowed to stir at rt for 18 h. The reaction mixture was cooled, extracted between EtOAc and water. The organic layer was dried over anhydrous sodium sulfate and removed under reduced pressure. The resulting residue was purified by column chromatography (1% Et_3N and 10% MeOH in EtOAc) to give the titled product as a pale-yellow solid (95 mg, 72 %). ^{1}H NMR (500 MHz, DMSO-*d*$_6$) δ 12.54 (s, 1H), 10.04 (s, 1H), 7.89 – 7.86 (m, 2H), 7.66 (d, *J* = 7.8 Hz, 1H), 7.57 (d, *J* = 7.8 Hz, 1H), 7.47 (t, *J* = 10.0 Hz, 1H), 7.22 (d, *J* = 6.5 Hz, 1H), 6.81 (d, *J* = 1.8 Hz, 1H), 3.65 (t, *J* = 8.0 Hz, 2H), 3.27 (s, 3H), 2.04 (t, *J* = 8.0 Hz, 2H). LC-MS: exact mass calculated for $C_{17}H_{16}{}^{35}ClN_3O_2$: 329.09, found 330.3 $(M+H)^+$.

*Synthesis of N-(3-(4-chloro-1H-pyrrolo[2,3-b]pyridin-2-yl)phenyl)-4-methylbenzenesulfonamide (**15t**).* Tosyl chloride (136 μL, 1 mmol) was added to a solution of compound **15c** (0.244 g, 1.04 mmol) and triethyl amine (0.3 mL, 2 mmol) in anhydrous DCM (5 mL) at 0 °C. The reaction mixture was allowed to stir at rt for 18 h. The reaction mixture was washed with saturated solution of sodium hydrogen carbonate (5 mL) and brine (5 mL), dried over anhydrous sodium sulfate and removed under reduced pressure. The crude residue was purified by column chromatography (90% EtOAc in petroleum ether) to afford the titled compound as a pale-yellow solid (301 mg, 73%). ^{1}H NMR (DMSO-d_6): δ 12.57 (br s, 1H), 10.36 (br s, 1H), 8.19 (d, *J* = 5.2 Hz, 1H), 7.71 (d, *J* = 8.4 Hz, 2H), 7.65 – 7.64 (m, 2H), 7.38 – 7.36 (m, 3H), 7.22 (d, *J* = 5.2 Hz, 1H), 7.08 – 7.05 (m, 1H), 6.74 (d, *J* = 2.0 Hz, 1H), 2.33 (s, 3H).

*General procedure for synthesis of N-(3-(4-chloro-1H-pyrrolo[2,3-b]pyridin-2-yl)benzyl)-N-substituted amine (**15n,p**).* A solution of compound **15ab** (0.12 g, 0.46 mmol), appropriate amine (0.6 mmol), sodium triacetoxyborohydride (0.14 g, 0.69 mmol) and acetic acid (0.036 mL, 0.59 mmol) in dimethylacetamide (2 mL) at rt for 48 h. The reaction mixture was poured into 1M sodium carbonate solution, stirred in an ice bath for 3 h and filtered.

*N-[3-(4-Chloro-7-azaindole)benzyl]-N-cyclopentylamine (**15n**).* The collected solid was purified by column chromatography (10% MeOH in EtOAc) to afford the desired compound as a white solid (102 mg, 68%), ^{1}H NMR (400 MHz, DMSO-d_6) δ 12.49 (br s, 1 H), 8.17 (d, *J*=5.27 Hz, 1 H), 7.97 (s, 1 H), 7.84 (d, *J*=7.47 Hz, 1 H), 7.41 (t, *J*=7.69 Hz, 1 H), 7.35 (d, *J*=7.91 Hz, 1 H), 7.21 (d, *J*= 5.27 Hz, 1 H), 6.99 (s, 1 H), 3.74 (s, 2 H), 3.03 – 3.01 (m, 1 H), 1.73 – 1.71 (m, 2 H), 1.64 – 1.62 (m, 2 H), 1.47 – 1.44 (m, 2 H), 1.37 – 1.35 (m, 2 H). LC-MS: exact mass calculated for $C_{19}H_{20}{}^{35}ClN_3$: 325.13, found 326.2 $(M+1)^+$.

*N-[3-(4-Chloro-7-azaindole)benzyl]-N-phenylamine (**15p**).* The collected solid was purified by column chromatography (60% EtOAc in petroleum ether) to afford the desired compound as a white solid (118 mg, 77%). ^{1}H NMR (400 MHz, DMSO-d_6) δ 12.54 (br s, 1 H), 8.17 (d, *J* = 5.27 Hz, 1 H), 8.04 (s, 1 H), 7.85 (d, *J* = 7.47 Hz, 1 H), 7.43 (t, *J* = 7.69 Hz, 1 H), 7.38 (d, *J* = 7.91 Hz, 1 H), 7.21 (d, *J* = 5.27 Hz, 1 H), 7.08-7.02 (m, 2 H), 6.97(s, 1 H), 6.62 (d, *J* = 7.47 Hz, 2 H), 6.51 (t, *J* = 7.25 Hz, 1 H), 6.27 (t, *J* = 5.93 Hz, 1 H), 4.32 (d, *J* = 6.15 Hz, 2 H). LC-MS: exact mass calculated for $C_{20}H_{16}{}^{35}ClN_3$: 333.10, found 334.1 $(M+1)^+$.

*Synthesis of N-(4-(4-chloro-1H-pyrrolo[2,3-b]pyridin-2-yl)phenyl)-4-methylbenzenesulfonamide (**15u**).* Tosyl chloride (136 μL, 1 mmol) was added to a solution of compound **15ac** (0.244 g, 1 mmol) and triethyl amine (0.3 mL, 2 mmol) in anhydrous DCM (5 mL) at 0 °C. The reaction mixture was allowed to stir at rt for 18 h. The reaction mixture was quenched with saturated solution of sodium hydrogen carbonate (5 mL). The organic layer was washed with brine (5 mL), dried over anhydrous sodium sulfate and removed under reduced pressure. The crude residue was purified by column chromatography (90% EtOAc in petroleum ether) to afford the titled compound as an off-white solid (199 mg, 50%). ^{1}H NMR (DMSO-d_6): δ 12.39 (br s, 1H), 10.47 (br s, 1H), 8.13 (d, *J* = 5.2 Hz, 1H), 7.85 (dd, *J* = 2.0, 6.8 Hz, 2H), 7.69 (dd, *J* = 1.6, 6.4 Hz, 2H), 7.36 (d, *J* = 8.0 Hz, 2H), 7.17 (dd, *J* = 0.8, 6.4 Hz, 3H), 6.87 (d, J = 2.4 Hz, 1H), 2.33 (s, 3H).

*General procedure for synthesis of 5-(2-(substituted phenyl)-1H-pyrrolo[2,3-b]pyridin-4-yl)-1H-indazol-3-amine (**5a–aa**).* K_3PO_4 (1M, 1.2 mL) was added to a suspension of compounds **15** (0.37 mmol), compound **9** (0.146 g, 0.56 mmol) and [1,1′-bis(di-tert-butylphosphino)ferrocene]dichloro palladium(II) catalyst (0.028 g, 0.04 mmol) in EtOH (3 mL). The reaction mixture was heated to 120 °C for 20 h. The reaction mixture was cooled to rt, diluted with EtOAc, washed with water and brine. The organic layer was dried over anhydrous sodium sulfate and concentrated under reduced pressure.

*2-(4-(3-Amino-1H-indazol-5-yl)-1H-pyrrolo[2,3-b]pyridin-2-yl)phenol (**5a**).* Obtained as a byproduct in the preparation of **5q**. The crude residue was purified by column chromatography (80% EtOAc in petroleum ether) to give the titled product as off-white solid (10 mg, 8%). ^{1}H NMR (DMSO-d_6): δ 11.67 (br s, 1H), 11.54 (br s, 1H), 10.17 (br s, 1H), 8.23 (d, *J* = 5.2 Hz, 1H), 8.19 (s, 1H), 7.85 (dd, *J* = 1.6, 7.6 Hz, 1H), 7.69 (dd, *J* = 1.6, 8.4 Hz, 1H), 7.40 (d, *J* = 8.8 Hz, 1H), 7.28 (s, 1H), 7.17 – 7.15 (m, 2H), 6.98 – 6.96 (m, 1H), 6.92 – 6.89 (m, 1H), 5.50 (br s, 2H). HRMS (ESI): exact mass calculated for $C_{20}H_{15}ON_5$: 341.1349, found 342.1346 $(M+1)^+$.

*5-(2-(2-Ethoxyphenyl)-1H-pyrrolo[2,3-b]pyridin-4-yl)-1H-indazol-3-amine (**5b**).* The crude residue was purified by column chromatography (90% EtOAc in petroleum ether) to afford the titled compound as an off-white solid (56 mg, 41%). ^{1}H NMR (DMSO-d_6): δ 11.81 (br s, 1H), 11.54 (br s, 1H), 8.25 (d, *J* = 5.0 Hz, 1H), 8.22 (s, 1H), 7.92 (dd, *J* = 2.0, 8.0 Hz, 1H), 7.72 (dd, *J* = 1.5, 9.0 Hz, 1H), 7.39 – 7.37 (m, 2H), 7.34 – 7.32 (m, 1H), 7.18 (d, *J* = 5.0 Hz, 1H), 7.14 (d, *J* = 6.4 Hz, 1H), 7.05 (t, *J* = 5.8 Hz, 1H), 5.50 (br s, 2H), 4.18

(q, *J* = 5.5 Hz, 2H), 1.44 (t, *J* = 7.0 Hz, 3H). HRMS (ESI): exact mass calculated for $C_{22}H_{19}N_5O$: 369.1662, found 370.1660 $(M+1)^+$.

*5-(2-(3-Aminophenyl)-1H-pyrrolo[2,3-b]pyridin-4-yl)-1H-indazol-3-amine (**5c**).* The crude residue was purified by flash chromatography (90% EtOAc in petroleum ether) to obtain the titled product as beige solid (21 mg, 17%). ^{1}H NMR (500 MHz, DMSO-*d*$_6$) δ 12.45 (s, 1H), 8.37 (s, 1H), 8.30 (d, *J* = 7.8 Hz, 1H), 7.84 (d, *J* = 8.0 Hz 2H), 7.54 – 7.47 (m, 3H), 7.34 (t, *J* = 8.0 Hz, 1H), 7.26 (d, *J* = 5.0 Hz, 1H), 7.15 (s, 1H), 5.57 (s, 2H). LC-MS: exact mass calculated for $C_{20}H_{16}N_6$: 340.14, found 341.20 $(M+1)^+$.

*3-(4-(3-Amino-1H-indazol-5-yl)-1H-pyrrolo[2,3-b]pyridin-2-yl)phenol (**5d**).* The crude residue was purified by column chromatography (10% MeOH in EtOAc) to obtain the titled product as brown solid (49 mg, 39%). ^{1}H NMR (400 MHz, DMSO-*d*$_6$) δ 12.16 (s, 1H), 11.56 (s, 1H), 9.56 (s, 1H), 8.25 – 8.24 (m, 2H), 7.71 (dd, *J* = 8.5, 2.1 Hz, 1H), 7.34 (s, 1H), 7.44 – 7.38 (m, 2H), 7.28 – 7.24 (m, 1H), 7.19 (d, *J* = 5.3 Hz, 1H), 6.77 (d, *J* = 8.0 Hz, 1H), 7.09 (d, *J* = 2.2 Hz, 1H), 5.57 (s, 2H). HRMS (ESI): exact mass calculated for $C_{19}H_{15}N_5O$: 341.1301, found 341.1401 $(M+1)^+$.

*5-(2-(3-Methoxyphenyl)-1H-pyrrolo[2,3-b]pyridin-4-yl)-1H-indazol-3-amine (**5e**).* The crude residue was purified by column chromatography (10% MeOH in EtOAc) to afford the titled compound as a brown solid (58 mg, 44%). ^{1}H NMR (500 MHz, DMSO) δ 12.43 (s, 1H), 8.39 (s, 1H), 8.33 (d, *J* = 5.1 Hz, 1H), 7.89 (d, *J* = 8.7 Hz, 1H), 7.61 – 7.57 (m, 2H), 7.53 (d, *J* = 8.7 Hz, 1H), 7.41 (t, *J* = 8.2 Hz, 1H), 7.28 (d, *J* = 5.2 Hz, 1H), 7.26 (s, 1H), 6.96 (dd, *J* = 8.2, 2.4 Hz, 1H), 3.87 (s, 3H). LC-MS: exact mass calculated for $C_{21}H_{17}N_5O$: 355.14, found 356.14 $(M+1)^+$.

*3-(4-(3-Amino-1H-indazol-5-yl)-1H-pyrrolo[2,3-b]pyridin-2-yl)-5-fluorophenol (**5f**).* The crude residue was purified by column chromatography (10% MeOH in EtOAc) to afford the titled compound as yellow solid (29 mg, 21.9%) ^{1}H NMR (DMSO-*d*$_6$): δ 12.35 (br s, 1H), 10.11 (s, 1H), 8.35 (s, 1H), 8.32 (d, *J* = 4.8 Hz, 1H), 7.85 (dd, *J* = 1.6, 8.8 Hz, 1H), 7.49 (d, *J* = 8.4 Hz, 1H), 7.29 – 7.26 (m, 1H), 7.26 (d, *J* = 4.8 Hz, 1H), 7.24 (t, *J* = 1.6 Hz, 1H), 7.20 (d, *J* = 2.0 Hz, 1H). HRMS (ESI): exact mass calculated for $C_{20}H_{14}FN_5O$: 359.1255, found 360.1252 $(M+1)^+$.

*5-(2-(3-Fluoro-5-methoxyphenyl)-1H-pyrrolo[2,3-b]pyridin-4-yl)-1H-indazol-3-amine (**5g**).* The crude solid was purified by column chromatography (90% EtOAc in petroleum ether) to give the titled compound as a yellow solid (62 mg, 45%). ^{1}H NMR (400 MHz, DMSO-*d*$_6$) δ 12.31 (s, 1H), 11.59 (s, 1H), 8.29 (d, *J* = 4.9 Hz, 1H), 8.24 (s, 1H), 7.73 (d, *J* = 8.7 Hz, 1H), 7.50 – 7.43 (m, 2H), 7.40 (d, *J* = 8.7 Hz, 1H), 7.32 (s, 1H), 7.22 (d, *J* = 5.0 Hz, 1H), 6.85 – 6.77 (m, 1H), 5.58 (s, 2H), 3.86 (s, 3H). LC-MS: exact mass calculated for $C_{21}H_{16}FN_5O$: 373.13, Found 374.3 $(M+1)^+$.

*3-(4-(3-Amino-1H-indazol-5-yl)-1H-pyrrolo[2,3-b]pyridin-2-yl)benzoic acid (**5h**).* The crude residue was purified by column chromatography (90% EtOAc in petroleum ether) to obtain the titled compound as beige solid (34 mg, 25 %). ^{1}H NMR (500 MHz, DMSO-*d*$_6$) δ 12.48 (s, 1H), 8.53 (s, 1H), 8.34 (s, 1H), 8.30 (d, *J* = 5.0 Hz, 1H), 8.23 (d, *J* = 8.1 Hz, 1H), 7.92 (d, *J* = 7.0 Hz, 1H), 7.85 (d, *J* = 8.5 Hz 1H), 7.61 (t, *J* = 7.8 Hz, 1H), 7.49 (d, *J* = 8.7 Hz, 1H), 7.26 – 7.24 (m, 2H), 5.54 (s, 2H). LC-MS: exact mass calculated for $C_{21}H_{15}N_5O_2$: 369.12, found 370.2 $(M+1)^+$.

*3-(4-(3-Amino-1H-indazol-5-yl)-1H-pyrrolo[2,3-b]pyridin-2-yl)benzamide (**5i**).* The crude solid was purified by column chromatography (10% MeOH in EtOAc) to obtain the titled compound as beige solid (23 mg, 17 %). ^{1}H NMR (500 MHz, DMSO-*d*$_6$) δ 12.48 (s, 1H), 8.53 (s, 1H), 8.34 (s, 1H), 8.30 (d, *J* = 5.0 Hz, 1H), 8.23 (d, *J* = 8.1 Hz, 1H), 7.92 (d, *J* = 7.0 Hz, 1H), 7.85 (d, *J* = 8.5 Hz 1H), 7.61 (t, *J* = 7.8 Hz, 1H), 7.49 (d, *J* = 8.7 Hz, 1H), 7.26 – 7.23 (m, 2H), 5.54 (s, 2H). LC-MS: exact mass calculated for $C_{21}H_{16}N_6O$: 368.12, found 369.2 $(M+1)^+$.

*N-(3-(4-(3-Amino-1H-indazol-5-yl)-1H-pyrrolo[2,3-b]pyridin-2-yl)phenyl)methanesulfonamide (**5j**).* The crude residue was purified by column chromatography (10% MeOH in EtOAc) and triturated with Et_2O to afford the titled compound as an off-white solid (14 mg, 9%). ^{1}H NMR (DMSO-*d*$_6$): δ 12.32 (br s, 1H), 11.57 (br s, 1H), 9.83 (br s, 1H), 8.27 (d, *J* = 5.2 Hz, 1H), 7.70 – 7.68 (m, 3H), 8.23 (s, 1H), 7.42 (q, *J* = 8.3 Hz, 2H), 7.19 – 7.16 (m, 2H), 7.07 (d, *J* = 2.0 Hz, 1H), 5.56 (br s, 2H), 3.09 (s, 3H). HRMS (ESI): exact mass calculated for $C_{21}H_{18}N_6O_2S$: 418.1285, found 419.1287 $(M+1)^+$.

*5-(2-(3-(Methylsulfonyl)phenyl)-1H-pyrrolo[2,3-b]pyridin-4-yl)-1H-indazol-3-amine (**5k**).* The crude solid was purified by column chromatography (10% MeOH/EtOAc) to afford the titled compound as an off-white solid (34 mg, 23%). ^{1}H NMR (DMSO-*d*$_6$): δ 12.43 (br s, 1H), 11.57 (br s, 1H), 8.27 (t, *J* = 1.2 Hz, 1H), 8.32 – 8.30 (m, 2H), 8.25 (s, 1H), 7.87 (d, *J* = 6.4 Hz, 1H), 7.75 – 7.73 (m, 2H), 7.42 (d, *J* = 6.8 Hz,

1H), 7.37 (d, *J* = 1.6 Hz, 1H), 7.23 (d, *J* = 4.0 Hz, 1H), 3.31 (s, 3H). HRMS (ESI): exact mass calculated for $C_{21}H_{17}N_5O_2S$: 403.1132, found 404.1172 $(M+1)^+$.

*N-(3-(4-(3-Amino-1H-indazol-5-yl)-1H-pyrrolo[2,3-b]pyridin-2-yl)phenyl)-3-methoxypropanamide (**5l**).* The crude residue was purified by column chromatography (90% EtOAc in petroleum ether) to obtain the titled compound as beige solid (27 mg, 17%). ^{1}H NMR (500 MHz, DMSO-*d*6) δ 12.45 (s, 1H), 10.08 (s, 1H), 8.32 (d, *J* = 6.50 Hz, 1H), 8.15 (s, 1H), 7.88 – 7.85 (m, 1H), 7.67 (d, *J* = 7.8 Hz, 1H), 7.60 (d, *J* = 7.8 Hz, 1H), 7.54 (d, *J* = 8.0 Hz, 1H), 7.43 (t, *J* = 10.0 Hz, 1H), 7.25 (d, *J* = 6.5 Hz, 1H), 7.06 (d, *J* = 1.8 Hz, 1H), 3.63 (t, *J* = 8.0 Hz, 2H), 3.25 (s, 3H), 2.58 (t, *J* = 8.0 Hz, 2H). LC-MS: exact mass calculated for $C_{24}H_{22}N_6O_2$: 426.18, found 427.20 $(M+1)^+$.

*5-(2-(3-((Cyclopentylamino)methyl)phenyl)-1H-pyrrolo[2,3-b]pyridin-4-yl)-1H-indazol-3-amine (**5n**).* The crude solid was purified by column chromatography (1% Et_3N and 20% MeOH in EtOAc) to give the titled compound as a brown solid (12.5 mg, 8%). ^{1}H NMR (400 MHz, DMSO-*d*6) δ 12.20 (br s, 1H), 8.28 – 8.21 (m, 2 H), 7.94 (s, 1 H), 7.84 (d, *J*=7.47 Hz, 1 H), 7.72 (d, *J*=8.79 Hz, 1 H), 7.44 – 7.37 (m, 2 H), 7.32 (d, *J*= 7.47 Hz, 1 H), 7.21 – 7.15 (m, 2 H), 5.56 (s, 2 H), 3.74 (s, 2 H), 3.04 – 3.01 (m, 1 H), 1.74 – 1.72 (m, 2 H), 1.65 – 1.64 (m, 2 H), 1.46 – 1.45 (m, 2 H), 1.38 – 1.36 (m, 2 H). ^{13}C NMR (100 MHz, DMSO-*d*6) δ 151.02, 150.43, 147.82, 143.71, 141.65, 141.75, 139.06, 131.98, 129.18, 128.35, 127.27, 125.70, 124.17, 120.91, 119.11, 115.07, 115.05, 110.53, 109.70, 97.35, 59.11, 52.20, 33.02, 24.18. HRMS (ESI): exact mass calculated for $C_{26}H_{26}N_6$: 422.2292, found 423.2289 $(M+1)^+$.

*5-(2-(3-Isobutoxyphenyl)-1H-pyrrolo[2,3-b]pyridin-4-yl)-1H-indazol-3-amine (**5o**).* The crude residue was purified by column chromatography (90% EtOAc in petroleum ether) to afford the titled compound as an off-white solid (28 mg, 19%). ^{1}H NMR (DMSO-*d*6): δ 12.19 (br s, 1H), 11.55 (br s, 1H), 8.26 (d, *J* = 4.8 Hz, 1H), 8.24 (s, 1H), 7.72 (dd, *J* = 1.6, 8.8 Hz, 1H), 7.57 – 7.54 (m, 2H), 7.39 – 7.37 (m, 2H), 7.20 – 7.18 (m, 2H), 6.91 – 6.89 (m, 1H), 5.54 (br s, 2H), 3.85 (d, *J* = 6.4 Hz, 2H), 2.06 – 2.04 (m, 1H), 1.02 (d, *J* = 6.8 Hz, 6H). HRMS (ESI): exact mass calculated for $C_{24}H_{23}N_5O$: 397.1975, found 398.1970 $(M+1)^+$.

*N-(3-(4-(3-Amino-1H-indazol-5-yl)-1H-pyrrolo[2,3-b]pyridin-2-yl)phenyl)-4-methylbenzensulfonamide (**5t**).* The crude residue was purified by column chromatography (10% MeOH in EtOAc) and triturated with Et_2O to afford the titled compound as an off-white solid (53 mg, 29%). ^{1}H NMR (DMSO-*d*6): δ 12.25 (br s, 1H), 11.58 (br s, 1H), 10.32 (s, 1H), 8.27 (d, *J* = 5.2 Hz, 1H), 8.22 (s, 1H), 7.69 – 7.66 (m, 3H), 7.61 – 7.59 (m, 2H), 7.42 (d, *J* = 8.4 Hz, 1H), 7.32 (t, *J* = 8.0 Hz, 3H), 7.18 (d, *J* = 4.8 Hz, 1H), 7.05 (dd, *J* = 1.2, 8.0 Hz, 1H), 6.92 (d, *J* = 2.0 Hz, 1H), 5.57 (br s, 2H), 3.33 (s, 3H). HRMS (ESI): exact mass calculated for $C_{27}H_{22}N_6O_2S$: 494.1598, found 495.1592 $(M+1)^+$.

*5-(2-(3-((Phenylamino)methyl)phenyl)-1H-pyrrolo[2,3-b]pyridin-4-yl)-1H-indazol-3-amine (**5p**).* The crude residue was purified by column chromatography (90% EtOAc in petroleum ether) followed by HPLC to give the titled compound as a yellow solid (25 mg, 16%). ^{1}H NMR (400 MHz, DMSO-*d*6) δ 12.22 (br s, 1 H), 8.26 (d, *J*=5.27 Hz, 1 H), 7.85 (d, *J*=7.47 Hz, 1 H), 7.71 (d, *J*=9.67 Hz, 1 H), 7.41 (d, *J*=7.91 Hz, 2 H), 7.36 – 7.32 (m, 1 H), 7.19 (d, *J*=4.83 Hz, 1 H), 7.16 (d, *J*=1.32 Hz, 1 H), 7.04 (t, *J*=7.69 Hz, 3 H), 6.61 (d, *J*= 7.91 Hz, 3 H), 6.54 – 6.48 (m, 1 H), 6.26 – 6.20 (m, 1 H), 5.56 (s, 2 H), 4.31 (d, *J*= 5.71 Hz, 2 H). HRMS (ESI): exact mass calculated for $C_{27}H_{22}N_6$: 430.199, found 431.1978 $(M+1)^+$.

*5-(2-(2-(Benzyloxy)phenyl)-1H-pyrrolo[2,3-b]pyridin-4-yl)-1H-indazol-3-amine (**5q**).* The crude solid was purified by column chromatography (10% MeOH in EtOAc) and triturated with Et_2O to afford the titled compound as an off-white solid (46 mg, 29%). ^{1}H NMR (DMSO-*d*6): δ 11.85 (br s, 1H), 11.54 (br s, 1H), 8.24 (d, *J* = 4.8 Hz, 1H), 8.16 (s, 1H), 7.92 (dd, *J* = 1.6, 7.6 Hz, 1H), 7.57 (dd, *J* = 1.6, 8.8 Hz, 1H), 7.53 – 7.52 (m, 2H), 7.35 – 7.30 (m, 1H), 7.27 – 7.24 (m, 6H), 7.22 (d, *J* = 4.8 Hz, 1H), 7.11 – 6.98 (m, 1H), 5.50 (br s, 2H), 5.24 (s, 2H). HRMS (ESI): exact mass calculated for $C_{27}H_{21}N_5O$: 431.1719, found 432.1814 $(M+1)^+$.

*(3-(4-(3-Amino-1H-indazol-5-yl)-1H-pyrrolo[2,3-b]pyridin-2-yl)phenyl)(4-methylpiperazin-1-yl)methanone (**5v**).* The resulting solid was washed with hot DCM to afford the titled compound as a brown solid (125 mg, 75%). ^{1}H NMR (DMSO-*d*6): δ 12.29 (br s, 1H), 11.59 (br s, 1H), 8.28 (d, *J* = 4.8 Hz, 1H), 8.26 (s, 1H), 8.06 – 8.03 (m, 1H), 8.00 (s, 1H), 7.73 – 7.70 (m, 1H), 7.54 (t, *J* = 7.8 Hz, 1H), 7.40 – 7.38 (m, 1H), 7.28 (d, *J* = 2.0 Hz, 1H), 7.21 (d, *J* = 5.2 Hz, 1H), 5.58 (s, 2H), 3.65 – 3.62 (m, 2H), 2.41 – 2.38 (m, 2H), 2.30 – 2.27 (m, 2H), 2.20 (s, 3H). HRMS (ESI): exact mass calculated for $C_{26}H_{25}N_7O$: 451.2193, found 452.2191 $(M+1)^+$.

*5-(2-(3-(Pyridin-2-ylmethoxy)phenyl)-1H-pyrrolo[2,3-b]pyridin-4-yl)-1H-indazol-3-amine (**5w**).* The crude residue was purified by column chromatography (5% MeOH in EtOAc) to obtain the titled

compound as beige solid (58 mg, 36%). ^{1}H NMR (400 MHz, DMSO-d_6) δ 12.24 (s, 1H), 11.57 (s, 1H), 8.60 (d, *J* = 4.3 Hz, 1H), 8.26 (d, *J* = 5.0 Hz, 1H), 8.24 (s, 1H), 7.88 – 7.84 (m, 1H), 7.75 – 7.69 (m, 2H), 7.62 – 7.57 (m, 2H), 7.44 – 7.32 (m, 3H), 7.22 (d, *J* = 1.9 Hz, 1H), 7.20 (d, *J* = 4.9 Hz, 1H), 7.02 (d, *J* = 8.0 Hz, 1H), 5.57 (s, 2H), 5.29 (s, 2H). HRMS (ESI): exact mass calculated for $C_{26}H_{20}N_6O$: 432.1777, found 433.1767 (M+1)$^+$.

*5-(2-(3-((Tetrahydro-2H-pyran-4-yl)methoxy)phenyl)-1H-pyrrolo[2,3-b]pyridin-4-yl)-1H-indazol-3-amine (**5x**).* The crude solid was purified by column chromatography (10% MeOH in EtOAc) to obtain the titled compound as white solid (26 mg, 16%). ^{1}H NMR (400 MHz, DMSO-d_6) δ 12.22 (s, 1H), 11.57 (s, 1H), 8.26 (d, *J* = 5.0 Hz, 1H), 8.24 (s, 1H), 7.72 (dd, *J* = 8.8, 1.2 Hz, 1H), 7.58 – 7.56 (m, 2H), 7.40 (d, *J* = 8.8 Hz, 1H), 7.38 – 7.34 (m, 1H), 7.21 – 7.18 (m, 2H), 6.92 (d, *J* = 7.1 Hz, 1H), 5.56 (s, 2H), 3.94 (d, *J* = 6.4 Hz, 2H), 3.90 (dd, *J* = 11.5, 3.4 Hz, 2H), 3.42 – 3.34 (m, 2H), 2.12 – 1.95 (m, 1H), 1.72 (d, *J* = 11.2 Hz, 2H), 1.41 – 1.32 (m, 2H). LC-MS: exact mass calculated for $C_{26}H_{25}N_5O_2$: 439.20, found 440.3 (M+1)$^+$.

*5-(2-(3-(2-Morpholinoethoxy)phenyl)-1H-pyrrolo[2,3-b]pyridin-4-yl)-1H-indazol-3-amine (**5y**).* The crude solid was purified by column chromatography (10% MeOH in EtOAc) to obtain the titled compound as white solid (123 mg, 73%). ^{1}H NMR (400 MHz, DMSO-d_6) δ 12.20 (s, 1H), 11.57 (s, 1H), 8.26 (d, *J* = 5.0 Hz, 1H), 8.24 (s, 1H), 7.73 (d, *J* = 8.8 Hz, 1H), 7.59 – 7.56 (m, 2H), 7.40 (d, *J* = 8.4 Hz, 1H), 7.38 – 7.34 (m, 1H), 7.22 (d, *J* = 2.1 Hz, 1H), 7.20 (d, *J* = 4.9 Hz, 1H), 6.92 (d, *J* = 8.4 Hz, 1H), 5.56 (s, 2H), 4.19 (t, *J* = 5.7 Hz, 2H), 3.63 – 3.56 (m, 4H), 2.73 (t, *J* = 5.7 Hz, 2H), 2.48-2.51 (m, 4H). LC-MS: exact mass calculated for $C_{26}H_{26}N_6O_2$: 454.21, found 455.3 (M+1)$^+$.

*5-(2-(3-(Benzyloxy)phenyl)-1H-pyrrolo[2,3-b]pyridin-4-yl)-1H-indazol-3-amine (**5r**).* [SU1261] The crude residue was purified by column chromatography (90% EtOAc in petroleum ether) to afford the titled compound as an off-white solid (92 mg, 58%). ^{1}H NMR (500 MHz, DMSO-d_6) δ 12.21 (s, 1H), 11.56 (s, 1H), 8.27 (d, J = 5.0 Hz, 1H), 8.25 (s, 1H), 7.73 (d, J = 8.4 Hz, 1H), 7.69 (s, 1H), 7.59 (d, J = 7.6 Hz, 1H), 7.51 (d, J = 7.6 Hz, 2H), 7.43 – 7.39 (m, 4H), 7.38 – 7.32 (m, 2H), 7.23 – 7.19 (m, 2H), 6.99 (d, J = 8.4 Hz, 1H), 5.55 (s, 2H), 5.21 (s, 2H). ^{13}C NMR (126 MHz, DMSO-d_6) δ 158.85, 150.43, 149.89, 143.32, 141.19, 141.08, 138.05, 137.04, 133.00, 129.96, 128.44, 127.87, 127.82, 127.70, 126.69, 120.35, 118.40, 118.06, 114.62, 114.50, 114.46, 111.62, 109.95, 97.28, 69.36. HRMS (ESI): exact mass calculated for $C_{27}H_{21}N_5O$: 431.1746, found 432.1817 (M+1)$^+$.

*5-(2-(3-((4-Methoxybenzyl)oxy)phenyl)-1H-pyrrolo[2,3-b]pyridin-4-yl)-1H-indazol-3-amine (**5z**).* The crude residue was purified by recrystallization using MeOH/DCM and hexane to afford the titled compound as an off-white solid (109 mg, 64%). ^{1}H NMR (DMSO-d_6): δ 12.19 (br s, 1H), 11.55 (br s, 1H), 8.26 (d, *J* = 5.2 Hz, 1H), 8.24 (s, 1H), 7.72 (dd, *J* = 1.2, 8.4 Hz, 1H), 7.66 (s, 1H), 7.58 (d, *J* = 8.0 Hz, 1H), 7.39 – 7.34 (m, 4H), 7.20 – 7.16 (m, 2H), 6.98 – 6.95 (m, 3H), 5.54 (br s, 2H), 5.11 (s, 2H), 3.75 (s, 3H). HRMS (ESI): exact mass calculated for $C_{28}H_{23}N_5O_2$: 461.1925 found 462.1922 (M+1)$^+$.

*5-(2-(3-(Morpholinosulfonyl)phenyl)-1H-pyrrolo[2,3-b]pyridin-4-yl)-1H-indazol-3-amine (**5aa**).* The resulting solid was suspended in EtOAc and filtered through a pad of celite and concentrated under reduced pressure to afford the titled compound as a pale-yellow solid (17.5 mg, 10%). ^{1}H NMR (DMSO-d_6): δ 12.53 (br s, 1H), 11.58 (br s, 1H), 8.32 – 8.28 (m, 2H), 8.26 (s, 1H), 7.74 – 7.69 (m, 4H), 7.41 (d, *J* = 8.8 Hz, 1H), 7.34 (s, 1H), 7.23 (d, *J* = 4.8 Hz, 1H), 5.55 (br s, 2H), 3.65 – 3.61 (m, 4H), 2.96 – 2.92 (m, 4H). HRMS (ESI): exact mass for $C_{24}H_{22}N_6O_3S$: 474.1547, found 475.1548 (M+1)$^+$.

*5-(2-(4-Benzyloxy)phenyl)-1H-pyrrolo[2,3-b]pyridin-4-yl)-1H-indazol-3-amine (**5s**).* The resulting solid was triturated with hexane and Et_2O to afford the titled compound as a pale-yellow solid (75 mg, 47%). ^{1}H NMR (DMSO-d_6): δ 12.09 (br s, 1H), 11.53 (br s, 1H), 8.22 – 8.18 (m, 2H), 7.92 (d, *J* = 8.8 Hz, 1H), 7.71 (d, *J* = 1.6, 8.8 Hz, 1H), 7.49 – 7.43 (m, 2H), 7.41 – 7.37 (m, 3H), 7.34 – 7.30 (m, 1H), 7.18 (d, *J* = 4.8 Hz, 1H), 7.12 (d, *J* = 8.8 Hz, 2H), 7.07 (d, *J* = 2.4 Hz, 1H), 5.53 (br s, 2H), 5.18 (s, 2H). HRMS (ESI): exact mass calculated for $C_{27}H_{21}N_5O$: 431.1719, found 432.1813 (M+1)$^+$.

*N-(4-(4-(3-Amino-1H-indazol-5-yl)-1H-pyrrolo[2,3-b]pyridin-2-yl)phenyl)-4-methylbenzene sulfonamide (**5u**).* The crude residue was purified by column chromatography (10% MeOH in EtOAc) and triturated with Et_2O to afford the titled compound as an off-white solid (51 mg, 28%). ^{1}H NMR (DMSO-d_6): δ 12.10 (br s, 1H), 11.55 (br s, 1H), 10.40 (br s, 1H), 8.22 (d, *J* = 4.8 Hz, 1H), 8.20 (s, 1H), 7.83 (d, *J* = 8.8 Hz, 2H), 7.68 (dd, *J* = 2.0, 8.8 Hz, 3H), 7.37 (t, *J* = 9.2 Hz, 3H), 7.17 (s, 1H), 7.15 (d, *J* = 4.0 Hz, 1H), 7.06 (d, *J* = 2.0 Hz, 1H), 5.54 (br s, 2H), 2.32 (s, 3H). HRMS (ESI): calculated for $C_{27}H_{22}N_6O_2S$: 494.1598, found 495.1592 (M+1)$^+$.

*Synthesis of 3-(4-(3-Amino-1H-indazol-5-yl)-1H-pyrrolo[2,3-b]pyridin-2-yl)-N-(2-methoxyethyl)benzamide (**5m**).* 3-(4-(3-Amino-1H-indazol-5-yl)-1H-pyrrolo[2,3-b]pyridin-2-yl)benzoic

acid (**5h**) (0.05 g, 0.1 mmol) and 2-methoxyethan-1-amine (17 μL, 0.2 mmol) were placed in a 50 ml RBF, DMF (5 ml) was then added and the solution allowed to stir for 5 min, HCTU (123 mg, 0.3 mmol) and trimethylamine (42 μL, 0.3 mmol) were then added and the solution allowed to stir at room temperature overnight. The solvent was then removed under high vacuum and the resulting residue purified by flash chromatography (10% methanol in EtOAc with 1% triethylamine) to give the desired product as an off white solid. (105 mg, 88%), ^{1}H NMR (DMSO-d6) δ 2.08 (s, 3H), 3.28 (br s, 2H), 3.48 (br s, 2H), 5.54 (s, 2H), 7.24 (m, 2H), 7.49 (d, *J* = 8.7 Hz, 1H), 7.57 (t, *J* = 7.8 Hz, 1H), 7.79 (d, *J* = 8.5 Hz 1H), 8.12 (d, *J* = 7.0 Hz, 1H), 8.31 (d, *J* = 8.1 Hz, 1H), 8.44 (d, *J* = 5.0 Hz, 1H), 8.59 (s, 1H), 12.35 (s, 1H). LC-MS: exact mass calculated for $C_{24}H_{22}N_6O_2$: 426.18, found 427.20 $(M+1)^+$.

*General procedure for synthesis of 4-chloro-2-(substituted aryl)-1H-pyrrolo[2,3-b]pyridine (**17a-i**).*

Followed the general procedure for synthesis of compounds **15a-ac**. The crude residue was used in the next step without further purification unless otherwise stated below.

*2-(6-(Benzyloxy)pyridin-3-yl)-4-chloro-1H-pyrrolo[2,3-b]pyridine (**17d**).* The crude residue was purified by column chromatography (70% EtOAc in petroleum ether) to afford the titled compound as white solid (181 mg, 44%). ^{1}H NMR (400 MHz, DMSO-d_6) δ 12.55 (s, 1H), 8.82 (d, *J* = 2.3 Hz, 1H), 8.33 (dd, *J* = 8.7, 2.3 Hz, 1H), 8.17 (d, *J* = 5.2 Hz, 1H), 7.48 (d, *J* = 7.0 Hz, 2H), 7.42 – 7.38 (m, 2H), 7.35 (d, *J* = 7.1 Hz, 1H), 7.21 (d, *J* = 5.1 Hz, 1H), 7.05 – 6.99 (m, 2H), 5.42 (s, 2H). LC-MS: exact mass calculated for $C_{19}H_{14}{}^{35}ClN_3O$: 335.0825, found 336.1 $(M+1)^+$.

*2-(5-(Benzyloxy)pyridin-3-yl)-4-chloro-1H-pyrrolo[2,3-b]pyridine (**17e**).* The crude residue was suspended in EtOAc and filtered through a pad of celite and evaporated to give the titled product as brown solid (383 mg, 93%). ^{1}H NMR (400 MHz, DMSO-d_6) δ 12.67 (s, 1H), 8.84 (d, *J* = 1.6 Hz, 1H), 8.34 (d, *J* = 2.6 Hz, 1H), 8.22 (d, *J* = 5.2 Hz, 1H), 8.11 (dd, *J* = 2.6, 1.6 Hz, 1H), 7.52 (d, *J* = 7.1 Hz, 2H), 7.43 – 7.41 (m, 2H), 7.39 – 7.35 (m, 1H), 7.25 – 7.24 (m, 2H), 5.29 (s, 2H). LC-MS: exact mass calculated for $C_{19}H_{14}{}^{35}ClN_3O$: 335.0825, found 336.1 $(M+1)^+$.

*2-(2-(Benzyloxy)pyridin-4-yl)-4-chloro-1H-pyrrolo[2,3-b]pyridine (**17g**).* The crude solid was purified by column chromatography (90% EtOAc in petroleum ether) to give the titled product as brown solid (371 mg, 90%). ^{1}H NMR (400 MHz, DMSO-d_6) δ 12.72 (s, 1H), 8.26 – 8.24 (m, 2H), 7.63 (d, *J* = 5.7 Hz, 1H), 7.52 – 7.47 (m, 3H), 7.42 – 7.38 (m, 2H), 7.35 (d, *J* = 7.5 Hz, 1H), 7.33 (d, *J* = 1.8 Hz, 1H), 7.26 (d, *J* = 5.3 Hz, 1H), 5.41(s, 2H). LC-MS: exact mass calculated for $C_{19}H_{14}{}^{35}ClN_3O$: 335.0825, found 336.1 $(M+1)^+$.

*General procedure for synthesis of 5-(2-substituted aryl-1H-pyrrolo[2,3-b]pyridin-4-yl)-1H-indazol-3-amine (**6a–i**).* Followed the general procedure for synthesis of *compounds* **5**.

*5-(2-(3-(Benzyloxy)-5-fluorophenyl)-1H-pyrrolo[2,3-b]pyridin-4-yl)-1H-indazol-3-amine (**6a**).* The crude solid was recrystallized from EtOAc to afford the titled compound as a beige solid (88 mg, 53%). ^{1}H NMR (400 MHz, DMSO-d_6) δ 12.27 (s, 1H), 11.58 (s, 1H), 8.29 (d, *J* = 4.9 Hz, 1H), 8.23 (s, 1H), 7.73 (d, *J* = 8.4 Hz, 1H), 7.61 – 7.31 (m, 9H), 7.22 (d, *J* = 4.9 Hz, 1H), 6.90 (d, *J* = 10.7 Hz, 1H), 5.56 (s, 2H), 5.22 (s, 2H). LC-MS: exact mass calculated for $C_{27}H_{20}FN_5O$: 449.1652, found 450.3 $(M+1)^+$

*5-(2-(3-(Benzyloxy)-5-((2-methoxyethoxy)methyl)phenyl)-1H-pyrrolo[2,3-b]pyridin-4-yl)-1H-indazol-3-amine (**6b**).* The crude solid was washed with water (3 mL) and hexane (3 × 3 mL) to afford the titled compound as an off-white solid (146 mg, 76%). ^{1}H NMR (400 MHz, DMSO-d_6) δ 12.23 (s, 1H), 11.57 (s, 1H), 8.26 (d, *J* = 4.9 Hz, 1H), 8.23 (s, 1H), 7.72 (d, *J* = 8.8 Hz, 1H), 7.61 (s, 1H), 7.55 (s, 1H), 7.51 (d, *J* = 7.5 Hz, 2H), 7.46 – 7.38 (m, 3H), 7.38 – 7.32 (m, 1H), 7.22 – 7.17 (m, 2H), 6.97 (s, 1H), 5.57 (s, 2H), 5.20 (s, 2H), 4.52 (s, 2H), 3.60 – 3.55 (m, 2H), 3.53 – 3.46 (m, 2H), 3.25 (s, 3H). LC-MS: exact mass calculated for $C_{31}H_{29}N_5O_3$: 519.2270, found 520.3 $(M+1)^+$

*5-(2-(3-(Benzyloxy)-5-((2-methoxyethyl)amino)phenyl)-1H-pyrrolo[2,3-b]pyridin-4-yl)-1H-indazol-3-amine (**6c**).* The crude product was purified by HPLC to afford the titled compound as a pale-yellow solid (26 mg, 14%). ^{1}H NMR (500 MHz, DMSO-d_6) δ 12.23 (s, 1H), 8.31 (s, 1H), 8.27 (d, *J* = 4.9 Hz, 1H), 7.82 (d, *J* = 8.4 Hz, 1H), 7.49 – 7.32 (m, 6H), 7.21 (d, *J* = 4.9 Hz, 1H), 7.07 (d, *J* = 1.7 Hz, 1H), 6.90 (s, 1H), 6.83 (s, 1H), 5.11 (s, 2H), 3.52 – 3.48 (m, 2H), 3.38 – 3.25 (m, 7H). LC-MS: exact mass calculated for $C_{20}H_{28}N_6O_2$: 504.2274, found 505.30 $(M+1)^+$

*5-(2-(6-(Benzyloxy)pyridin-3-yl)-1H-pyrrolo[2,3-b]pyridin-4-yl)-1H-indazol-3-amine (**6d**).* The crude solid was purified by column chromatography (10% MeOH in EtOAc) to afford the title compound as a beige solid (43 mg, 27%). ^{1}H NMR (400 MHz, DMSO-d_6) δ 12.27 (s, 1H), 11.57 (s, 1H), 8.81 (d, *J* = 2.3 Hz, 1H), 8.32 (dd, *J* = 8.4, 2.3 Hz, 1H), 8.25 (d, *J* = 5.0 Hz, 1H), 8.23 (s, 1H), 7.72 (d, *J* = 8.9 Hz, 1H), 7.50 –

7.46 (m, 2H), 7.42 – 7.36 (m, 3H), 7.34 (d, *J* = 7.4 Hz, 1H), 7.21 – 7.19 (m, 2H), 7.01 (d, *J* = 8.9 Hz, 1H), 5.56 (s, 2H), 5.42 (s, 2H). HRMS (ESI): exact mass calculated for $C_{26}H_{20}N_6O$: 432.1699, found 433.1777 (M+1)$^+$.

*5-(2-(5-(Benzyloxy)pyridin-3-yl)-1H-pyrrolo[2,3-b]pyridin-4-yl)-1H-indazol-3-amine (**6e**).* The crude solid was purified by column chromatography (1% Et_3N and 10% MeOH in EtOAc) to afford the titled compound as a beige solid (86 mg, 54%) ^{1}H NMR (400 MHz, DMSO-d_6) δ 12.37 (s, 1H), 11.58 (s, 1H), 8.85 (d, *J* = 1.6 Hz, 1H), 8.34 – 8.27 (m, 2H), 8.24 (s, 1H), 8.08 (s, 1H), 7.74 (d, *J* = 8.6 Hz, 1H), 7.52 (d, *J* = 7.5 Hz, 2H), 7.47 – 7.35 (m, 5H), 7.23 (d, *J* = 5.0 Hz, 1H), 5.57 (s, 2H), 5.28 (s, 2H). HRMS (ESI): exact mass calculated for $C_{26}H_{20}N_6O$: 432.1699, found 433.1777 (M+1)$^+$.

*5-(2-(2-Fluoro-6-((phenylamino)methyl)pyridin-4-yl)-1H-pyrrolo[2,3-b]pyridin-4-yl)-1H-indazol-3-amine (**6f**).* The crude product was purified by HPLC to afford the titled compound as a pale-yellow solid (65 mg, 39%). ^{1}H NMR (500 MHz, DMSO-d_6) δ 12.38 (s, 1H), 8.49 (t, *J* = 5.6 Hz, 1H), 8.35 (d, *J* = 5.6 Hz, 1H), 8.29 (s, 1H), 7.80 (d, *J* = 8.4 Hz, 1H), 7.51 (d, *J* = 8.5 Hz, 1H), 7.49 (d, *J* = 8.5 Hz, 1H), 7.26 (d, *J* = 8.5 Hz, 1H), 7.17 (s, 1H), 7.07 (t, *J* = 8.5 Hz, 1H), 6.62 – 6.56 (m, 3H), 4.37 (s, 2H). LR-MS: exact mass calculated for $C_{26}H_{20}FN_7$: 449.1764, found 450.20 (M+1)$^+$.

*5-(2-(2-(Benzyloxy)pyridin-4-yl)-1H-pyrrolo[2,3-b]pyridin-4-yl)-1H-indazol-3-amine (**6g**) [SU1349].* The crude product was purified by column chromatography (10% MeOH in EtOAc) to afford the titled compound as white solid (78 mg, 49%). ^{1}H NMR (500 MHz, DMSO-*d*) δ 12.41 (s, 1H), 11.58 (s, 1H), 8.34 (d, *J* = 4.9 Hz, 1H), 8.25 (s, 1H), 8.23 (d, *J* = 5.5 Hz, 1H), 7.74 (d, J = 8.0 Hz, 1H), 7.62 (d, *J* = 5.5 Hz, 1H), 7.52 – 7.46 (m, 4H), 7.43 – 7.37 (m, 3H), 7.34 (d, *J* = 8.0 Hz, 1H), 7.25 (d, *J* = 4.9 Hz, 1H), 5.56 (s, 2H), 5.41 (s, 2H).). ^{13}C NMR (126 MHz, DMSO-d_6) δ 163.97, 150.58, 149.94, 147.39, 144.70, 142.03, 141.78, 141.26, 137.33, 135.09, 128.36, 127.94, 127.75, 127.32, 126.72, 120.50, 117.98, 114.71, 114.64, 113.70, 110.03, 105.86, 100.25, 67.03. HRMS (ESI): exact mass calculated for $C_{26}H_{20}N_6O$: 432.1699, found 433.1777 (M+1)$^+$.

*5-(2-(2-(Benzylthio)pyridin-4-yl)-1H-pyrrolo[2,3-b]pyridin-4-yl)-1H-indazol-3-amine (**6h**).* The crude solid was purified by HPLC to afford the titled compound as a yellow solid (33 mg, 20%). ^{1}H NMR (500 MHz, DMSO-d_6) δ 12.51 (s, 1H), 8.51 (d, *J* = 5.3 Hz, 1H), 8.36 (d, *J* = 4.9 Hz, 1H), 8.32 (d, *J* = 1.5 Hz, 1H) 7.92 (s, 1H), 7.85 – 7.80 (m, 1H), 7.72 (dd, *J* = 5.3, 1.6 Hz, 1H), 7.50 (d, *J* = 2.2 Hz, 1H), 7.48 (d, *J* = 8.7 Hz, 1H), 7.46 – 7.43 (m, 2H), 7.33 – 7.29 (m, 2H), 7.27 (d, *J* = 5.0 Hz, 1H), 7.26 – 7.21 (m, 1H), 4.50 (s, 2H). LR-MS: exact mass calculated for $C_{26}H_{20}N_6S$: 448.1470, found 449.2 (M+1)$^+$.

*5-(2-(2-(Benzylthio)-6-fluoropyridin-4-yl)-1H-pyrrolo[2,3-b]pyridin-4-yl)-1H-indazol-3-amine (**6i**).* The crude solid was purified by HPLC to afford the titled compound as yellow (29 mg, 17%). ^{1}H NMR (500 MHz, DMSO-d_6) δ 12.56 (s, 1H), 8.40 (d, *J* = 4.9 Hz, 1H), 8.33 (s, 1H), 7.91 (s, 1H), 7.84 (dd, *J* = 8.7, 1.7 Hz, 1H), 7.61 (d, *J* = 1.8 Hz, 1H), 7.51 (d, *J* = 2.5 Hz, 3H), 7.50 – 7.46 (m, 2H), 7.34 (t, *J* = 7.5 Hz, 2H), 7.31 – 7.23 (m, 2H), 4.47 (s, 2H). LR-MS: exact mass calculated for $C_{26}H_{19}FN_6S$: 466.1376, found 467.1 (M+1)$^+$.

*Synthesis of 4-(benzyloxy)-2-bromopyridine (**20a**).* Compound **19a** (0.436 g, 0.303 mL, 2.5 mmol) was added to a mixture of compound **18a** (0.376 g, 2.16 mmol) and cesium carbonate (1.037 g, 3.182 mmol) in DMF (5 mL) under argon at 0 °C. The reaction mixture was allowed to warm to rt and stirred for 4 h. The reaction mixture was poured onto ice/water (20 mL) and extracted with EtOAc (3 × 50 mL). The combined organic layers was washed with brine (3 × 20 mL), dried over anhydrous sodium sulfate, concentrated under reduced pressure and purified by column chromatography (50% EtOAc in petroleum ether) to afford the titled compound as a light brown solid (466 mg, 82%). ^{1}H NMR (400 MHz, DMSO-d_6) δ 8.20 (d, *J* = 5.8 Hz, 1H), 7.54 – 7.34 (m, 6H), 7.11 (dd, *J* = 5.8, 2.3 Hz, 1H), 5.24 (s, 2H). LR-MS: exact mass calculated for $C_{12}H_{10}{}^{79}BrNO$: 262.9946, found 264.0 (M+1)$^+$.

*General procedure for synthesis of 4-bromo-2-(aryloxy)pyridine (**20b,c**).* Potassium *tert*-butoxide (0.58 g, 1.36 mmol) in anhydrous THF (10 mL)was added to a solution of compound **18b** (0.2 g, 1.136 mmol) in anhydrous THF (5 mL) at 0 °C. Compounds **19b,c** (1.36 mmol) were then added, and the resulting mixtures allowed to warm to rt and stirred for 4 h. The reaction mixtures were poured onto ice/water (20 mL) and extracted with EtOAc (3 × 50 mL). The combined organic layers was washed with brine (3 × 20 mL), dried over anhydrous sodium sulfate and concentrated under reduced pressure. The crude residue was used in the next step without further purification unless otherwise stated below.

*4-Bromo-2-(pyridin-4-ylmethoxy)pyridine (**20b**).* The crude residue was purified by column chromatography (50% EtOAc in petroleum ether) to afford the titled compound as a light brown oil (240 mg, 80%). ^{1}H NMR (500 MHz, DMSO-d_6) δ 8.56 (ddd, *J* = 4.8, 1.8, 0.9 Hz, 1H), 8.07 (d, *J* = 5.4 Hz, 1H), 7.81 (td, *J* = 7.7, 1.8 Hz, 1H), 7.45 (dt, *J* = 7.9, 1.0 Hz, 1H), 7.33 (ddd, *J* = 7.6, 4.9, 1.2 Hz, 1H), 7.29 – 7.24 (m, 2H), 5.44 (s, 2H). LR-MS: exact mass calculated for $C_{11}H_9{}^{79}BrN_2O$: 263.9898, found 265.0 (M+1)$^+$.

General procedure for synthesis of 2-(substituted aryl)-4,4,5,5-tetramethyl-1,3,2-dioxaborolane (**21a-c**). A suspension of **20a-c** (6.3747 mmol), [1,1′-bis(diphenylphosphino)ferrocene] dichloropalladium(II) (0.238 g, 0.325 mmol), potassium acetate (1.918 g, 19.541 mmol), and bis(pinacolato)diboron (2.15 g, 8.466 mmol) in dioxane (9 mL) and water (1 mL) under argon was allowed to stir at 110 °C for 18 h. The reaction was cooled and diluted with EtOAc (30 mL), washed with water (3 × 20 mL). The organic layer was dried over anhydrous sodium sulfate and concentrated under reduced pressure. The crude residue was used in the next step without further purification unless otherwise stated below.

*2-(Pyridin-4-ylmethoxy)-4-(4,4,5,5-tetramethyl-1,3,2-dioxaborolan-2-yl)pyridine (**21b**).* The crude residue was purified by column chromatography on (70% EtOAc in petroleum ether) to afford the titled compound as a brown solid (978 mg, 49%). ^{1}H NMR (500 MHz, DMSO-*d*$_6$) δ 8.64 (s, 2H), 8.18 (dd, *J* = 5.0, 0.8 Hz, 1H), 7.45 (s, 2H), 7.17 (dd, *J* = 5.0, 0.8 Hz, 1H), 7.11 (s, 1H), 5.43 (s, 2H), 1.30 (s, 12H). LR-MS: exact mass calculated for $C_{17}H_{21}BN_2O_3$: 312.1645, found 313.0 (M+1)$^+$.

*2-Phenethoxy-4-(4,4,5,5-tetramethyl-1,3,2-dioxaborolan-2-yl)pyridine (**21c**).* The crude residue was purified by column chromatography (50% EtOAc in petroleum ether) to afford the titled compound as a brown solid (832 mg, 40%). ^{1}H NMR (500 MHz, DMSO-*d*$_6$) δ 8.19 (dd, *J* = 4.9, 0.9 Hz, 1H), 7.31 (d, *J* = 4.9 Hz, 4H), 7.22 (d, *J* = 4.3 Hz, 1H), 7.12 (dd, *J* = 4.9, 0.9 Hz, 1H), 6.91 (d, *J* = 1.0 Hz, 1H), 4.46 (t, *J* = 6.8 Hz, 2H), 3.02 (t, *J* = 6.8 Hz, 2H), 1.30 (s, 12H). LR-MS: exact mass calculated for $C_{19}H_{24}BNO_3$: 325.1849, found 326.1 (M+1)$^+$.

*Synthesis of 4-chloro-1-(methoxymethyl)-1H pyrrolo[2,3-b]pyridine (**23b**).* Methoxymethyl chloride (3.1659 g, 39.33 mmol)was added to a solution of 4-chloro-1*H*-pyrrolo[2,3-*b*]pyridine (**22**, 5.0 g, 32.77 mmol) and potassium carbonate (6.793 g, 49.15 mmol) in DMF (20 mL) at 0 °C. The reaction mixture was allowed to warm to rt and stirred for 18 h. The reaction was poured onto ice/water and extracted with EtOAc (3 × 50 mL). The combined organic layers were washed with brine (3 × 20 mL), dried over anhydrous sodium sulfate and concentrated under reduced pressure. The crude residue was purified by column chromatography (90% EtOAc in petroleum ether) to afford the titled compound as a beige solid (4.62 g, 72%). ^{1}H NMR (500 MHz, DMSO-*d*$_6$) δ 8.26 (d, *J* = 5.2 Hz, 1H), 7.78 (d, *J* = 3.6 Hz, 1H), 7.29 (d, *J* = 5.2 Hz, 1H), 6.61 (d, *J* = 3.6 Hz, 1H), 5.62 (s, 2H), 3.22 (s, 3H). LR-MS: exact mass calculated for $C_9H_9{}^{35}ClN_2O$: 196.0403, found 197.3 (M+1)$^+$.

*Synthesis of tert-butyl 4-chloro-1H-pyrrolo[2,3-b]pyridine-1-carboxylate (**23a**).* Di-*tert*-butyl dicarbonate (20.70 g, 21.81 mL, 94.85 mmol) was added to a solution of 4-chloro-1*H*-pyrrolo[2,3-*b*]pyridine (**22**, 9.6 g, 62.92 mmol) and 4-dimethylaminopyridine (11.59 g, 94.85 mmol) in DMF (10 mL) under argon at rt. The reaction mixture was stirred at rt for 18 h. The reaction was extracted between EtOAc and water. The combined organic layers were washed with brine (3 × 20 mL), dried over anhydrous sodium sulfate and concentrated under reduced pressure. The crude residue was purified by column chromatography (50% EtOAc in petroleum ether) to afford the titled product as a beige solid (15.69 g, 99%). ^{1}H NMR (500 MHz, DMSO-*d*$_6$) δ 8.36 (d, *J* = 5.2 Hz, 1H), 7.89 (d, *J* = 4.0 Hz, 1H), 7.44 (dd, *J* = 5.2, 1.1 Hz, 1H), 6.74 (dd, *J* = 4.1, 1.2 Hz, 1H), 1.62 (s, 9H). LR-MS: exact mass calculated for $C_{12}H_{13}{}^{35}ClN_2O_2$: 252.0666, found 253.3 (M+1)$^+$.

*General procedure for synthesis of N-Protected-4-chloro-2-iodo-1H-pyrrolo[2,3-b]pyridine (**24a,b**). n-*Butyllithium (8.7 mL, 8.70 mmol, 1 M solution in THF) was slowly added to a solution of compounds **23a,b** (7.253 mmol) in anhydrous THF at -78 °C under argon. The reaction mixture was allowed to stir for 30 minutes at 0 °C. Iodine (1.0125 g, 7.978 mmol) in THF (10 mL) was slowly added at -78 °C. The reaction mixture was allowed to warm to rt and stirred for 2 h. The reaction was poured onto ice/H_2O and extracted with EtOAc (3 × 50 mL). The combined organic layers were washed with saturated ammonium chloride (20 mL), water (20 mL) and brine (20 mL). The organic layer was dried over anhydrous sodium sulfate and removed under reduced pressure.

*4-Chloro-2-iodo-1-(methoxymethyl)-1H-pyrrolo[2,3-b]pyridine (**24b**).* The crude residue was purified by column chromatography (90% EtOAc in petroleum ether) to afford the titled compound as a light brown solid (1 g, 43%). ^{1}H NMR (500 MHz, DMSO-*d*$_6$) δ 8.21 (d, *J* = 5.2 Hz, 1H), 7.29 (d, *J* = 5.2 Hz, 1H), 7.01 (s, 1H), 5.61 (s, 2H), 3.23 (s, 3H). LR-MS: exact mass calculated for $C_9H_8{}^{35}ClIN_2O$: 321.9370, found 323.0 (M+1)$^+$.

*tert-Butyl 4-chloro-2-iodo-1H-pyrrolo[2,3-b]pyridine-1-carboxylate (**24a**).* The crude solid was purified by column chromatography (50% EtOAc in petroleum ether) to afford the titled compound as a light

brown solid (1.8 g, 65%). ^{1}H NMR (500 MHz, DMSO-d_6) δ 8.19 (t, *J* = 1.0 Hz, 1H), 7.53 (t, *J* = 1.4 Hz, 1H), 7.05 (dd, *J* = 1.7, 0.9 Hz, 1H), 1.58 (s, 9H). LR-MS: exact mass calculated for $C_{12}H_{12}{}^{35}ClIN_2O_2$: 377.9632, found 379.4 $(M+1)^+$.

*General procedure for synthesis of N-Protected-4-chloro-2-(substituted aryl)-1H-pyrrolo[2,3-b]pyridine (**25a-c**).* A mixture of compounds **21a-c** (3.139 mmol), compounds **24a,b** (2.64 mmol), bis(triphenylphosphine)palladium(II) dichloride (0.0926 g, 0.132 mmol) and cesium carbonate (2.58 g, 7.9 mmol) was suspended in dioxane (9 mL) and water (1 mL) under argon. The reaction mixture was stirred at 80 °C for 18 h. The reaction was cooled, diluted with EtOAc and washed with water (3 × 20 mL). The organic layer was dried over anhydrous sodium sulfate and concentrated under reduced pressure. The crude residue was used in the next step without further purification unless otherwise stated below.

*2-(4-(Benzyloxy)pyridin-2-yl)-4-chloro-1-(methoxymethyl)-1H-pyrrolo[2,3-b]pyridine (**25a**).* The crude residue was purified by column chromatography (1% Et_3N and 50% EtOAc in petroleum ether) to afford the titled compound as a brown solid (990 mg, 99%).^{1}H NMR (500 MHz, DMSO-d_6) δ 8.54 (d, *J* = 5.7 Hz, 1H), 8.34 (d, *J* = 5.2 Hz, 1H), 7.73 (d, *J* = 2.4 Hz, 1H), 7.54 – 7.48 (m, 2H), 7.44 (td, *J* = 7.3, 6.3, 1.5 Hz, 2H), 7.38 (dd, *J* = 8.0, 6.2 Hz, 2H), 7.25 (s, 1H), 7.10 (dd, *J* = 5.8, 2.4 Hz, 1H), 6.21 (s, 2H), 5.33 (s, 2H), 3.08 (s, 3H). LR-MS: exact mass calculated for $C_{21}H_{18}{}^{35}ClN_3O_2$: 379.1088, found 380.3 $(M+1)^+$.

*General procedure for synthesis of 5-(2-substituted aryl-1H-pyrrolo[2,3-b]pyridin-4-yl)-1H-indazol-3-amine (**6j–l**).* A mixture of compounds **25a-c** (2.373 mmol), compound **9** (0.0676 g, 0.261 mmol), [1,1′-bis(di-tert-butylphosphino)ferrocene]dichloropalladium(II) catalyst (0.0154 g, 0.0236 mmol) and cesium carbonate (0.232 g, 0.712 mmol) was suspended in dioxane (9 mL) and water (1 mL). The reaction mixture was stirred at 110 °C for 18 h. The reaction was cooled, diluted with EtOAc and washed with water (3 × 20 mL). The organic layer was dried over anhydrous sodium sulfate and concentrated under reduced pressure. The crude residue was then taken up in concentrated HCl/MeOH (4:1, 5 mL) for **6j** or TBAF (5 mL, 1M solution in THF) for **6k,l** and refluxed for 8 h. The reaction mixture was cooled and concentrated under reduced pressure.

*5-(2-(4-(Benzyloxy)pyridin-2-yl)-1H-pyrrolo[2,3-b]pyridin-4-yl)-1H-indazol-3-amine (**6j**).* The crude residue was purified by HPLC to afford the titled compound as an orange solid (35 mg, 31%) ^{1}H NMR (500 MHz, DMSO-d_6) δ 12.60 (s, 2H), 8.57 (d, *J* = 5.9 Hz, 1H), 8.41 (d, *J* = 5.1 Hz, 1H), 8.35 (d, *J* = 1.5 Hz, 1H), 7.95 (d, *J* = 2.4 Hz, 1H), 7.85 (dd, *J* = 8.7, 1.7 Hz, 1H), 7.63 (s, 1H), 7.57 – 7.51 (m, 4H), 7.48 – 7.44 (m, 2H), 7.43 – 7.38 (m, 1H), 7.32 (d, *J* = 5.0 Hz, 1H), 7.17 (dd, *J* = 6.2, 2.4 Hz, 1H), 5.37 (s, 2H). LR-MS: exact mass calculated for $C_{26}H_{20}N_6O$: 432.1699, found 433.3 $(M+1)^+$.

*5-(2-(2-(Pyridin-4-ylmethoxy)pyridin-4-yl)-1H-pyrrolo[2,3-b]pyridin-4-yl)-1H-indazol-3-amine (**6k**).* The crude residue was purified by HPLC to afford the titled compound as a bright yellow solid (11 mg, 10%). ^{1}H NMR (500 MHz, DMSO-d_6) δ 12.60 (s, 1H), 8.83 (d, *J* = 5.7 Hz, 2H), 8.43 – 8.38 (m, 2H), 8.21 (d, *J* = 5.5 Hz, 1H), 7.92 – 7.88 (m, 3H), 7.69 – 7.65 (m, 2H), 7.54 (d, *J* = 7.5 Hz, 2H), 7.31 (d, *J* = 5.0 Hz, 1H), 5.69 (s, 2H). LR-MS: exact mass calculated for $C_{25}H_{19}N_7O$: 433.1651, found 434.3 $(M+1)^+$.

*5-(2-(2-Phenethoxypyridin-4-yl)-1H-pyrrolo[2,3-b]pyridin-4-yl)-1H-indazol-3-amine (**6l**).* The crude residue was purified by HPLC to afford the titled compound as a bright yellow solid (13 mg, 11%). ^{1}H NMR (400 MHz, DMSO-d_6) δ 12.56 – 12.13 (m, 1H), 11.58 (s, 1H), 8.34 (d, *J* = 5.0 Hz, 1H), 8.27 – 8.23 (m, 1H), 8.22 (d, *J* = 5.5 Hz, 1H), 7.74 (dd, *J* = 8.7, 1.6 Hz, 1H), 7.59 (dd, *J* = 5.4, 1.5 Hz, 1H), 7.47 (d, *J* = 1.9 Hz, 1H), 7.44 – 7.39 (m, 2H), 7.37 – 7.30 (m, 4H), 7.25 (dd, *J* = 5.6, 2.1 Hz, 2H), 5.56 (s, 2H), 4.54 (t, *J* = 6.9 Hz, 2H), 3.08 (t, *J* = 6.8 Hz, 2H). LR-MS: exact mass calculated for $C_{27}H_{22}N_6O$: 446.1855, found 446.3 $(M+1)^+$.

S1.1. ^{1}H and ^{13}C NMR spectra for the pharmacological tools **SU1261** [**5r**] and **SU1349** [**6g**]

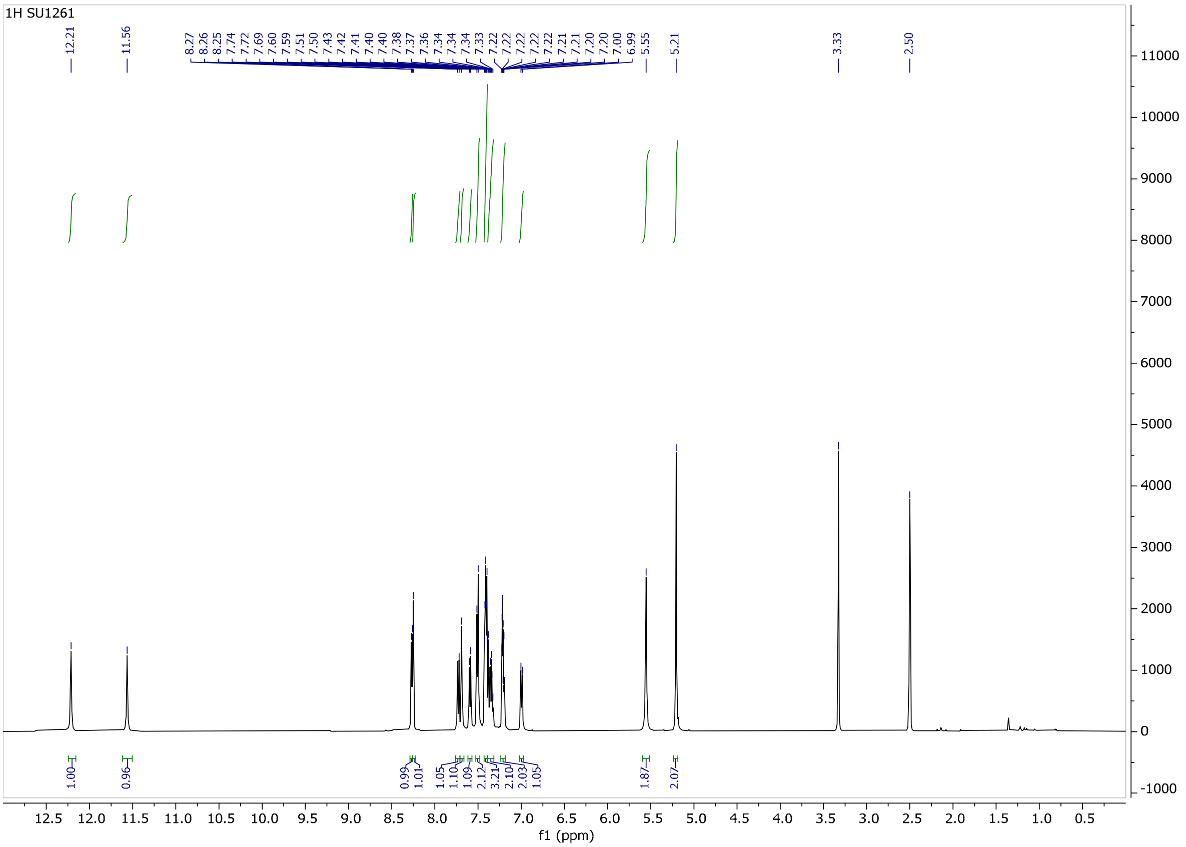

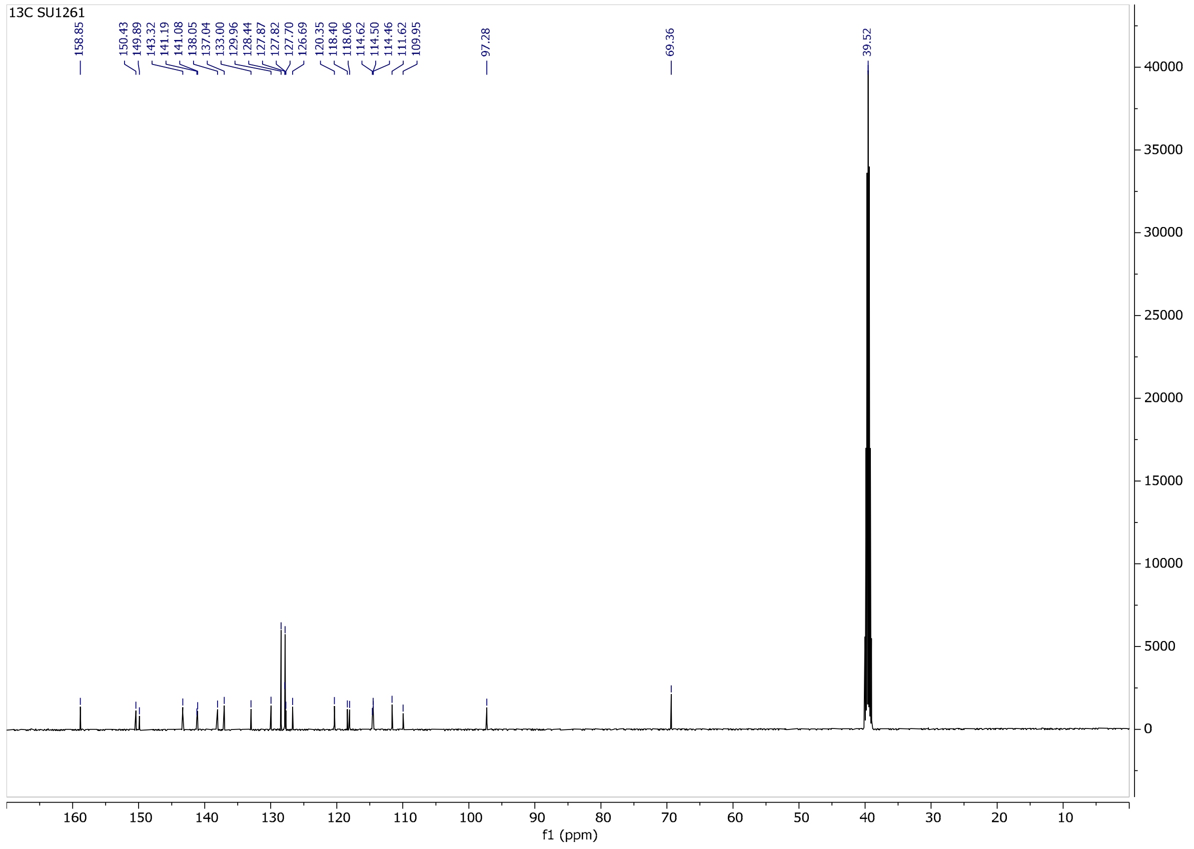
13C SU1261
158.85
150.43
149.89
143.32
141.19
141.08
138.05
137.04
133.00
129.96
128.44
127.87
127.82
127.70
126.69
120.35
118.40
118.06
114.62
114.50
114.46
111.62
109.95
97.28
69.36
39.52
f1 (ppm)

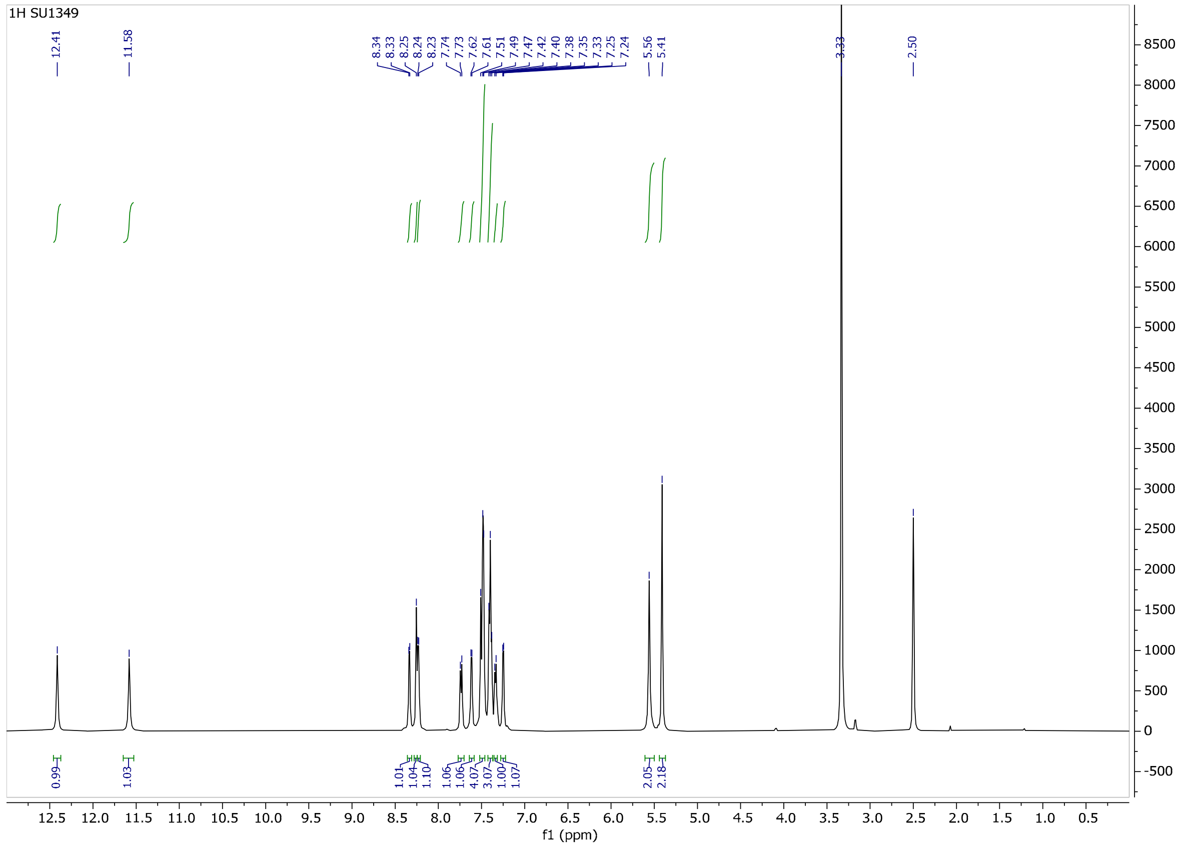
1H SU1349
12.41
11.58
8.34
8.33
8.25
8.24
8.23
7.74
7.73
7.62
7.61
7.51
7.49
7.47
7.42
7.40
7.38
7.35
7.33
7.25
7.24
5.56
5.41
3.33
2.50
0.99
1.03
1.01
1.04
1.10
1.06
1.06
4.07
3.07
1.00
1.07
2.05
2.18
f1 (ppm)

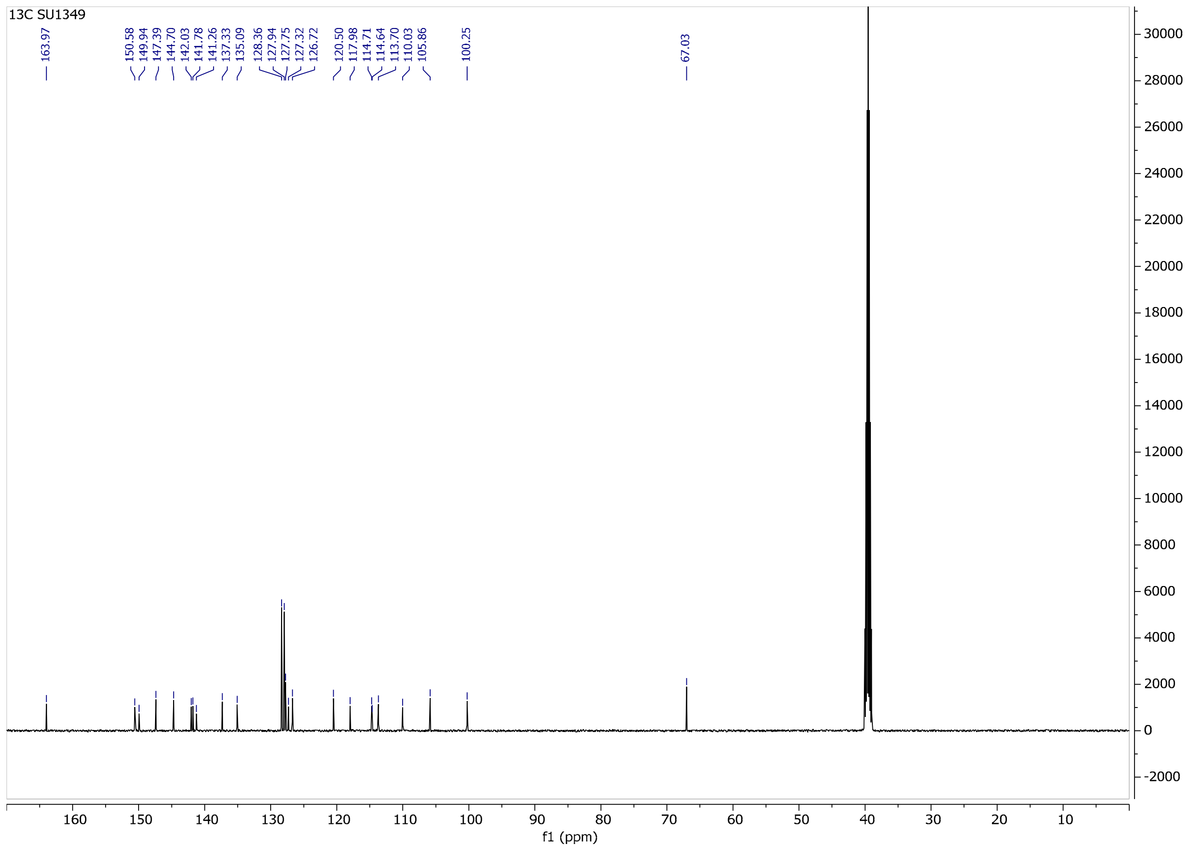
13C SU1349
163.97
150.58
149.94
147.39
144.70
142.03
141.78
141.26
137.33
135.09
128.36
127.94
127.75
127.32
126.72
120.50
117.98
114.71
114.64
113.70
110.03
105.86
100.25
67.03
f1 (ppm)
